# Genetic variants predisposing to an increased risk of kidney stone disease

**DOI:** 10.1172/JCI186915

**Published:** 2025-05-15

**Authors:** Catherine E. Lovegrove, Michelle Goldsworthy, Jeremy Haley, Diane Smelser, Caroline Gorvin, Fadil M. Hannan, Anubha Mahajan, Mohnish Suri, Omid Sadeghi-Alavijeh, Shabbir H. Moochhala, Daniel P. Gale, David Carey, Michael V. Holmes, Dominic Furniss, Rajesh V. Thakker, Sarah A. Howles

**Affiliations:** 1 Nuffield Department of Surgical Sciences, University of Oxford, Oxford, United Kingdom.; 2 Department of Genomic Health, Geisinger Medical Center, Danville, Pennsylvania, USA.; 3 Institute of Metabolism and Systems Research and Centre of Membrane Proteins and Receptors (COMPARE), University of Birmingham, Birmingham, United Kingdom.; 4 Wellcome Centre for Human Genetics, Nuffield Department of Medicine, University of Oxford, Oxford, United Kingdom.; 5 Nottingham University Hospitals NHS Trust, Nottingham, United Kingdom, USA.; 6 UCL Department of Renal Medicine, University College London, London, United Kindom.; 7 Medical Research Council, Integrative Epidemiology Unit, University of Bristol, Bristol, United Kingdom.; 8 Nuffield Department of Orthopaedics, Rheumatology and Musculoskeletal Sciences, University of Oxford, Oxford, United Kingdom.; 9 Academic Endocrine Unit, Radcliffe Department of Medicine, University of Oxford, Oxford, United Kingdom.; 10 National Institute for Health Research Oxford Biomedical Research Centre, Oxford, United Kingdom.; 11 Centre for Endocrinology, William Harvey Research Institute, Barts and the London School of Medicine, Queen Mary University of London, London, United Kingdom.

**Keywords:** Endocrinology, Genetics, Nephrology, Calcium signaling, Genetic variation, Urology

## Abstract

**BACKGROUND:**

Kidney stone disease (KSD) affects approximately 10% of adults, is heritable, and is associated with mineral metabolic abnormalities.

**METHODS:**

Genetic variants and pathways increasing KSD risk via calcium and phosphate homeostasis were ascertained using GWAS, region-specific Mendelian randomization (MR), and genetic colocalization. The utility of pathway modulation was estimated via drug target MR, and the effects of variants on calcium-sensing receptor (CaSR) signaling were characterized.

**RESULTS:**

Seventy-nine independent KSD-associated genetic signals at 71 loci were identified. MR identified 3 loci affecting KSD risk via increased serum calcium or decreased serum phosphate concentrations (ORs for genomic regions = 4.30, 11.42, and 13.83 per 1 SD alteration; *P* < 5.6 × 10^–10^). Colocalization analyses defined putative, noncoding KSD-causing variants estimated to account for 11%–19% of KSD cases in proximity to diacylglycerol kinase δ (*DGKD*), a CaSR signaling partner; solute carrier family 34 member 1 (*SLC34A1*), a renal sodium-phosphate transporter; and cytochrome P450 family 24 subfamily A member 1 (*CYP24A1*)*,* which degrades 1,25-dihydroxyvitamin D. Drug target MR indicated that reducing serum calcium by 0.08 mmol/L via *CASR*, *DGKD*, or *CYP24A1*, or increasing serum phosphate by 0.16 mmol/L via *SLC34A1* may reduce KSD relative risk by up to 90%. Furthermore, reduced DGKδ expression and KSD-associated *DGKD* missense variants impaired CaSR signal transduction in vitro, which was ameliorated by cinacalcet, a positive CaSR allosteric modulator.

**CONCLUSION:**

*DGKD*-, *SLC34A1*-, and *CYP24A1*-associated variants linked to reduced CaSR signal transduction, increased urinary phosphate excretion, and impaired 1,25-dihydroxyvitamin D inactivation, respectively, are common causes of KSD. Genotyping patients with KSD may facilitate personalized KSD risk stratification and targeted pharmacomodulation of associated pathways to prevent KSD.

**FUNDING:**

Oxfordshire Health Services Research Committee (OHSRC, part of Oxford Hospitals Charity); Kidney Research UK (RP_030_20180306); The Urology Foundation; National Institute for Health Research (NIHR) Oxford Biomedical Research Centre (NF-SI-0514–10091); Wellcome Trust (204826/z/16/z and 106995/z/15/z); Medical Research Council (MRC) Clinical Research Training Fellowships (MR/W03168X/1 and MR/S021329/1); Wellcome Trust Clinical Career Development Fellowship; Sir Henry Dale Fellowship, with joint funding by the Wellcome Trust and the Royal Society (224155/Z/21/Z); St. Peter’s Trust for Kidney Bladder and Prostate Research.

## Abstract 2

Kidney stone disease is common and recurrent. In these studies we identify three biologic pathways that underlie ~12-17% of common causes of stone formation and that may facilitate personalised medicine approaches.

## Graphical abstract

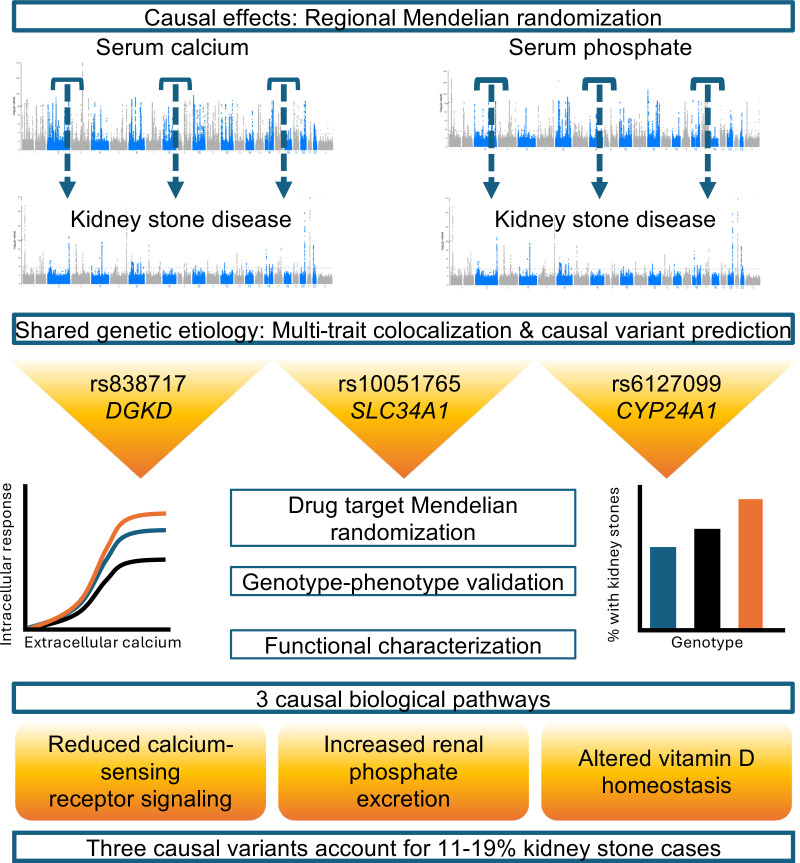

## Introduction

Kidney stone disease (KSD) has a lifetime prevalence of approximately 20% in men and approximately 10% in women and is commonly a recurrent condition (1, 2). Systemic disorders, including disturbances of calcium homeostasis, may predispose individuals to kidney stone formation, and rare monogenic causes of nephrolithiasis are well recognized (3). However, most cases of KSD are considered idiopathic, and multiple genetic and environmental factors contribute to the observed phenotype (4, 5). The homeostatic and renal tubular mechanisms underlying these common forms of KSD are poorly understood, impeding efforts to implement improved therapeutic strategies to prevent recurrent kidney stone formation (6).

Our previous genomic studies revealed that higher serum calcium and lower serum phosphate concentrations likely increase the risk of KSD (7), suggesting that minor perturbations in mineral metabolism within the normal range may be a common risk factor for kidney stone formation. Thus, a 0.08 mmol/L increase in serum calcium was associated with an KSD OR of 1.48, and a 0.16 mmol/L decrease in serum phosphate concentration was associated with an KSD OR of 1.41 (7). To further characterize the mechanisms by which alterations in calcium and phosphate homeostasis contribute to kidney stone formation, we pursued genetic discovery studies, in vitro assays, and 3D modeling to report KSD-causing variants associated with diacylglycerol kinase δ (*DGKD*), which encodes the calcium-sensing receptor (CaSR) signaling partner DGKδ; solute carrier family 34 member 1 (*SLC34A1*), which encodes renal sodium-phosphate transport protein 2A (NaPi-IIa); and cytochrome P450 family 24 subfamily A member 1 (*CYP24A1*), which encodes the 24-hydroxylase that inactivates 1,25-dihydroxyvitamin D.

## Results

### Putative kidney stone causal variants.

**Figure 1:**
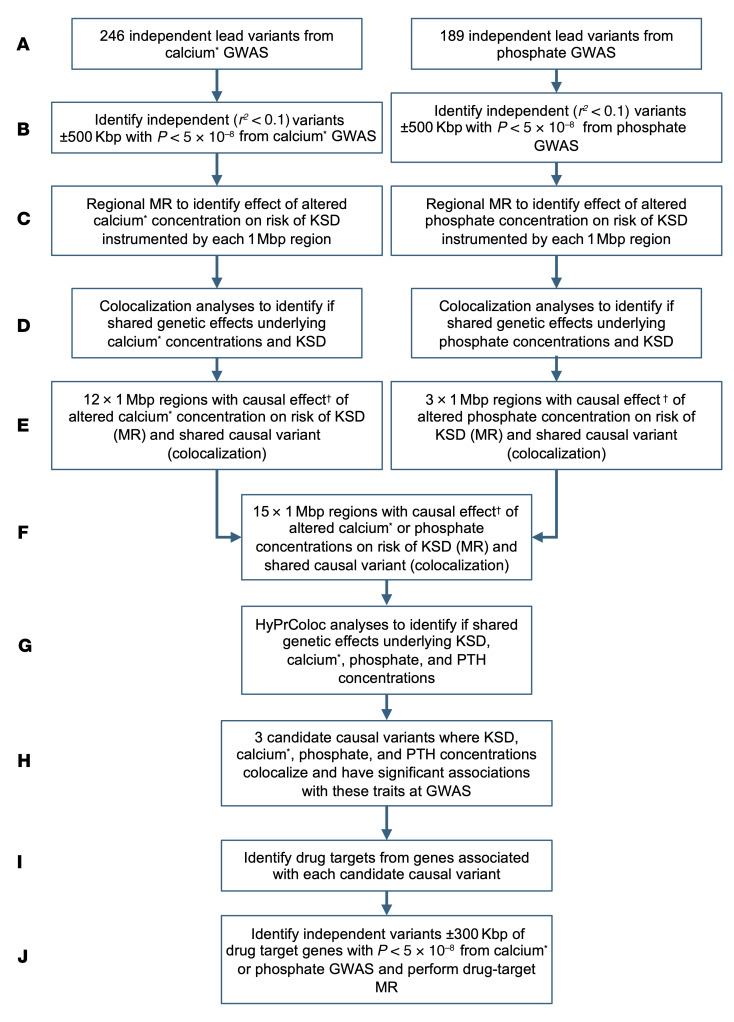
Study design to identify genetic variants predisposing to an increased risk of KSD. (**A** and **B**) Independent (*r^2^* < 0.1) genetic variants ± 500 kbp of the lead independent variants from serum albumin–adjusted calcium or phosphate GWAS significantly (*P* < 5 × 10^–8^) associated with serum albumin–adjusted calcium or phosphate concentrations were selected for use as IVs. (**C**) MR was performed using each of the identified IVs to instrument the effects of alterations in the biochemical exposure on the risk of KSD using UK Biobank, FinnGen, and UK Biobank-FinnGen meta-analysis KSD GWAS summary statistics. (**D**) Colocalization analyses were performed. (**E**) Regions with significant MR results (after *P* value adjustment using the FDR method) and evidence of colocalization were identified. (**F**–**H**) HyPrColoc was undertaken to assess whether there was colocalization between KSD and serum albumin–adjusted serum calcium, phosphate, and PTH concentrations and identity candidate causal variants. (**I**) Drug targets from the genes associated with candidate causal variants were identified. (**J**) Drug target MR was performed to assess the potential utility of modulating drug targets to prevent KSD, selecting genetic variants for use as IVs within 300 kbp of genes of interest. ^*^albumin-adjusted serum calcium concentration; ^†^IV comprising 3 or more genetic variants.

**Figure 2:**
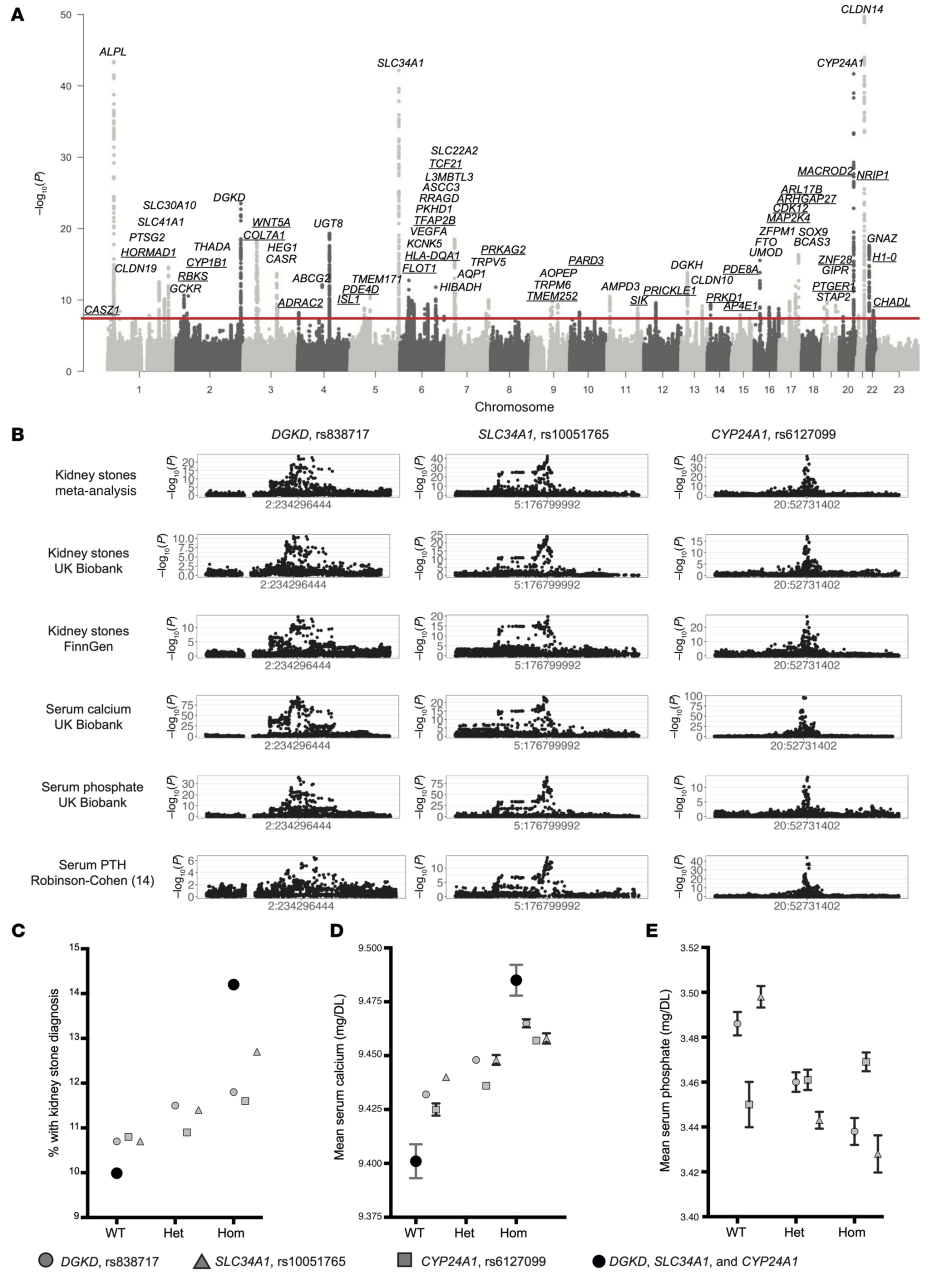
Genetic associations of KSD and serum calcium and phosphate concentrations. (**A**) Meta-analysis of GWASs of data from the UK Biobank and FinnGen including data on 24,167 kidney stone cases and 876,673 controls. Manhattan plot shows genome-wide *P* values (–log_10_) plotted against the chromosomal position. Horizontal red line indicates the genome-wide significance threshold (5.0 × 10^−8^). Loci are labeled with the following primary candidate genes: *CASZ1*, *ALPL*, *CLDN19*, *HORMAD1*, *PTGS2*, *SLC41A1*, *SLC30A10*, *GCKR*, *RBKS*, *CYP1B1*, *THADA*, *DGKD*, *COL7A1*, *WNT5A*, *HEG1*, *CASR*, *ADRAC2*, *ABCG2*, *UGT8*, *ISL1*, *PDE4D*, *TMEM171*, *SLC34A1*, *FLOT1*, *HLA-DQA*, *KCNK5*, *VEGFA*, *TFAP2B*, *PKHD1*, *RRAGD*, *ASCC3*, *L3MBTL3*, *TCF21*, *SLC22A2*, *HIBADH*, *AQP1*, *TRPV5*, *PRKAG2*, *TMEM252*, *TRPM6*, *AOPEP*, *PARD3*, *AMPD3*, *SIK*, *PRICKLE1*, *DGKH*, *CLDN10*, *PRKD1*, *AP4E1*, *PDE8A*, *UMOD*, *FTO*, *ZFPM1*, *MAP2K4*, *CDK12*, *ARHGAP27*, *ARL17B*, *SOX9*, *BCAS3*, *PTGER1*, *STAP2*, *GIPR*, *ZNF28*, *CYP24A1*, *NRIP1*, *CLDN14*, *GNAZ*, *H1-0*, and *CHADL*. Thirty-three of the loci (underlined) have not previously been associated with KSD. (**B**) Locus zooms from GWASs of KSD and albumin-adjusted serum calcium, serum phosphate, and PTH concentrations at loci, with evidence from regional MR that the risk of KSD is increased via serum calcium and phosphate concentrations and where genetic associations of KSD and serum calcium, phosphate, and PTH concentrations colocalize. (**C**–**E**) Associations of genotype with KSD (**C**), serum calcium concentration (**D**), and serum phosphate concentration (**E**) in the DiscovEHR cohort (*n* = 11,451 kidney stone cases and 86,294 controls). Mean serum calcium (**D**) and phosphate (**E**) measurements ± SEM adjusted for KSD case status. Note, in some cases, the SEM is small and obscured by the graphical icon. Associations of combinations of *DGKD*-, *CYP24A1*-, and *SLC34A1*- risk alleles were not assessed for serum phosphate due to a lack of directional concordance. These findings provide evidence that the variants rs838717, rs10051765, and rs6127099 are causal risk factors for KSD acting via reduced CaSR signal transduction, increased urinary phosphate excretion, and impaired vitamin D inactivation, respectively. Het, heterozygous; Hom, homozygous.

To facilitate genetic analyses, a GWAS of data from the UK Biobank, which included 11,186 kidney stone cases and 390,488 controls (Supplemental Figures 1 and 2, and Supplemental Tables 1–3), was undertaken, and a meta-analysis of FinnGen R12 data, which included 12,981 kidney stone cases and 486,185 controls, was performed. This meta-analysis identified 79 independent genetic signals at 71 loci that are associated with KSD (Figures 1 and 2, Supplemental Figure 3, and Supplemental Tables 3 and 4). Thirty-three of these loci have not, to our knowledge, previously been reported to associate with KSD, with candidate genes lying in proximity to the following genes: *CASZ1, CLDN19, HORMAD1, RBKS, CYP1B1, COL7A1, WNT5AA, HEG1, ADRA2C, ISL1, PDE4D, FLOT1, HLA-DQA1, TFAP2B, TCF21, PRKAG2, TMEM252, PARD3, AMPD3, SIK2, PRICKLE1, PRKD1, AP4E1, PDE8A, MAP2K4, CDK12, ARHGAP27, ARL17B, PTGER1, ZNF28, MACROD2, NRIP1, H1-0*, and *CHADL* (Figure 2). Genetic associations with KSD that replicated previous studies were in proximity to the following genes: *ALPL, PTGS2, SLC41A1, SLC30A10, GCKR, THADA, DGKD, CASR, ABCG2, UGT8, TMEM171, SLC34A1, KCNK5, VEGFA, PKHD1, RRAGD, ASCC3, L3MBTL3, SLC22A2, HIBADH, AQP1, TRPV5, TRPM6, AOPEP, AMPD3, DGKH, CLDN10, UMOD, SCNN1B, FTO, ZFPM1, BCAS3, SOX9, STAP2, GIPR, CYP24A1, CLDN14,* and *GNAZ* (Figure 2).

Gene and gene set enrichment analyses revealed associations with 128 protein-coding genes and 10 gene sets, respectively (Supplemental Tables 5 and 6), indicating roles for hypermagnesemia, hypocalciuria, abnormal blood inorganic cation concentration, fibroblast growth factor production, anion homeostasis, urate metabolism, alkaline phosphatase activity, and renal structure and development in KSD.

To ascertain the regional effects of genetically predicted serum calcium and phosphate concentrations on the odds of KSD in the UK Biobank and FinnGen cohorts (8), we used a *cis* Mendelian randomization (MR) approach, systematically considering 1 Mbp genomic areas ± 500 kbp of the lead independent variants associated with serum albumin–adjusted calcium or phosphate in the GWAS (Supplemental Figure 4, Supplemental Data 1) (9, 10). Where potential causal regional effects of serum calcium or phosphate concentrations on KSD were identified, we conducted colocalization analyses to evaluate the probability of a single shared causal variant (11, 12), considering data from KSD GWASs and serum albumin–adjusted calcium, phosphate and parathyroid hormone (PTH) concentration GWASs simultaneously (Figure 1) (10, 13, 14). We identified 3 variants that were significantly associated with kidney stones and predicted to causally increase KSD risk via the effects on serum calcium and phosphate homeostasis (Figures 1 and 2, Supplemental Figure 5, Supplemental Tables 7–13, and Supplemental Data 2 and 3). These variants comprised an intronic *DGKD* variant that is a predicted transcription factor binding site (rs838717) with an MR OR estimate of regional effects on KSD of a 1 SD (0.08 mmol/L) increase in albumin-adjusted serum calcium of 4.51 (95% CI = 3.38–6.03; posterior probability [PP] that the SNP is causal variant = 1.00); an intergenic variant approximately 6 kb upstream of *SLC34A1* (rs10051765) with an MR OR estimate of regional effects on KSD of a 1SD (0.16 mmol/L) decrease in serum phosphate of 11.11 (95% CI = 7.69–14.29, SNP PP = 1.00); and an intergenic variant approximately 50 kb downstream of *CYP24A1* (rs6127099) with an MR OR estimate of regional effects on KSD of a 1 SD increase in albumin-adjusted serum calcium of 10.36 (95% CI = 8.54–12.56, SNP PP = 1.00), using UK Biobank and FinnGen meta-analysis data (Supplemental Tables 8–12).

Mutations of *CYP24A1* and *SLC34A1* are known to cause infantile hypercalcemia (IH) types 1 and 2, respectively (15, 16), which are autosomal recessive disorders of calcium and phosphate metabolism associated with nephrocalcinosis and KSD. IH1 is due to impaired inactivation of 1,25-dihydroxyvitamin D, which leads to elevations in circulating 1,25-dihydroxyvitamin D that result in increased intestinal and renal absorption of calcium with consequent hypercalcemia. In contrast, IH2 is caused by increased renal phosphate excretion due to impaired NaPi-IIa function, resulting in a reduction in serum FGF23 concentrations, activation of 1-α hydroxylase (an enzyme that activates 25-hydroxyvitamin D), and inhibition of 24-hydroxylase. In addition, a reduction in DGKδ expression results in impaired CaSR signal transduction (17), and gain- and loss-of-function mutations in components of the CaSR signaling pathway cause autosomal dominant hypocalcemia with relative hypercalciuria and hyperphosphatemia and familial hypocalciuric hypercalcemia (FHH), which may associate with hypophosphatemia, respectively (18, 19). We sought associations of genotype with serum biochemistry in the DiscovEHR cohort and using UK Biobank GWAS data. Our results revealed that the predicted *DGKD* (rs838717) and *SLC34A1* (rs10051765) causal variants were associated with higher serum calcium and lower serum phosphate concentrations, which are consistent with attenuated forms of FHH and IH2, respectively, and that the predicted *CYP24A1* (rs6127099) causal variant was associated with higher serum calcium concentration without a reduction in phosphate concentration, consistent with an attenuated form of IH1 (Figure 2 and Supplemental Tables 13 and 14). Moreover, the *SLC34A1*-associated candidate causal variant (rs10051765) was highly correlated (*r²* = 0.84) with rs12654812, which has been reported to associate with KSD and lower serum phosphate and PTH concentrations (20).

To determine the clinical relevance of these variants in conferring a risk of developing KSD, we calculated the fraction of KSD that may arise as a result of these 3 putative causal variants in DiscovEHR and UK Biobank. This revealed a population-attributable fraction of approximately 11% in DiscovEHR and approximately 19% in UK Biobank (Supplemental Tables 15 and 16). We did not consult a FinnGenn study for our analysis due to a lack of individual-level data. Furthermore, addition of a single *DGKD*, *SLC34A1*, or *CYP24A1* putative causal variant was associated with a 6%–10%, 10%–16%, and 5%–14% increased risk of KSD, respectively (OR 1.06 and 1.10, 1.10 and 1.16, and 1.05 and 1.14, Supplemental Table 14). Occurrence of all 6 risk alleles in an individual was associated with an approximately 4% increased prevalence (Figure 2) and a greater than 35% increased odds of KSD (Supplemental Tables 14–16; OR = 1.07 for addition of one risk allele). Thus, *DGKD*, *SLC34A1*, and *CYP24A1* variants confer risks that summate to a substantially increased risk of developing KSD. Moreover, these findings indicate that reduced CaSR signal transduction, increased urinary phosphate excretion, and impaired vitamin D inactivation may be common causes of KSD.

### Drug target MR.

**Figure 3:**
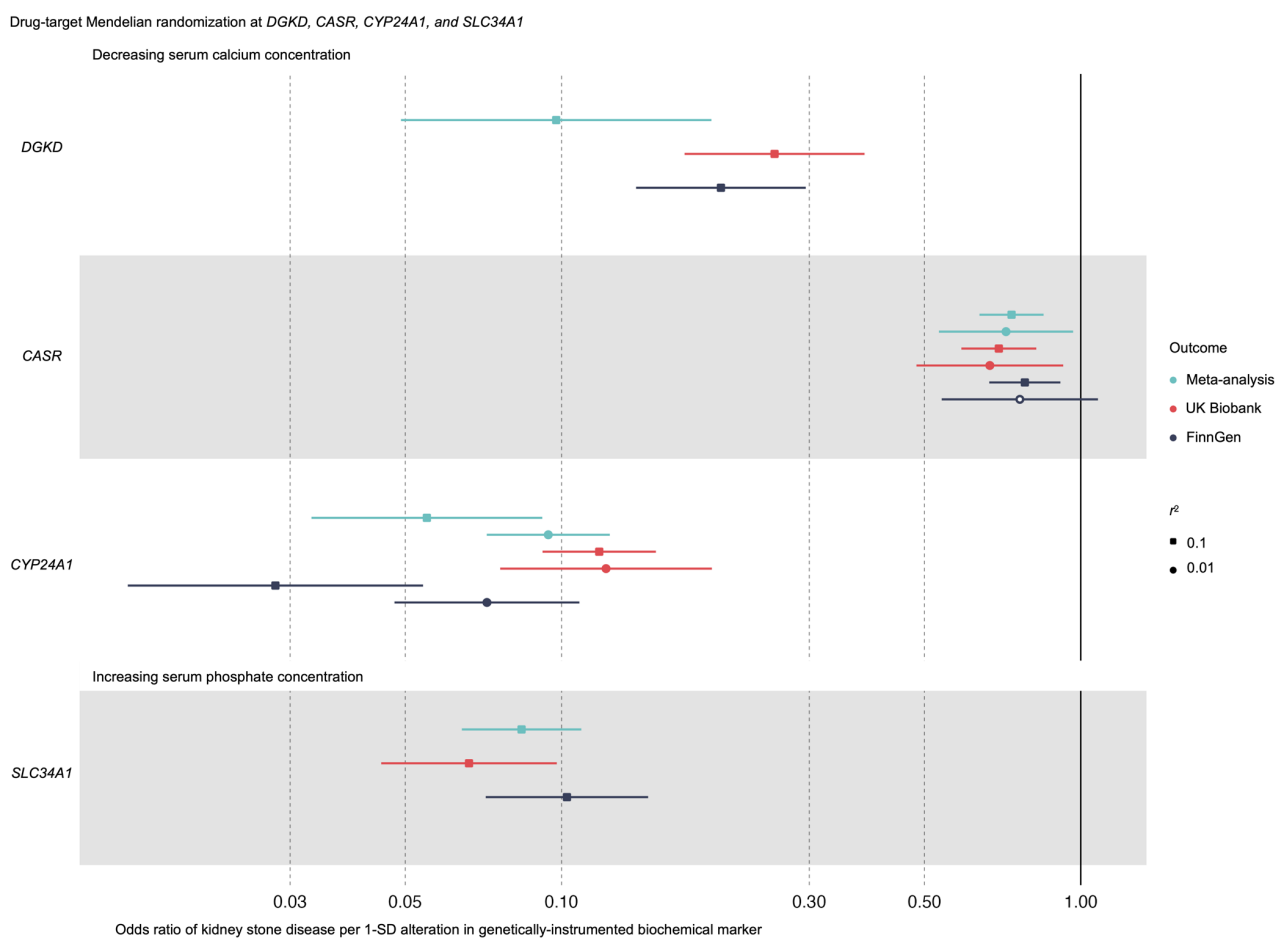
Drug target MR. Forest plot of the predicted effects of modulating albumin-adjusted serum calcium concentrations via *DGKD*, *CASR*, or *CYP24A1* or serum phosphate concentrations via *SLC34A1*. Gene positions are defined via Ensembl ± 300 kbp. There were insufficient genetic instruments to undertake analyses of modulating serum calcium or phosphate concentrations via *DGKD* or *SLC34A1* using a threshold for genetic independence (*r^2^*) of 0.01. These data indicate that reducing serum calcium via *DGKD*, *CASR*, or *CYP24A1*, or increasing serum phosphate via *SLC34A1* would decrease the risk of KSD.

To identify potential therapeutic pathways that could be modulated to prevent KSD, we undertook drug target MR analyses using a stringent threshold of *r^2^* < 0.01 to define independent genetic variants as exposure instrumental variables (IVs). GWAS summary statistics from studies using data from the UK Biobank and FinnGen and meta-analyzed results were used as outcome datasets (Figure 1 and Supplemental Figure 4). These analyses suggested that modulating *CASR* and *CYP24A1* to reduce serum calcium concentrations by 1 SD (0.08 mmol/L) may decrease KSD relative risk by approximately 30% and approximately 90%, respectively (Figure 3 and Supplemental Table 17); directionally concordant but statistically insignificant effects were detected for *CASR*-mediated effects using FinnGen outcome data (Figure 3 and Supplemental Table 17). Similar analyses of *DGKD* or *SLC34A1* modulation were not possible, as there were insufficient genetic proxies. However, MR analyses relaxing the threshold of genetic independence of IVs to *r^2^* < 0.1 indicated that reducing serum calcium concentrations by 1 SD via *DGKD* may decrease the risk of KSD by approximately 70%, and increasing serum phosphate concentrations by 1 SD (0.16 mmol/L) via *SLC34A1* may decrease the risk of KSD by more than 90% (Figure 3 and Supplemental Table 17). Phenome-wide association study data suggested that modulating *DGKD*, *CASR*, *CYP24A1*, or *SLC34A1* may result in target-mediated adverse effects including alterations in serum bilirubin, inflammatory bowel disease, migraine, atopic dermatitis, and eczematous phenotypes (Supplemental Data 4) (9).

### Coding region DGKD variants associated with KSD.

**Figure 4:**
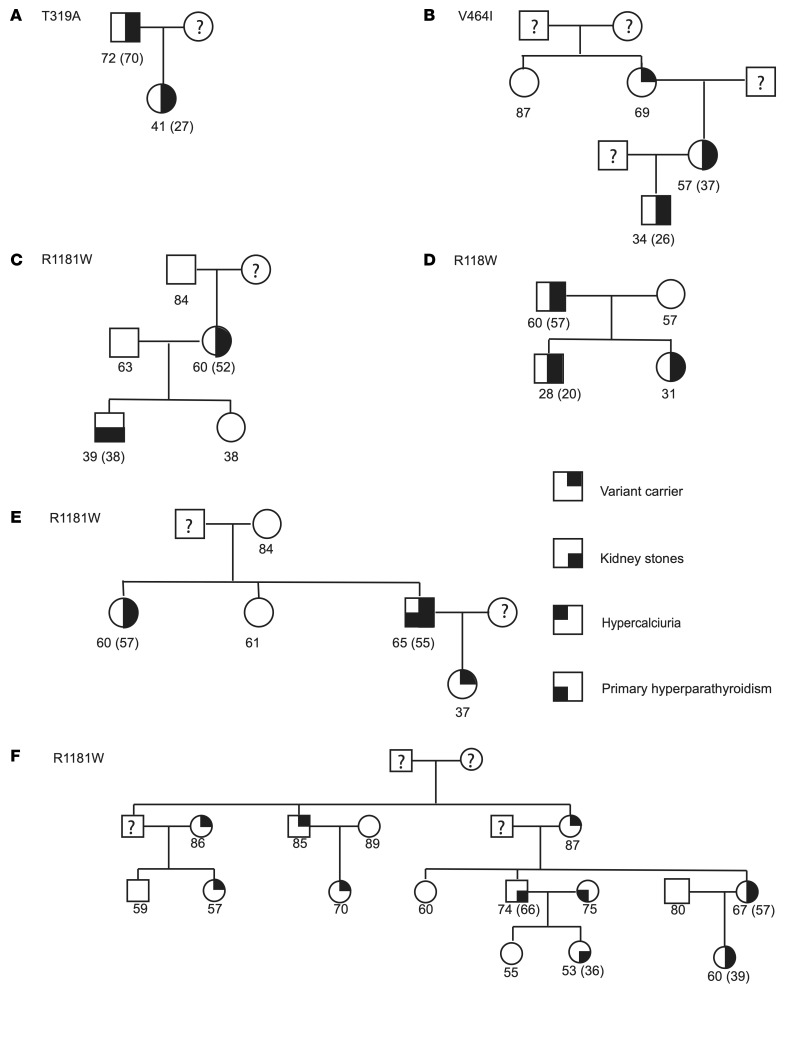
Family trees of DiscovEHR kindreds (A–F) were identified as harboring *DGKD* variants. Squares represent male family members, circles female family members, and ? indicates missing data. Individuals’ ages (years) are shown below the symbols, and the age of the individual at the first record of a kidney stone episode is shown in parentheses.

The function of *SLC34A1* and *CYP24A1* in mineral metabolism and IH are well characterized (15, 21), so we therefore focused on further defining the role of DGKδ in CaSR signaling and KSD. A total of 7 rare, predicted deleterious *DGKD* variants associated with KSD were identified in the Genomics England 100,000 Genomes Project (100KGP) and DiscovEHR cohorts (2 from the 100KGP [H190Q and I221N]; 4 from DiscovEHR [I91V, T319A, V464I, R900H]; and 1 [R1181W] from both cohorts) (Supplemental Tables 18 and 19). Residues I91, H190, I221, T319, R900, and R1181 are evolutionarily conserved and residue V464 is partially conserved across vertebrate DGKδ orthologs, suggesting that these DGKδ variants may be pathogenic (Supplemental Figure 6). In DiscovEHR, 6 kindreds with *DGKD* variants comprised 13 individuals who were variant carriers and affected with a relevant phenotype (11 KSD, 1 hypercalciuria, and 1 primary hyperparathyroidism and KSD); 7 individuals who were variant carriers but unaffected; 12 individuals who were not variant carriers and unaffected; and 3 individuals who were not variant carriers but affected with KSD (*n* = 2) or primary hyperparathyroidism (*n* = 1) (Figure 4). Variants T319A and V464I cosegregated with KSD in 2 DiscovEHR cohort kindreds, but penetrance was incomplete for V464I (Figure 4). Statistically significant associations of R1181W with KSD were not detected in DiscovEHR, however, in 3 kindreds, R1181W cosegregated with KSD with incomplete penetrance, and in a further kindred, cosegregation was incomplete with the possibility of additional genetic risk factors for KSD (Figure 4).

### Functional characterization of DGKδ variants.

**Figure 5:**
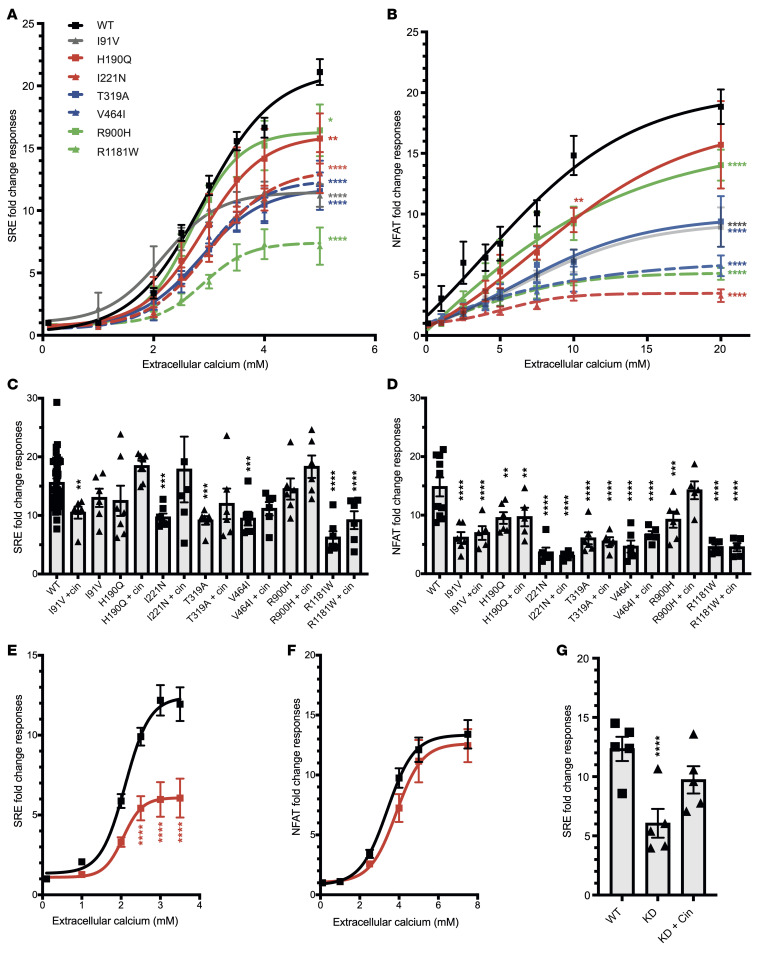
Functional characterization of kidney stone–associated DGKδ variants. (**A**) CaSR-mediated SRE and (**B**) NFAT-RE responses to changes in extracellular calcium concentration [Ca^2+^]_e_ in HEK-CaSR-DGK cells stably transfected with WT or the kidney stone–associated variants I91V, H190Q, I221N, T319A, V464I, R900H, or R1181W. Transfection with kidney stone–associated *DGKD* variants led to a reduction in SRE and NFAT-RE responses compared with cells transfected with WT *DGKD*. (**C**) Effect of 100 nM cinacalcet (cin) treatment on SRE responses at 3.5 mM [Ca^2+^]_e_ and (**D**) NFAT-RE responses at 10 mM [Ca^2+^]_e_ in HEK-CaSR-DGK cells transfected with the kidney stone–associated variants. Treatment with cinacalcet increased SRE-mediated responses of all variants but had no effect on NFAT-RE responses except for cells transfected with the R900H variant. (**E**) CaSR-mediated SRE and (**F**) NFAT-RE responses to changes in [Ca^2+^]_e_ in HEK-CaSR cells following DGKδ KD (red), which led to a reduction in SRE responses without a change in NFAT-RE responses, compared with WT (black). (**G**) Effect of 5 nM cinacalcet treatment on SRE responses at 3.5 mM [Ca^2+^]_e_ in HEK-CaSR cells following DGKδ KD. Treatment with cinacalcet rectified impaired SRE-mediated responses. Mean fold change responses ± SEM are shown for 4 biologically independent experiments. A 2-way ANOVA with Dunnett’s correction for multiple comparisons was used to compare points on the dose response curve with reference to WT. These data provide evidence that KSD is associated with impaired CaSR signal transduction, which can be ameliorated with cinacalcet. *P < 0.05, **P < 0.01, ***P < 0.001, and ****P < 0.0001 versus WT.

We assessed the effects of KSD-associated *DGKD* variants and reduced DGKδ expression on CaSR signal transduction in CaSR-expressing HEK293 cells and determined their responses to alterations in extracellular calcium concentration using ERK phosphorylation (pERK) and serum response element (SRE) assays to evaluate Ras/Raf/MEK/ERK signaling and NFAT-RE assays to evaluate intracellular calcium release (Supplemental Figure 7). Variants I91V, H190Q, I221N, T319A, V464I, R900H, and R1181W resulted in reduced pERK and SRE responses and/or reduced NFAT-RE–mediated responses in comparison with cells transfected with WT DGKδ, and reduced DGKδ expression, resulting from shRNA *DGKD* knockdown (KD), attenuated pERK and SRE-mediated responses without a change in NFAT-mediated responses (Figure 5 and Supplemental Figures 8 and 9). These findings are consistent with loss-of-function mutations in components of the CaSR signaling pathway and indicate that DGKδ KD resulted in biased CaSR signal transduction. The CaSR positive allosteric modulator cinacalcet rectified the CaSR signaling loss-of-function effects associated with reduced DGKδ expression and ameliorated impaired SRE responses due to DGKδ KSD–associated variants (Figure 5). However, cinacalcet had no effect on impaired NFAT-RE responses due to DGKδ KSD–associated variants, except for the mildly inactivating R900H (Figure 5 and Supplemental Figure 9).

### Predicted effects of DGKδ variants on protein function.

**Figure 6:**
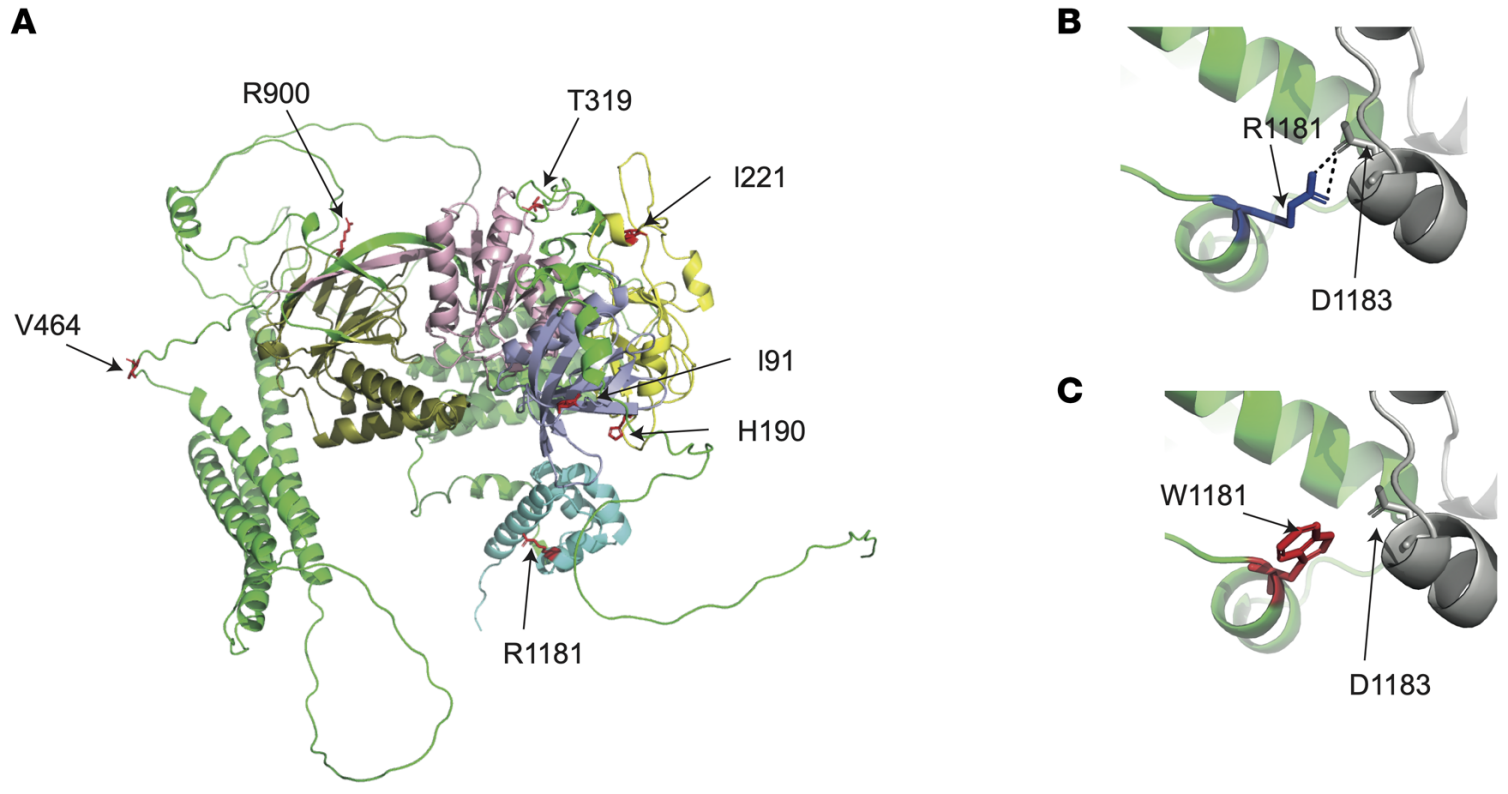
3D modeling of kidney stone–associated DGKδ variants. (**A**) Predicted structure of DGKδ isoform 2 (AF-Q16760-F1-mod; refs. 79, 80; AlphaFold). Residue I91 lies in the pleckstrin homology domain (purple); residues H190 and I221 are located in the cysteine-rich domain (yellow); residue T319 is in the catalytic domain (pink); residue V464 is in a linker region; residue R900 is in the accessory catalytic domain (dark green); and R1181 is located in the SAM domain (blue) (86). (**B**) Location of R1181 (dark blue) in the oligomeric DGKδ SAM domain crystal structure (PDB 3BQ7; ref. 25). R1181 is predicted to form a polar contact (dashed black line) with D1183 on the adjacent DGKδ SAM domain (gray). (**C**) Location of W1181 (red) in the oligomeric DGKδ SAM domain crystal structure. W1181 is not predicted to form a polar contact with D1183 on the adjacent DGKδ SAM structure (gray).

To further elucidate the mechanisms by which KSD-associated DGKδ variants may alter DGKδ function, we pursued 3D modeling studies. DGKδ R1181 is in the sterile α motif (SAM) domain, which facilitates DGKδ oligomerization and intracellular localization (22). Analysis of the crystal structure of oligomeric DGKδ SAM domains indicated that R1181 likely forms a polar contact with D1183 on adjacent DGKδ SAM domains. Replacing the polar R1181 residue with a nonpolar W1181 residue is predicted to cause a reduced affinity for the adjacent DGKδ SAM domain, which may compromise DGKδ oligomerization and alter intracellular localization (Figure 6). Mutation of D1183 to G1183 is reported to increase DGKδ solubility in vitro, indicating a reduction in oligomerization, and to induce spontaneous localization of DGKδ to the plasma membrane (23). As the crystal structure of the remainder of DGKδ has not been solved, we undertook additional analyses using the AlphaFold DGKδ-predicted structure. This analysis revealed that DGKδ I91 is within the pleckstrin homology domain and that the V91 variant may impair DGKδ binding to partner proteins and cell membrane localization (24, 25); that H190 and I221 are within the C1 domain of DGKδ, and the Q190 and N221 variants may affect diacylglycerol (DAG) binding (26); and that T319 and R900 are located in proximity to the ATP-binding pocket and in the accessory domain ATP-binding motif, respectively, and the A319 and H900 variants may alter ATP-binding dynamics (Supplemental Figure 10). Predictions regarding the mechanistic effects of V464I could not be made because of its location in a region of the AlphaFold structure with very low model confidence.

## Discussion

To our knowledge, this study is the first to use a systematic, region-specific MR approach, combined with colocalization analyses, to identify putative disease-causing genetic variants and pathways. Using this approach, we identified 3 common, noncoding variants that predict an increased risk of KSD via DGKδ-mediated reduced CaSR signal transduction, NaPi-IIa–mediated increased renal phosphate excretion, and 24-hydroxylase–mediated decreased 1,25-dihydroxyvitamin D inactivation. Furthermore, we found that these 3 variants may be responsible for 11%–19% of KSD cases and that homozygosity for all putative causal variants was associated with greater than 35% increased odds and approximately 4% increased prevalence of kidney stones. Our findings highlight the importance of increased serum calcium concentrations arising from attenuated CaSR signal transduction, altered vitamin D homeostasis, and increased urinary phosphate excretion via effects on FGF23, 1-α hydroxylase, and 24 hydroxylase in the pathogenesis of common idiopathic forms of KSD as well as in the rare monogenic kidney stone–associated diseases FHH, IH1, and IH2, respectively.

These results may have direct clinical utility in facilitating the prediction of kidney stone recurrence risk and thereby motivate lifestyle modifications and the selection of therapeutic interventions such as calcimimetics to ameliorate DGKδ-mediated CaSR signaling perturbations; phosphate supplements to increase serum phosphate in NaPi-IIa–associated KSD; or inhibitors of vitamin D activation (for example triazole drugs or rifampicin) and/or avoidance of vitamin D supplementation, in which 24-hydroxylase–mediated 1,25-dihydroxyvitamin D inactivation is likely impaired. Indeed, our drug target MR studies indicate that modulating DGKδ, CaSR, or 24-hydroxylase to decrease serum calcium or NaPi-IIa to increase serum phosphate is predicted to decrease KSD risk by up to 90%. Furthermore, we demonstrate that cinacalcet, a positive CaSR allosteric modulator, ameliorated impaired CaSR-mediated signaling due to rare kidney stone–associated DGKδ missense mutations and normalized biased CaSR signal transduction in DGKδ-depleted cells. Idiopathic hypercalciuria occurs in up to 50% of individuals with KSD (27), and negative CaSR allosteric modulators have been suggested as a potential therapy to reduce kidney stones in these individuals by decreasing urinary calcium excretion (28). However, our studies indicate that increased serum calcium concentrations may have an underappreciated role in KSD that is independent of urinary calcium excretion, and that the use of negative CaSR allosteric modulators, which would be expected to increase serum calcium concentrations, may increase the risk of kidney stones. Although positive allosteric modulation of the CaSR might be expected to increase urinary calcium concentrations and therefore kidney stone risk, it is interesting to note that increased urinary calcium excretion is not always reported when cinacalcet is used to treat disorders of impaired CaSR signaling (29–31), thus suggesting that treatment with a positive allosteric modulator such as cinacalcet may possibly be of benefit to patients with kidney stones. Further translational studies are required to assess the therapeutic potential of calcimimetics, phosphate supplementation, and inhibitors of vitamin D activation in the context of KSD, with careful attention to the effects on urinary mineral concentrations, given that increasing urinary calcium or phosphate excretion may exacerbate kidney stone risk.

Both FHH and autosomal dominant hypocalcemia are reported to associate with KSD (32–35), although kidney stones most commonly occur in the context of autosomal dominant hypocalcemia with hypercalciuria. Our current study reveals pathways linking the *DGKD*-associated predicted transcription factor binding site rs838717 to KSD via increased serum calcium concentrations, a phenotype in keeping with decreased CaSR signal transduction and FHH. Furthermore, we found that rs838717 associated with decreased phosphate concentrations, which is compatible with the CaSR acting as a phosphate sensor to regulate PTH release (36). Moreover, our functional studies of rare coding, KSD-associated *DGKD* variants are consistent with DGKδ-mediated impaired CaSR signal transduction in the pathophysiology of nephrolithiasis. Kidney stones, parathyroid hyperplasia, mild hypercalcemia, and hypercalciuria have been described in a kindred with the FHH-associated CaSR loss-of-function mutation F881L (32). This is similar to our findings of hyperparathyroidism in individuals with KSD carrying the DGKδ Q190 and W1181 variants. Furthermore, DGKδ W1181 was associated with hypercalciuria in this study, and rs838717 has been reported to be associated with higher urinary calcium excretion in a small cohort (17), although associations of 24-hour urinary calcium excretion and rs838717 were not replicated in a GWAS of approximately 6,500 individuals (37). Thus, DGKδ-associated perturbations of CaSR signal transduction may have intracellular and tissue-specific effects that mirror CaSR loss-of-function variants associated with hyperparathyroidism phenotypes (32, 38).

Idiopathic calcium oxalate stones may form on subepithelial renal papilla calcium phosphate aggregates known as Randall’s plaques. Randall’s plaques are hypothesized to arise due to high levels of calcium reabsorption in the thin ascending limb of the loop of Henle, leading to increased calcium loads in the descending vasa recta that raise supersaturations sufficiently to cause mineral precipitation (39). A tendency toward higher serum and urinary calcium concentrations due to altered CaSR signaling that mirrors CaSR-associated hyperparathyroidism phenotypes (32, 38) would act in tandem to increase the likelihood of plaque formation and may provide mechanistic insight into the occurrence of Randall’s plaques and kidney stones in the absence of overt hypercalcemia or hypercalciuria.

Our findings of impaired DGKδ-mediated CaSR NFAT-RE responses that were not ameliorated by cinacalcet and arose due to DGKδ missense variants may be due to reduced phosphatidylinositol availability as a result of decreased phosphatidic acid production or provide evidence for diacylglycerol (DAG) and inositol 1,4,5-trisphosphate crosstalk (40). Furthermore, our study reveals that DGKδ missense variants impaired both NFAT-RE and pERK/SRE CaSR signaling responses, while reduced DGKδ expression impaired only CaSR pERK/SRE signaling, highlighting the complexity of DGKδ-mediated perturbations of CaSR signal transduction. In addition, our studies indicate the importance of DGKδ oligomerization, ATP binding, pleckstrin homology, and cysteine-rich domains in DGKδ function. Monoallelic *SLC34A3* variants likely increase kidney stone risk via effects that are insufficient to cause fully penetrant Mendelian disease but confer a higher risk than aggregate effects of known common risk alleles (41). The identification of incompletely penetrant DGKδ variants in this study, including the recurrent R1181W, which is reported 4 times in a homozygous state in the genome aggregation database gnomAD, suggests that *DGKD* variants may fall into a comparable intermediate-risk category.

As part of our causal variant discovery pipeline, we undertook the largest GWAS of KSD to date, including data from 24,167 kidney stone cases and 876,673 controls. This GWAS identified 33 loci, which, to our knowledge, have not previously been associated with KSD, and facilitated gene set analyses that highlight the potential role of altered calcium, magnesium, inorganic blood cation concentrations, and urate metabolism in KSD. Furthermore, several disease-associated loci underscore the importance of renal tubular calcium and magnesium transport in common forms of KSD, including *TRPV5*, which encodes the transient receptor potential cation channel subfamily V protein that facilitates calcium reabsorption in the distal tubule (42), *CLDN10*, *CLDN14*, and *CLDN19*, which encode claudins 10, 14, and 19, respectively, and act with claudin 16 to maintain tight junctions in the thick ascending limb of the loop of Henle to modulate paracellular divalent cation reabsorption, and *TRPM6*, which encodes the transient receptor potential cation channel 6 that has a role in magnesium homeostasis in the distal tubule. Biallelic loss-of-function mutations in *CLDN19* are known to cause familial hypomagnesemia with hypercalciuria and nephrocalcinosis. Our study is the first, to our knowledge, to link a common *CLDN19*-associated variant with KSD.

Our findings are most likely relevant to calcium oxalate and calcium phosphate lithiasis rather than uric acid stones, which comprise only approximately 10% of all kidney stones and most commonly form in the context of reduced urinary pH rather than perturbed mineral metabolism. Our gene set analyses highlighted a potential role for urate metabolism in KSD in these studies; we predict this reflects the effects of urinary uric acid on calcium oxalate stone formation (43).

The use of databases that primarily include data on individuals of White European descent may be a limitation in the generalizability of our findings. However, the genetic architecture of KSD has previously been reported to be similar across diverse populations (17), thus suggesting that our findings may have a more general relevance, although this remains to be proven. Our study is further limited by the lack of suitable genetic proxies of urinary calcium and phosphate excretion to facilitate a more detailed exploration of mediating biological pathways and examine the potential role of pleiotropy related to urinary phenotypes. Moreover, additional studies are required to define the cells and tissues via which impaired DGKδ-mediated CaSR signal transduction causes increased kidney stone risk. Our studies, which have not examined the physiological effects of DGKδ dysfunction, indicate that additional investigations are required to define the cells and tissues via which impaired DGKδ-mediated signal transduction via the CaSR and other GPCRs may cause increased kidney stone risk. DGKδ is a ubiquitously expressed protein, and its coexpression with the CaSR in the parathyroid glands is in keeping with the reported observation of an association of the *DGKD*-associated causal variant rs838717 with increased PTH serum concentrations (13). In addition to these DGKδ-mediated parathyroid effects, rs838717 may also exert effects on signaling by another GPCR, namely the PTH1 receptor (*PTH1R*) in the proximal renal tubule, where both *DGKD* and *PTH1R* are highly expressed (44); this would be consistent with our finding that rs838717 is associated with reduced serum phosphate and increased serum calcium concentrations. In vivo studies in *Dgkd*-mutant mice and patients with *DGKD* variants and KSD may help to further elucidate of the physiological effects of DGKδ dysfunction on these GPCR signaling pathways.

In conclusion, this study defines 3 common putative KSD-causing variants and demonstrates the central role of the DGKδ-mediated reduction of CaSR signal transduction, increased renal phosphate excretion, and perturbed 1,25 vitamin D inactivation in kidney stone pathogenesis. Our studies reveal potential novel therapeutic approaches for kidney stone prophylaxis and may have clinical utility in enabling personalized risk stratification and management strategies in KSD.

## Methods

### Sex as a biological variable

Participants were of the male and female sexes. Sex was used as a covariate in GWAS analyses. Associations in the DiscovEHR cohort reflect combined-sex analyses.

### KSD GWAS and meta-analysis

A GWAS of KSD, including genetic sex and genotyping platform as covariates, was undertaken with UK Biobank data (45) using a linear mixed noninfinitesimal model (BOLT-LMM, version 2.4) to account for population substructure and cryptic relatedness (46). Kidney stone cases were identified using International Classification of Diseases (ICD) revisions 9 and 10, Office of Population Censuses and Surveys Classification of Surgical Operations and Procedures (OPCS) revisions 3 and 4, primary care Read v2 and v3 codes, and self-report codes (Supplemental Table 1). Genotype phasing and imputation in the UK Biobank study using UK-BiLEVE and UK-Biobank Axiom Arrays has been previously described (47). This included 92,693,895 autosomal SNPs, short indels, and large structural variants. R version 4.2.0 and PLINK, version 2.0, were used for quality control (QC). SNPs with a call rate below 90% were removed, accounting for the 2 different genotyping platforms used to genotype individuals. SNP-level QC excluded SNPs with Hardy-Weinberg equilibrium *P* of less than 10^−4^, a call rate of less than 98%, and a minor allele frequency (MAF) of less than 1%. Following QC, data from 547,011 autosomal genotyped and 8,397,548 imputed variants were considered.

An hg19 reference genetic map and a reference linkage disequilibrium (LD) score file for European ancestry were used. Quantile-quantile and Manhattan plots were generated using the “qqman” package, implemented in R (48). Heritability estimates were calculated using Linkage Disequilibrium Score Regression (LDSC), version 1.0.1 (49, 50). Analyses were restricted to variants in HapMap3 (51) and used LD scores computed with 1000 Genomes European data (49, 50, 52). A population prevalence approximation of 10% was used in liability transformation. Genome-Wide Complex Trait Analysis (GCTA) software, version 1.94.1, was used to perform step-wise approximate conditional and joint analysis with the same UK Biobank LD reference panel used in the UK Biobank KSD GWAS (53, 54). Where there was a single signal of association at a locus (a chromosomal region with adjacent pairs of KSD-associated SNPs of less than 1 Mbp apart; refs. 53, 55), the index SNP was defined as the lead SNP from unconditional analysis. For loci with multiple association signals, the index SNP was defined as that with the lowest *P* value in conditional approximate analysis.

Novel loci were defined as those associated with kidney stones at a threshold *P* value of less than 5 × 10^−8^ and, to our knowledge, not reported, or within 1 Mbp of a variant/independent locus significantly associated with kidney stones in previously published GWASs.

A fixed-effects meta-analysis of KSD was performed using UK Biobank and FinnGen kidney stone GWAS summary statistics. FinnGen R12 GWAS data are publicly available for the phenotype “N14 calculus of kidney and ureter” comprising 12,999 cases and 486,185 controls (56). Information on sample phenotyping, genotyping, and GWAS in the FinnGen sample has been given previously (56). SNPs with a MAF of less than 0.01 were omitted from the FinnGen summary statistics. Meta-analysis was undertaken in METAL and effect sizes weighted using the inverse of corresponding standard errors (57). SNPs with a high level of heterogeneity between studies (*I*^2^ statistic >75%) were excluded. The resulting summary statistics were used to perform MR and colocalization analyses.

### Gene and gene set analyses

Genes and gene sets associated with KSD were identified in the UK Biobank-FinnGen meta-analysis using Multi-marker Analysis of GenoMic Annotation (MAGMA), version 1.10 (58, 59). MAGMA uses a multiple regression model to incorporate variant-level *P* values, LD, and gene positions to detect multi-SNP effects (58). Gene associations were determined using a SNP-wise mean model and LD patterns based on 1,000 Genomes Project genotypes. A Bonferroni-adjusted *P* value of greater than 0.05/*N* provides evidence against the null hypothesis, where *N* refers to the number of genes tested. For gene set and cell-based analyses, Human Phenotype Ontology (HPO) and Gene Ontology (GO) term-based gene sets and cell-type annotations were downloaded from the Molecular Signature Database (MSigDB), version 2022.1 (https://www.gsea-msigdb.org/gsea/msigdb/collections.jsp) (59–61). Bonferroni-corrected *P* value thresholds were used to identify HPO gene sets or cell types and genes within these sets significantly associated with KSD (*P* < 0.05/number of gene sets tested and *P* < 0.05/number of genes in the gene set, respectively).

### GWAS of albumin-adjusted serum calcium and serum phosphate concentrations in the UK Biobank

Serum albumin–adjusted calcium concentrations for UK Biobank participants were derived using the following equation: adjusted calcium (mmol/L) = total calcium (mmol/L) + 0.0177 × (46.3 – albumin [g/L]). Data from participants with an estimated glomerular filtration rate (eGFR) (Chronic Kidney Disease Epidemiology Collaboration [CKD-EPI]) of less than 60 mL/min/1.73 m^2^ and 25-OH vitamin D concentrations of less than 30 nmol/L were excluded from the association analyses for both serum phosphate concentrations and serum albumin–adjusted calcium concentrations. Primary hyperparathyroidism and hypoparathyroidism are associated with an increased risk of KSD (62, 63). Serum PTH concentrations are not available in the UK Biobank, but nonfasting serum albumin–adjusted calcium and phosphate concentrations are, and we therefore used these as surrogate markers to gauge the possible prevalence of parathyroid disorders in UK Biobank participants with an eGFR above 60 mL/min/1.73 m^2^. This revealed that 0.16% (736 or 458,335) and 10.67% (48,882 of 258,335) of participants had serum calcium concentrations > 2.6 mmol/L and <2.2 mmol/L, respectively. However, these estimates likely include many parathyroid-independent causes of hypercalcemia and hypocalcemia. To refine these estimates, we determined the number of participants with combined hypercalcemia and hypophosphatemia (serum phosphate concentration <0.8 mmol/L) — expected to occur in patients with primary hyperparathyroidism — or with combined hypocalcemia and hyperphosphatemia (serum phosphate concentration >1.5 mmol/L) — expected to occur in patients with hypoparathyroidism. This revealed that only 0.02% (75 of 458,335) and 0.07% (308 of 458,335) of the participants had combinations that may be expected to occur in primary hyperparathyroidism or hypoparathyroidism, respectively. Thus, these data suggest that the prevalence of primary hyperparathyroidism or hypoparathyroidism in the UK Biobank is low and would be unlikely to have a major effect on study results. Thus, individuals with potential parathyroid dysfunction were not excluded from calcium, phosphate, or kidney stone association analyses. We performed analyses using genotyped and imputed variants from the UK Biobank. Genotyping was undertaken using UK-BiLEVE and UK Biobank Axiom arrays. Phenotypes were inverse-normalized with additional adjustments for array, age, and sex. Analyses were undertaken in individuals of European ancestry using BOLT-LMM to account for population substructure and cryptic relatedness. Imputed SNPs with a MAF of less than 1% and an imputation quality score of less than 0.3 were excluded from the analyses. Lead SNPs were identified from unconditional analyses and loci defined as ± 500 kb surrounding each SNP. Overlapping loci were merged as 1 locus. GCTA software was used to perform a stepwise model selection procedure to select independently associated SNPs within each 1 Mbp region with a *P* value significance of less than 5 × 10^–9^. Directly genotyped variants underwent stringent QC checks, including call rate per array, manual cluster plot checks, and status in gnomAD. Only variants with a MAF of less than 1% and the coding or loss-of-function annotations of “missense variant,” “stop gain,” “frameshift variant,” “splice acceptor variant,” “splice donor variant,” “splice region variant,” “start lost,” or “stop lost” were included. A significance threshold *P* value of less than 5 × 10^–6^ was used to identify directly genotyped SNPs that had significant associations with each phenotype.

### MR analyses

MR assumes that IVs are associated with the exposure variable (relevance), that there are no unmeasured confounding relationships (exchangeability), and that variants are associated with the outcome only through changes in the exposure variable (exclusion restriction) (64). Following the recommendation of Gkatzionis et al. (65) that *cis* MR should “contain the target gene, as well as variants within a few hundred thousand base pairs on either side of the gene,” regional (1 Mbp) effects of genetically predicted serum calcium and phosphate concentrations on the odds of KSD were estimated (TwoSample MR package, Rv4.3.1) (66). This was accomplished by selecting independent (*r*^2^ < 0.1) genetic variants ± 500 kbp of lead independent variants from serum albumin–adjusted calcium or phosphate GWASs significantly (*P* < 5 × 10^–8^) associated with biochemical traits for use as IVs (Figure 1). LD between variants was calculated using the “clump_data” function, with a European population as a reference. Given the exploratory nature of these investigations, variants with a LD *r^2^* of less than 0.1 were retained for MR analyses to reduce the risk of type 2 error. Exposure IVs were harmonized with outcome IVs from summary statistics generated by UK Biobank and FinnGen GWASs for KSD and the meta-analysis described above. Allele frequencies were used to infer positive strand alleles for palindromic IVs. Where harmonization was not possible and the positive strand alleles remained ambiguous, IVs were omitted. A power calculation was performed to calculate the minimum and maximum effects that we had 80% statistical power to detect (Supplemental Table 7) (67–69).

Primary MR analyses used the inverse variance–weighted method and individual MR estimates were calculated using the Wald ratio. The MR-Egger intercept and estimate were used to explore pleiotropic relationships, where the MR-Egger intercept was significantly different from zero (*P* < 0.05), the MR-Egger estimate was interpreted as the estimate of best fit. Results are presented as effect estimates and corresponding 95% CIs per SD decrease in mineral metabolism trait on the odds of KSD. Cochrane’s *Q* test was used to identify heterogeneity in causal estimates.

### Colocalization analyses

Once potential causal regional effects of serum calcium or phosphate concentrations on KSD were identified, colocalization analyses [*Coloc ()* and HyPrColoc, Rv4.3.1] were used to evaluate the probability of a single shared causal variant (11, 12) and to identify putative causal variants, considering data from KSD, serum albumin–adjusted calcium, phosphate, and PTH GWASs (Figure 1) (10, 13, 14). *Coloc ()* integrates evidence over all variants at a locus to enable evaluation of several hypotheses (11, 12). H0: The genomic region is associated with neither the KSD nor the mineral metabolite trait; H1: the genomic region is associated with the mineral metabolite trait but not KSD; H2: the genomic region is associated with KSD but not the mineral metabolite trait; H3: the genomic region is associated with the mineral metabolite trait and KSD, with 2 separate putative causal variants; H4: the genomic region is associated with the mineral metabolite trait and KSD, with 1 putative causal variant. coloc.abf () in the colocR package with prior probabilities set to p1 = 1 × 10^–4^, p2 = 10 × 10^–4^, and p12 = 1 × 10^–5^ was used, and the results with a PP H4 of greater than 0.80 were considered to show strong evidence of colocalization (11).

### Drug target MR and phenome-wide association studies

The potential utility of modulating drug targets to prevent KSD was estimated using genetic proxies ± 300 kbp of target genes significantly associated (*P* < 5 × 10^–8^) with relevant mineral metabolism traits (Figure 1) (10). Using smaller genomic regions defined by target-gene coordinates, rather than 1 Mbp loci used during regional MR, provided enhanced estimates of the clinical utility of target modulation (Supplemental Figure 1). Analyses were conducted using UK Biobank, FinnGen R12, and meta-analyzed KSD GWAS data. MR analyses were performed using the principles described above. Sensitivity analyses were undertaken using the “clump_data” function with LD *r^2^* thresholds of 0.1 and 0.01. Possible off-target effects of putative therapeutics were identified by collating phenotypes associated with variants linked to these genes (*P* < 5 × 10^–8^) via the Open Targets Genetics portal (https://genetics.opentargets.org/, accessed 30/01/2024) (70, 71).

### Associations in the DiscovEHR cohort

Genotype in the DiscovEHR cohort was determined using DNA extracted from MyCode participant blood or saliva samples collected as part of the DiscovEHR collaboration (72). Regeneron Genetic Center (RGC) (Tarrytown, New York, USA) performed genotyping using the Illumina Infinium Global Screening Array GSA-24v2-0_A2 (Illumina), and then filtered for a MAF of greater than 1%, a Hardy-Weinberg equilibrium (HWE) *P* value of greater than 1 × 10^–15^, and a site missingness of less than 1%. Data were uploaded by batch to the TOPMed Imputation Server for genotype imputation using MINIMAC4 and the TOPMed reference panel (73).

Individuals with a history of kidney stones were identified in the DiscovEHR cohort on the basis of the presence of kidney stone ICD-10 codes (N20.0, N20.1, N20.2, N20.9 or N23) in their electronic health record. Exome data were filtered using the following QC metrics: a MAF of less than 0.1, a combined depth of 10 or more for indels, a quality by depth of more than 3, a combined depth of 7 or more for single nucleotide variants, an alternate allelic balance of greater than 15% (single nucleotide variants) or greater than 20% (indels), and 5 or more alternate reads. Variants were annotated with variant type (e.g., missense), human genome variation, and variant definition (including position, gene name, predicted protein-coding alterations). The frequency of rare *DGKD* missense variant carriers with and without a kidney stone diagnosis was compared using the *χ*^2^ test or Fisher’s exact test (as appropriate). Unadjusted *P* values and ORs with 95% CIs were calculated using the *χ*^2^ test and the Cochran-Mantel-Haenszel statistic, respectively, to compare the frequency of a kidney stone diagnosis between individuals carrying a variant of interest and those not carrying a variant of interest. Analysis was performed using SAS Enterprise Guide, version 8.3 (SAS Institute).

Outpatient serum calcium and serum phosphorus laboratory values were collected and used to determine the median value. The average median value for serum calcium and serum phosphate in carriers and noncarriers was then compared. The log-transformed serum phosphorus values were used for all statistical analyses, as serum phosphorus values were not normally distributed. Effect sizes and *P* values, adjusted for kidney stone diagnosis, were generated using logistic regression to assess how serum calcium and serum phosphorus in carriers are affected with the addition of 1 putative kidney stone–causing allele. This analysis was performed for each individual putative kidney stone–causing variant and with all 3 putative kidney stone–causing variants combined. Analysis was performed using SAS Enterprise Guide, version 8.3.

### Relationship and pedigrees in the DiscovEHR cohort

To determine the relatedness between individuals within the DiscovEHR cohort, genome-wide identity by descent (IBD) was used (74). Ancestral class (admixed American, African, East Asian, European, South Asian, and unknown) was determined using principal components and the HapMap3 dataset. High-quality common variants (MAF >0.1, missingness 0.05, and expected heterozygosity rates) and high-quality samples (percentage ×20 coverage <0.75) were used to calculate pairwise IBD values within ancestral groups. A PI_HAT threshold 0.1875 was set for second-degree relationships and 0.3 for first-degree relationships Individuals were then grouped into family networks and run through PRIMUS (75) for improved IBD estimates to determine the relationships within each family network.

### Functional and structural characterization of DGKδ variants

Individuals with KSD and a predicted “deleterious” (SIFT) and “probably damaging” (PolyPhen) *DGKD* missense variant with a MAF of less than 0.1% were identified in Genomics England 100KGP (Integrative Variant Analysis 2.0). Clinical data were obtained from Patient Explorer and referring clinicians. Conservation of DGKδ variants was assessed by aligning DGKδ orthologs with Clustal Omega.

Functional studies were conducted using HEK293 cells (ATCC CRL-1573, ThermoFisher Scientific) that were transfected with the FLP-In system to express CaSRs (HEK-FLP-In CaSR cells). HEK293 cells were chosen, since suitable parathyroid and renal thick ascending limb cells are not available and because HEK293 cells are an established model for assessment of CaSR signal transduction (76, 77). For overexpression studies, a Myc-DDK–tagged DGKDv2 cDNA (NM_152879) in the pCMV6_Entry vector clone was purchased from Origene (catalog RC217053). Point mutations were introduced into this clone using the QuickChange Lightning Site Directed Mutagenesis kit (Agilent Technologies) according to the manufacturer’s instructions to produce constructs containing the I91V, H190Q, I221N, T319A, V464I, R900H, and R1181W variants. Constructs were sequenced to confirm the presence of variants prior to transfection into HEK FLP-In CaSR cells and OE stable cell lines selected for by growth in Geneticin media. Cell lines were subsequently maintained in DMEM-GlutaMAX media (Thermo Fisher Scientific) with 10% FBS (Gibco, Thermo Fisher Scientific), 400 μg/mL Geneticin (Thermo Fisher Scientific), and 200 μg/mL hygromycin (Invitrogen, Thermo Fisher Scientific) at 37°C, 5% CO_2_. These cell lines were utilized in subsequent SRE and nuclear factor of activated T cells (NFAT) assays.

Expression of DGKδ and the CaSR was confirmed by Western blotting. Western blot analyses were performed using anti-cMyc (A190-105P; Thermo Fisher Scientific; 1:3,000), anti-DGKδ (GTX87254; GeneTex; 1:1,000), anti-CaSR (5C10, ADD; ab19347; Abcam; 1: 6,000), and anti−α-tubulin (T5168; MilliporeSigma; 1:3,000) antibodies. The Western blots were visualized using an Immuno-Star Western C kit (Bio-Rad) on a Bio-Rad Chemidoc XRS+ system.

A *DGKD* Human shRNA Plasmid kit (locus ID8527) (Origene, catalog TF313492) containing 4 differing shRNAs in the pRFP-C-RS vector was utilized to generate stable KD cell lines in HEK-FLP-In CaSR cells according to the manufacturer’s instructions. Stable cell lines were maintained in DMEM-GlutaMAX media (Thermo Fisher Scientific) with 10% FBS (Gibco, Thermo Fisher Scientific), 1 μg/mL puromycin (Thermo Fisher Scientific), and 200 μg/mL hygromycin (Invitrogen, Thermo Fisher Scientific) at 37°C, 5% CO_2_. These cell lines were used in subsequent SRE and NFAT assays.

Successful KD of DGKD and maintenance of CaSR expression were confirmed via quantitative reverse transcriptase PCR (qRT-PCR) and Western blot analyses. qRT-PCR analyses were performed in quadruplicate using the Power SYBR Green Cells-to-CT Kit (Life Technologies, Thermo Fisher Scientific), *DGKD*-, *CASR*-, *PGK1*-, *GAPDH*-, *TUB1A*-, and *CDNK1B*-specific primers (Qiagen), and a Rotor-Gene Q real-time cycler (Qiagen). Samples were normalized to a geometric mean of 4 housekeeper genes: *PGK1*, *GAPDH*, *TUB1A*, and *CDNK1B*.

To perform pERK response assays, HEK-FLP-In CaSR-DGKδ–OE or –KD cells were placed in 96-well plates. Twenty-four hours after seeding, cells were incubated in serum-free DMEM containing 0.1 mM calcium and incubated for 4 hours, the media were then changed to varying concentrations of extracellular calcium (0.1–5 mM) for 2.5 minutes, and the cells were lysed in lysis buffer. ERK1/2 (phosphorylated at Thr202/Tyr204), total ERK 1/2, and GAPDH were measured using AlphaLISA SureFire Ultra assay kits (ALSU-PERK-A500, ALSU-TERK-A500, ALSU-TGAPDH-A500; Revvity) according to the manufacturer’s instructions. pERK was normalized to total ERK and GAPDH. Data represent the average of 2 technical replicates from 8 separate experiments.

To perform SRE assays, HEK-FLP-In CaSR-DGKδ–OE or –KD cells were placed in 96-well plates and transfected with an SRE reporter assay plasmid (Promega) using Lipofectamine 2000 (Invitrogen, Thermo Fisher Scientific) according to the manufacturer’s instructions. Thirty-six hours after transfection, cells were incubated in 0.05% FBS media with 0.45 mM calcium for 12 hours, reducing the extracellular calcium concentration and thus inducing basal cellular CaSR-mediated responses while maintaining cellular viability. Forty-eight hours after transfection, the media were changed to varying concentrations of extracellular calcium (0.1–5 mM), with either 5 nM cinacalcet, 100 nM cinacalcet, or an equivalent volume of DMSO (final concentration of DMSO: 0.0001%), and the cells were incubated for a further 4 hours at 37°C. Cinacalcet (AMG-073 HCL) was obtained from Cambridge Bioscience (catalog CAY16042) and dissolved in DMSO prior to use in the in vitro studies. Cells were lysed and luciferase activity measured using the Luciferase Assay System (Promega) on a PHERAstar microplate reader (BMG Labtech). Assays were performed in more than 4 biological replicates (independently transfected wells, performed on at least 4 different days).

To perform the NFAT response assays, HEK-FLP-In CaSR-DGKδ–OE or –KD cells were placed in 96-well plates and transfected with an NFAT reporter assay plasmid (Promega) using Lipofectamine 2000 according to the manufacturer’s instructions. Thirty-six hours after transfection, cells were incubated in 0.05% FBS media with 0.45 mM calcium for 12 hours, reducing the extracellular calcium concentration and thus inducing the basal cellular CaSR-mediated responses, while maintaining cellular viability. Forty-eight hours after transfection, the media were changed to varying concentrations of extracellular calcium (0.1–10 mM), with either 5 nM cinacalcet, 100 nM cinacalcet, or an equivalent volume of DMSO (final concentration of DMSO: 0.0001%), and the cells were incubated for a further 4 hours at 37°C. Cells were lysed and luciferase activity measured using the Luciferase Assay System (Promega) on a PHERAstar microplate reader (BMG Labtech). Assays were performed in more than 4 biological replicates (independently transfected wells, performed on at least 4 different days).

Cellular responses to increasing extracellular calcium concentrations were compared using 2-way ANOVA with Dunnett’s multiple-comparisons test (GraphPad Prism, version 9).

### 3D modeling of the DGKδ structure

The crystal structure of oligomeric DGKδ SAM domains has been determined (PDB 3BQ7), and the structure of DGKδ isoform 2 has been predicted (AF-Q16760-F1-mod; AlphaFold; refs. 78, 79). The PyMOL Molecular Graphics System, version 2.5.2 (Schrödinger) was used for structural modeling based on these structures (80, 81). The PyMOL Molecular Graphics System, version 2.5.2, and PyMod, version 3.0, were used to model the effects of DGKδ variants (80, 82, 83).

### Statistics

#### GWASs and meta-analysis.

GWASs were undertaken using a linear mixed noninfinitesimal model, and significant associations were considered for variants with a *P* value of less than 5 × 10^–8^. Conditional analyses were undertaken iteratively at loci with multiple association signals using Genome-Wide Complex Trait Analysis software, version 1.94 (53, 54).

A fixed-effects meta-analysis of KSD GWASs was performed, and significant associations were considered for variants with a heterogeneity I^2^ of 75% or less and a *P* value of less than 5 × 10^–8^. For associations of KSD with genes and gene sets, a Bonferroni-adjusted *P* value of less than 0.05/number of genes or number of gene sets tested was interpreted as evidence against the null hypothesis.

#### MR.

For MR analyses, *P* values were corrected for multiple testing using the Benjamini-Hochberg FDR method, controlled at 5%, and a corrected *P* value of less than 0.05 was considered significant (49). The variance in exposure trait explained by the IVs in each MR analysis was calculated as (*r^2^* = [2 × MAF × (1 – MAF) × β^2^], where MAF is the MAF, and β is the log-odds of the SNP) (84). The mean *F* statistic for exposure IVs was calculated as follows:


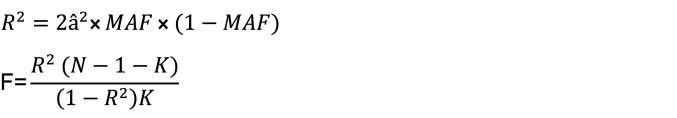
(Equations 1 and 2)

The genetic association with the risk factor (â) is provided in SD units, the MAF is given, *N* is the sample size for the IV outcome association, and *K* is the number of IVs (85). Cochrane’s *Q* test was used to assess heterogeneity in causal estimates.

#### Colocalization analyses.

Colocalization analyses were performed with prior probabilities set at p1 = 1 × 10^–4^, p2 = 10 × 10^–4^, and p12 = 1 × 10^–5^. Where there was evidence of colocalization between albumin-adjusted serum calcium concentrations or phosphate concentrations and KSD using *Coloc* (posterior probability [PP] ≥0.80)*,* multi-trait colocalization analyses were performed with *HyPrColoc* to include albumin-adjusted serum calcium, phosphate, and PTH concentrations and KSD studies simultaneously. Default prior probabilities were set as prior.1 = 1 × 10^–5^, prior.12 = 1 × 10^–5^. Again, a PP of 0.80 or higher was interpreted as strong evidence of colocalization in HyPrColoc analyses.

#### Phenome-wide association studies.

The Open Targets Genetics portal was used to identify possible off-target effects of candidate therapeutics (70, 71). Phenotypes reported for variants significantly (*P* < 5 × 10^–8^) associated with genes of interest were identified.

### Genotype-phenotype associations in the DiscovEHR cohort

Genotyped variants were filtered for a MAF of greater than 1%, a Hardy-Weinberg equilibrium *P* value of greater than 1 × 10^–15^, and site missingness of less than 1%. Imputation was carried out using a TOPMed reference panel. Statistical analyses were performed using SAS Enterprise Guide, version 8.3 (SAS Institute). ORs and 95% CIs to compare the frequency of a kidney stone diagnosis between variant carriers and noncarriers were determined using χ^2^ tests and Cochran-Mantel-Haenszel statistics. Serum calcium and phosphate concentrations were normalized using log transformation, and the effect of adding 1 variant allele on serum calcium and phosphate concentrations was assessed using logistic regression (adjusted for case/control status) Data were plotted as mean ± standard error (SEM). We also assessed the effect of combining all variants.

### Functional characterization of DGKδ mutations and reduced DGKδ expression

Responses of HEK293 cells to increasing extracellular calcium concentrations were assessed via pERK, SRE, and NFAT response assays. Assays were performed in more than 4 biological replicates and compared by 2-way ANOVA with Dunnett’s multiple-comparison test using GraphPad Prism version 9 (GraphPad Software). Significant differences were defined as a *P* value of less than 0.05 Data were plotted as mean ± standard error (SEM).

### Study approval

The UK Biobank received approval from the North West Multi-Centre Research Ethics Committee (11/NW/0382). Ethics approval for the 100KGP was granted by the Cambridge South Research Ethics Committee for the East of England (REC Ref14/EE/1112; Cambridge, United Kingdom). Additional informed consent was obtained from participants in 100KGP using protocols approved by the London Central Research Ethics Committee (MREC/02/2/93, London, United Kingdom). Participants from the DiscovEHR cohort provided written informed consent for participation in the MyCode Community Health Initiative, an Institutional Review Board-approved project (Geisinger IRB, Danville, Pennsylvania, USA, protocol 2006-0258) that allows for genetic analysis and linking to information from the electronic health records. The research included in this publication was reviewed and determined to be exempt by the Geisinger IRB no. 2023-1786.

### Data availability

Individual participant data utilized in the preparation of this manuscript are available via the UK Biobank. Underlying data values are presented in the Supporting Data Values file. Exposure IVs for serum albumin–adjusted calcium and phosphate concentrations, results from MR and colocalization analyses of serum albumin–adjusted calcium and phosphate concentrations with kidney stones in the UK Biobank and FinnGen cohorts, and full results of the phenome-wide association studies are available via Figshare (9) (https://doi.org/10.25446/oxford.26968825.v2).

## Author contributions

CEL, MG MVH, DF, RVT, and SAH were responsible for study conception. CEL, MG, JH, DS, FMH, AM, MS, OSA, SM, DPG, DC, SAH acquired data. CEL MG, JH, DS, CG, DC, MVH, DF, RVT, and SAH analyzed and interpreted data. CEL MG, JH, DS, CG, FMH, MS, OSA, SM, DPG, DC, MV, DF, RVT, and SAH prepared and reviewed the manuscript. CEL, MG, JH, DS, CG FMH, AM, MS, OSA, SM, DPG, DC, MVH, DF, RVT, and SAH were responsible for final approval of the manuscript.

## Supplementary Material

Supplemental data

ICMJE disclosure forms

Unedited blot and gel images

Supporting data values

## Version 1 05/15/2025

In-Press Preview

## Version 2 08/01/2025

Electronic publication
