# Supplementary material for: Genetic variants predisposing to an increased risk of kidney stone disease
Source: J Clin Invest. 2025 May 15;135(15):e186915. doi: 10.1172/JCI186915 (PMC12321396; doi:10.1172/JCI186915)
Supplement: Supplemental data [file jci-135-186915-s121.pdf]

Supplementary Appendix

Supplement to: Lovegrove CE, Goldsworthy M, Haley J, et al. Genetic variants predisposing to increased risk of kidney stone disease

This appendix has been provided by the authors to give readers additional information about the work.

Contents

Supplementary Figure S1: Quantile-quantile plot of observed vs. expected p-values for genome-wide association study (GWAS) of 11,186 kidney stone disease cases and 390,488 controls in the UK Biobank. 4

Supplementary Figure S2: Manhattan plot for genome-wide association study (GWAS) of 11,186 kidney stone disease cases and 390,488 controls in the UK Biobank. 5

Supplementary Figure S3: Quantile-quantile plot of observed vs. expected p-values for a kidney stone disease genome-wide association study meta-analysis in the UK Biobank and FinnGen R12 Study. 6

Supplementary Figure S4: Locus zoom plots showing genetic coordinates from which regional and drug-target Mendelian randomization instrumental variables were selected. 7

Supplementary Figure S5: Forest plot demonstrating regional Mendelian randomization estimates for independent serum calcium and phosphate index-associated variants (±500kb) on liability to kidney stone disease in a meta-analysis of the UK Biobank and FinnGenR12 study and each constituent study. 9

Supplementary Figure S6: Multiple protein sequence alignment of DGKδ 10

Supplementary Figure S7: Proposed role of diacylglycerol kinase delta (DGKD) in the calcium sensing receptor signaling pathway. 11

Supplementary Figure S8: Expression of DGKδ variants in HEK293 cells. 12

Supplementary Figure S9: Functional characterization of kidney stone-associated DGKδ variants. 15

Supplementary Figure S10: Predicted effects of kidney stone-associated DGKδ variants based on the predicted structure of DGKδ isoform 2, (AF-Q16760-F1-mod(2, 3) AlphaFold) 16

Supplementary Table S1: Inclusion criteria for kidney stone disease association analyses in the UK Biobank. 18

Supplementary Table S2: Variants associated with kidney stone disease at genome-wide association study in the UK Biobank. 24

Supplementary Table S3: Heritability estimates for GWAS in UK Biobank and in UK Biobank-FinngenR12 GWAS meta-analysis. 25

Supplementary Table S4: Variants associated with kidney stone disease from meta-analysis of genome-wide association studies in the UK Biobank and FinnGenR12 study. 26

Supplementary Table S5: MAGMA gene association test for meta-analysis of genome-wide association studies in the UK Biobank and FinnGenR12 study. 30

Supplementary Table S6: MAGMA Gene-set analysis in meta-analysis of genome-wide association studies in the UK Biobank and FinnGenR12 study. 32

Supplementary Table S7: Estimate of bias for Mendelian randomization with sample overlap in the UK Biobank. 33

Supplementary Table S8: Causal effects of lead variants from mineral metabolism GWAS (±500kb) with evidence of colocalization on kidney stone risk. 34

Supplementary Table S9: 95% credible sets for genomic regions with evidence for causal effects on kidney stone risk and colocalization in UK Biobank-FinnGenR12 genome-wide association study meta-analysis...38

Supplementary Table S10: 95% credible sets for genomic regions with evidence for causal effects on kidney stone risk and colocalization in the UK Biobank...................................41

Supplementary Table S11: 95% credible sets for genomic regions with evidence for causal effects on kidney stone risk and colocalization in the FinnGenR12 study. 42

Supplementary Table S12: Variants predicted to increase risk of kidney stones via effects on serum mineral metabolism...44

Supplementary Table S13: Associations of variants predicted to cause kidney stones with kidney stone disease and mineral metabolism traits. 46

Supplementary Table S14: Associations of variants predicted to cause kidney stones with kidney stone disease and mineral metabolism traits in DiscovEHR. 47

Supplementary Table S15: Kidney stone population attributable risk and population attributable fraction in the UK Biobank....48

Supplementary Table S16: Kidney stone population attributable risk and population attributable fraction in DiscovEHR...49

Supplementary Table S17. Drug target Mendelian randomization; effects of modulating mineral metabolism traits on odds of kidney stone disease...50

Supplementary Table S18: Rare predicted deleterious DGKD missense variants in 100kGP participants with kidney stone disease....52

Supplementary Table S19: Rare DGKD missense variants associated with kidney stone disease in the DiscovEHR cohort....53

Bibliography 54

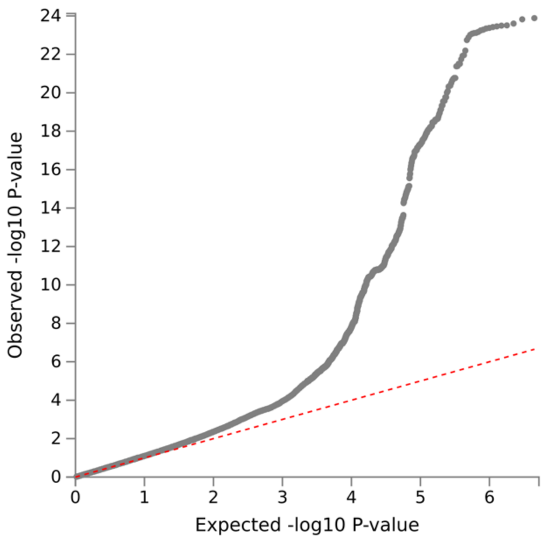

Supplementary Figure S1: Quantile-quantile plot of observed vs. expected p-values for genome-wide association study (GWAS) of 11,186 kidney stone disease cases and 390,488 controls in the UK Biobank.

The λGC demonstrated some inflation (1.15), but the LD score regression (LDSC) intercept of 1.01, with an attenuation ratio of 0.09 indicated that the inflation was largely due to polygenicity and the large sample size.

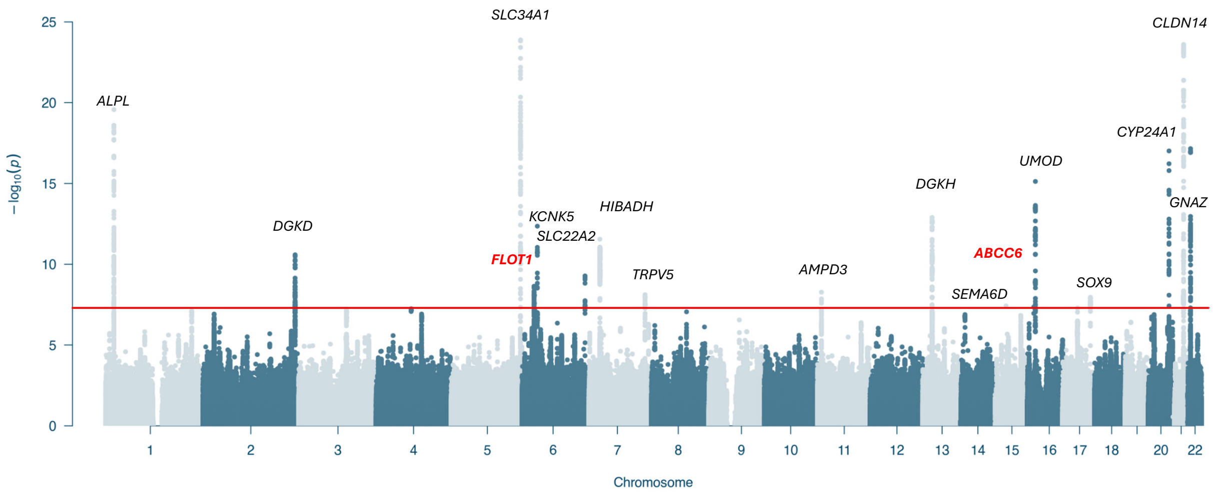

Supplementary Figure S2: Manhattan plot for genome-wide association study (GWAS) of 11,186 kidney stone disease cases and 390,488 controls in the UK Biobank.

Genome-wide p-values (-log10) are plotted against their respective positions on each chromosome. The horizontal red line indicates the genome-wide significance threshold (5.0 x 10−8). Loci are labelled with the primary candidate gene at each locus. These loci are in proximity to biomineralization associated alkaline phosphatase, ALPL; diacylglycerol kinase delta, *DGKD*; solute carrier family 34 member 1, *SLC34A1*; flotillin 1, *FLOT1*; potassium two pore domain channel subfamily K member 5, *KCNK5*; solute carrier family 22 member 2, *SLC22A2*; 3-hydroxyisobutyrate dehydrogenase, *HIBADH*; transient receptor potential cation channel subfamily V member 5, *TRPV5*; adenosine monophosphate deaminase 3, AMPD3; diacylglycerol kinase eta, *DGKH*; semaphoring 6D, *SEMA6D*; ATP binding cassette subfamily C member 6, *ABCC6*; uromodulin, *UMOD*; SRY-box transcription factor, *SOX9*; cytochrome P450 family 24 subfamily A member 1, *CYP24A1*; claudin 14, *CLDN14*; and G protein subunit alpha z, *GNAZ*. Two loci (*FLOT1* and *ABCC6*) have not been previously associated with kidney stone disease.

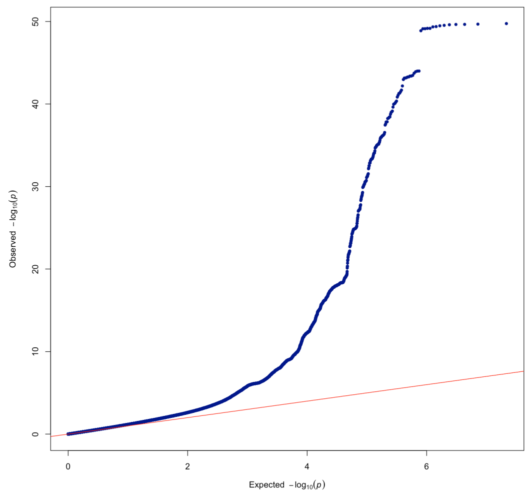

Supplementary Figure S3: Quantile-quantile plot of observed vs. expected p-values for a kidney stone disease genome-wide association study meta-analysis in the UK Biobank and FinnGen R12 Study.

The meta-analysis of genome-wide association studies of kidney stone disease comprises 24,167 cases and 876,673 controls from the UK Biobank and FinnGen R12 study. λGC demonstrated some inflation (1.26). The LD score regression (LDSC) intercept of 1.05, with an attenuation ratio of 0.13 indicated that inflation was largely due to polygenicity and large sample size.

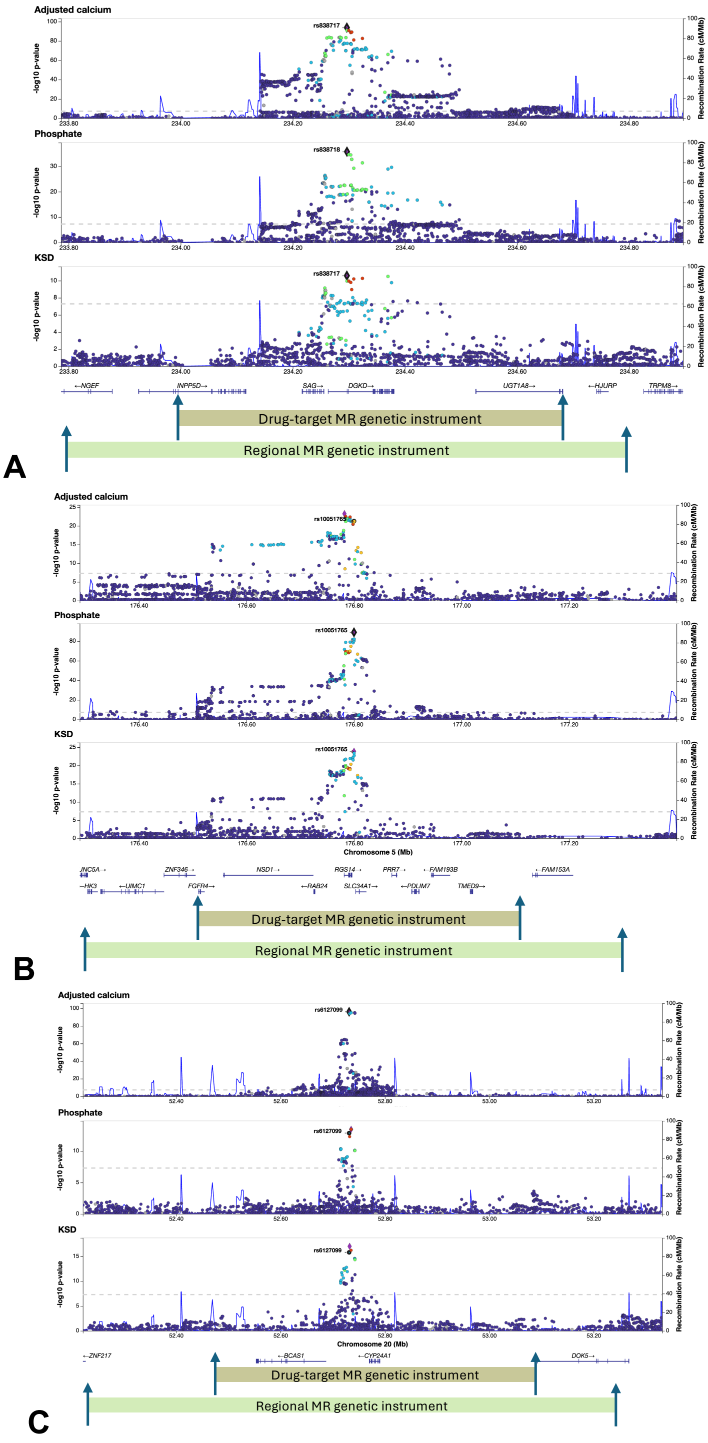

Supplementary Figure S4: Locus zoom plots showing genetic coordinates from which regional and drug-target Mendelian randomization instrumental variables were selected

Green boxes denote genomic regions from which genetic variants were selected for regional Mendelian randomization (MR); these variants were within a 500kbp window either side of

lead independent variants from serum albumin-adjusted calcium or phosphate genome-wide association studies. Brown boxes denote genomic regions from which genetic variants were selected for drug-target MR; these variants were within a 300kbp window either side of the gene coordinates for genes associated with candidate causal variants identified from MR and colocalization analyses. Candidate causal variants are highlighted in each locus zoom plot in the **A**: *DGKD*, **B**: *SLC34A1*, and **C**: *CYP24A1* regions using data from genome-wide association studies of albumin-adjusted serum calcium, phosphate, and kidney stone disease (KSD) in the UK Biobank.

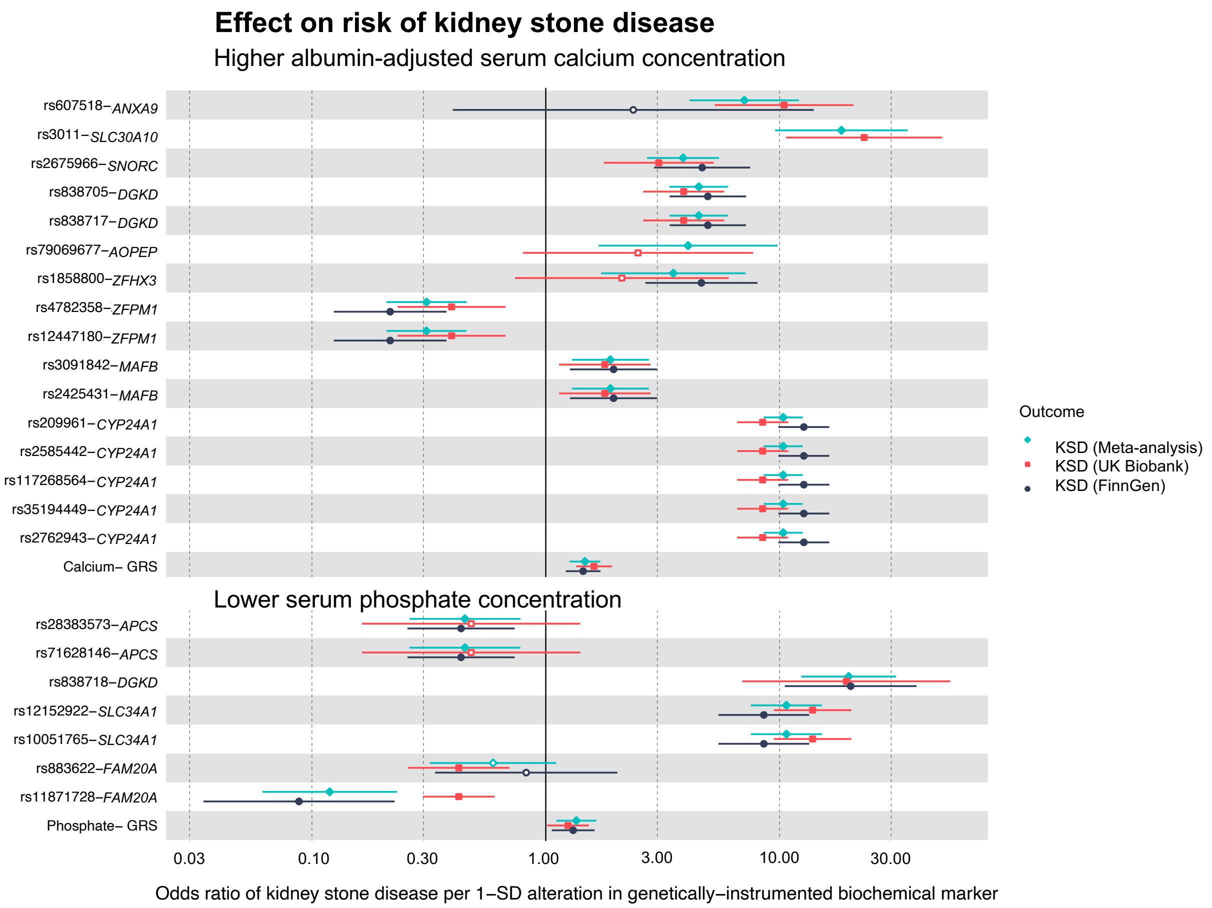

Supplementary Figure S5: Forest plot demonstrating regional Mendelian randomization estimates for independent serum calcium and phosphate index-associated variants (±500kb) on liability to kidney stone disease in a meta-analysis of the UK Biobank and FinnGenR12 study and each constituent study.

GRS=overall Mendelian randomization estimate for trait on risk of kidney stone disease.

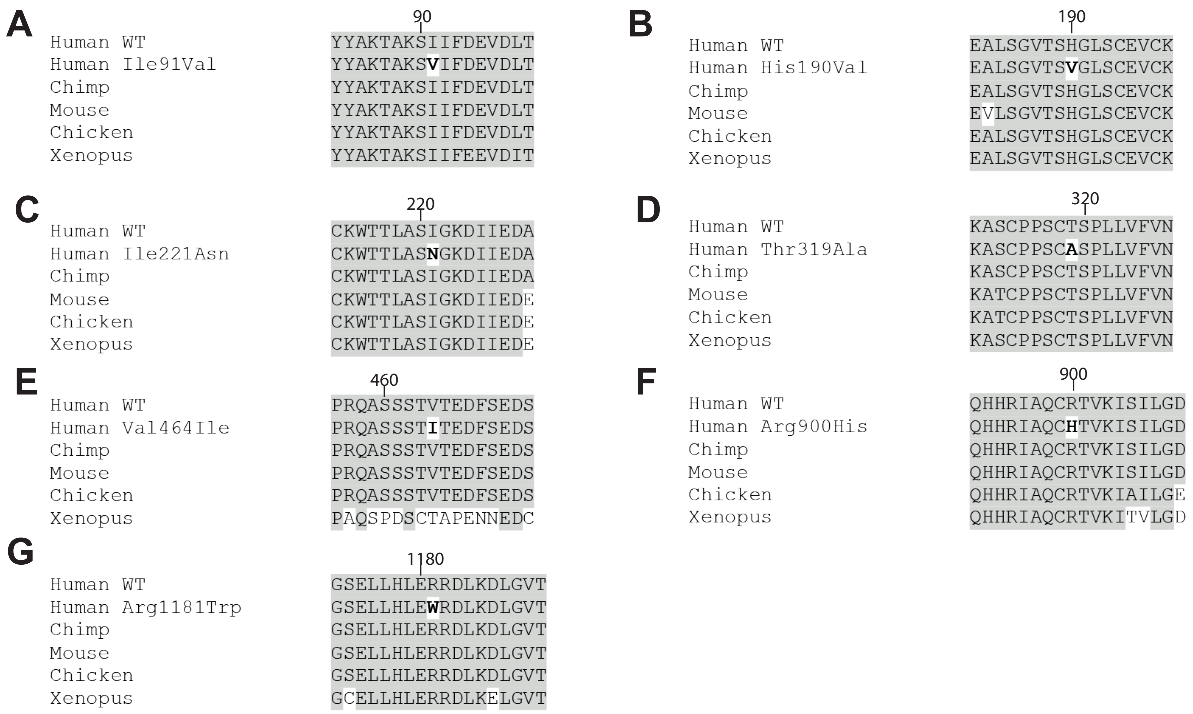

Supplementary Figure S6: Multiple protein sequence alignment of DGKδ

Evolutionary conservation of residues implicated in kidney stone disease in orthologs. Conserved residues are shaded grey.

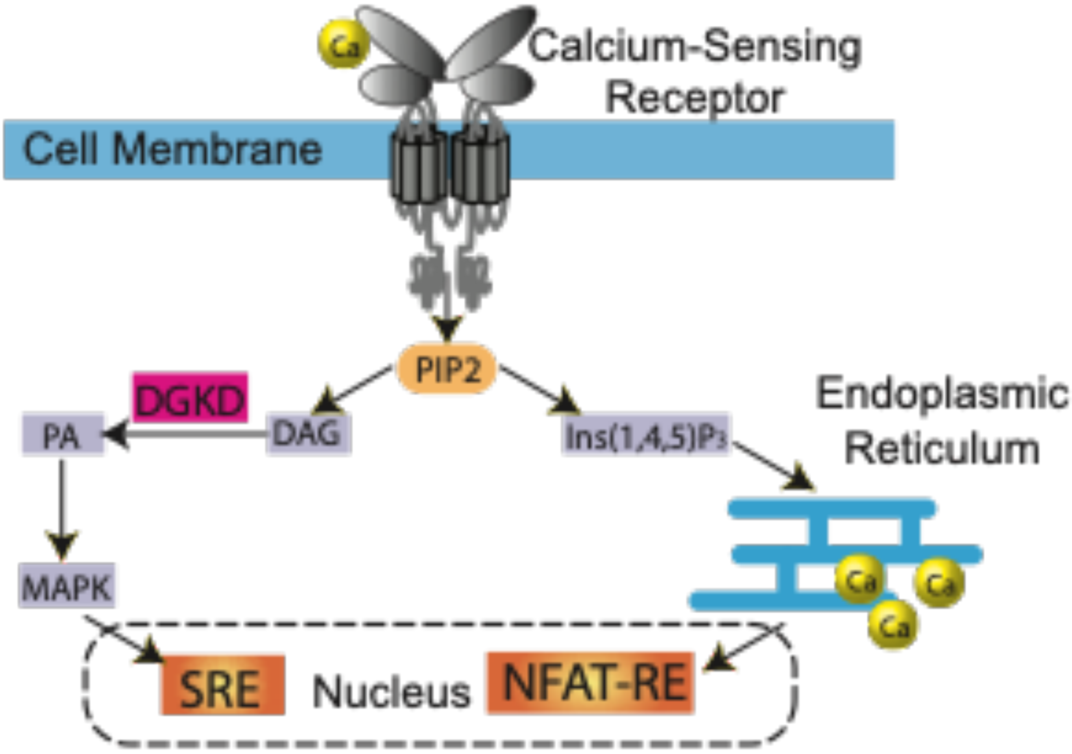

Supplementary Figure S7: Proposed role of diacylglycerol kinase delta (DGKD) in the calcium sensing receptor signaling pathway.

Binding of calcium (yellow) to the extracellular bilobed venus fly-trap domain of the CaSR (light grey) results in Gα11 dependent stimulation of phospholipase C-β, which catalyzes the formation of inositol 1,4,5-trisphosphate (Ins(1,4,5)P_3) and diacylglycerol (DAG) from phosphatidylinositol 4,5-bisphosphate (PIP2). An accumulation of Ins(1,4,5)P_3 mediates calcium mobilization into the cytosol from intracellular stores, whereas DAG activates the phospho-extracellular signal regulated kinase (pERK) arm of the mitogen activated protein kinase (MAPK) cascade via the production of phosphatidic acid (PA). Gene transcription mediated by intracellular calcium and MAPK signaling can be measured using nuclear factor of activated T-cells response element (NFAT-RE) and serum response element (SRE) containing luciferase reporter constructs, respectively(1).

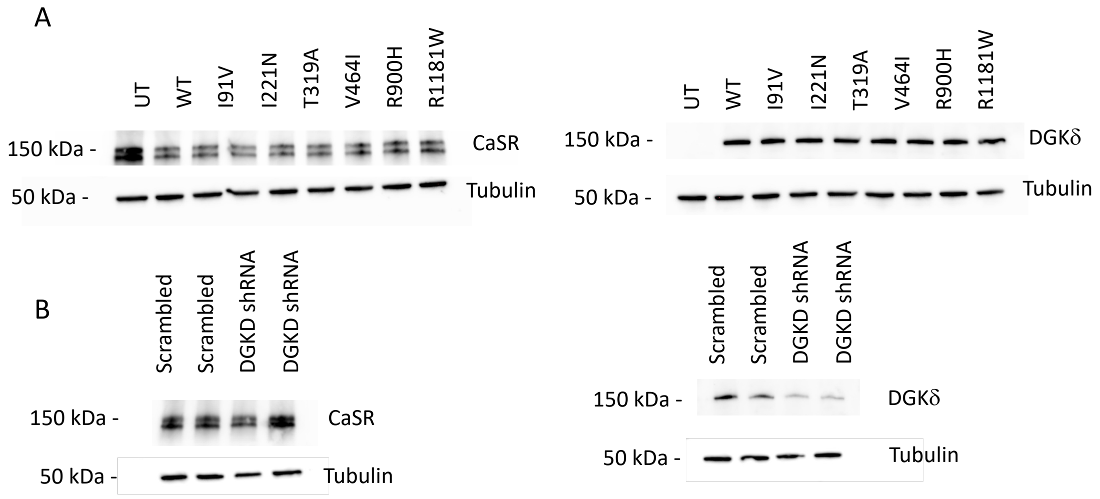

Supplementary Figure S8: Expression of DGKδ variants in HEK293 cells.

A : Representative western blot of lysates from HEK-CaSR stably transfected with Myc-tagged DGKD; α−Tubulin was used as a loading control. Anti-CaSR, anti-tubulin, and anti-Myc antibodies were used. UT-untransfected cells, WT- wildtype. **B:** Representative western blot of lysates from HEK-CaSR cells treated with scrambled or DGKD shRNA; α−Tubulin was used as a loading control. Anti-CaSR, anti-tubulin, and anti-DGKD antibodies were used.

I91V

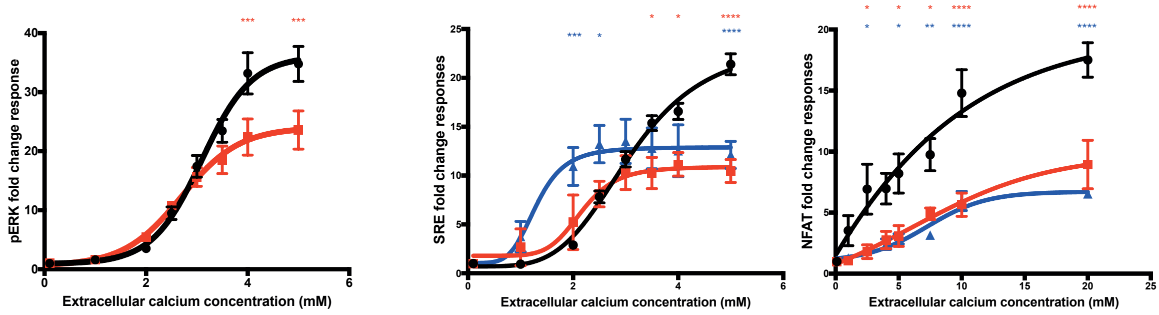

H190Q

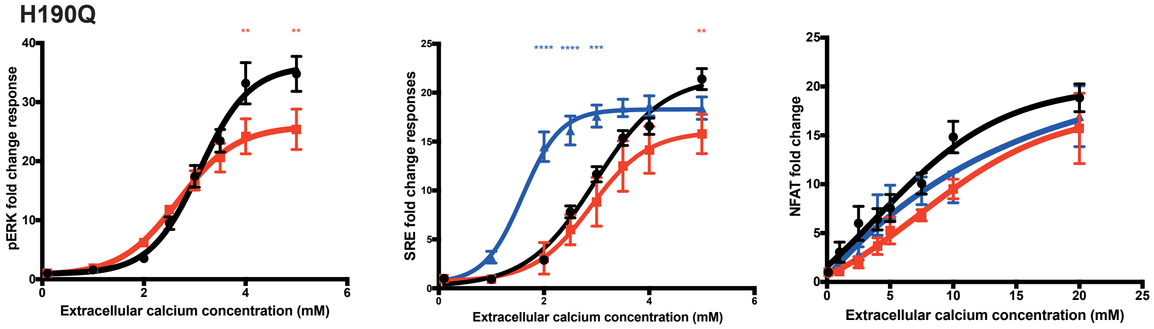

I221N

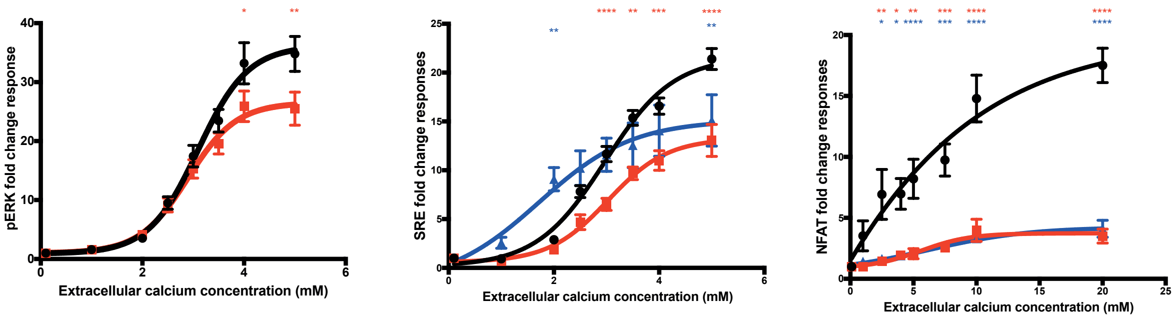

T319A

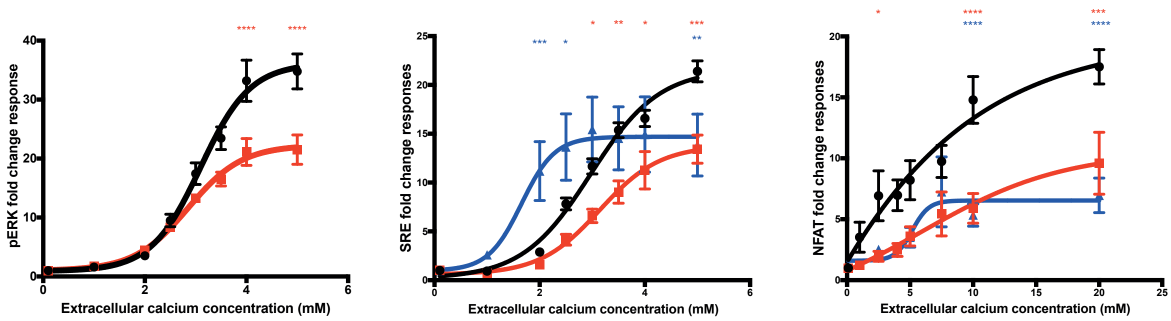

V464I

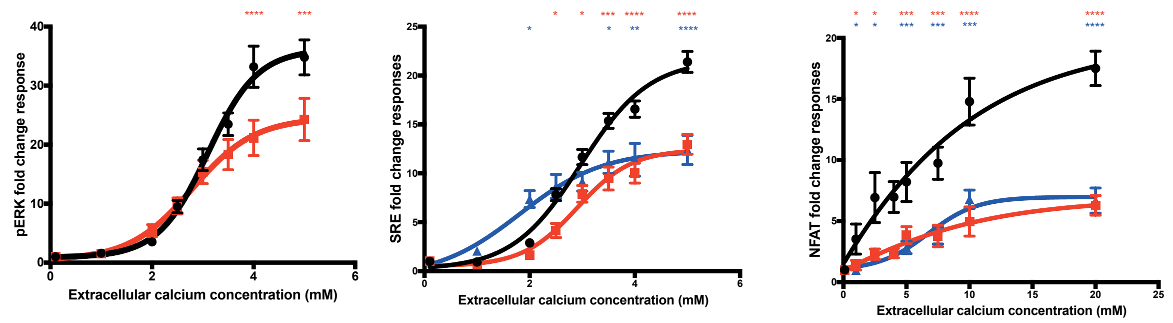

R900H

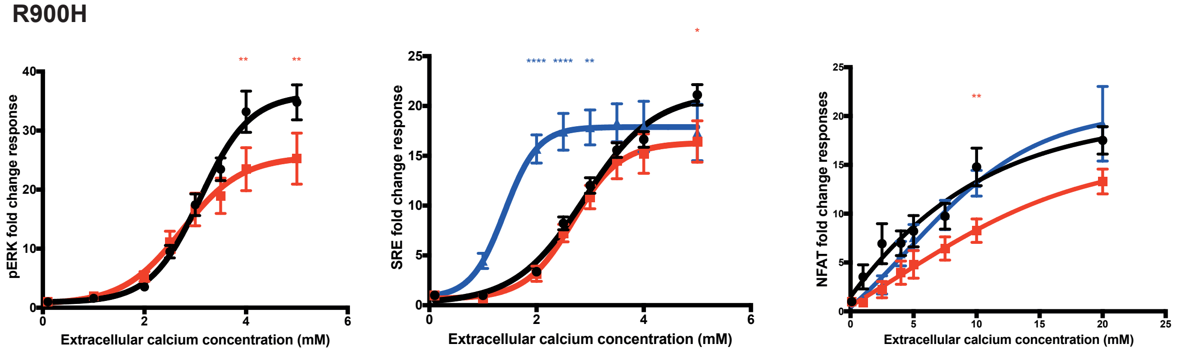

R1181W

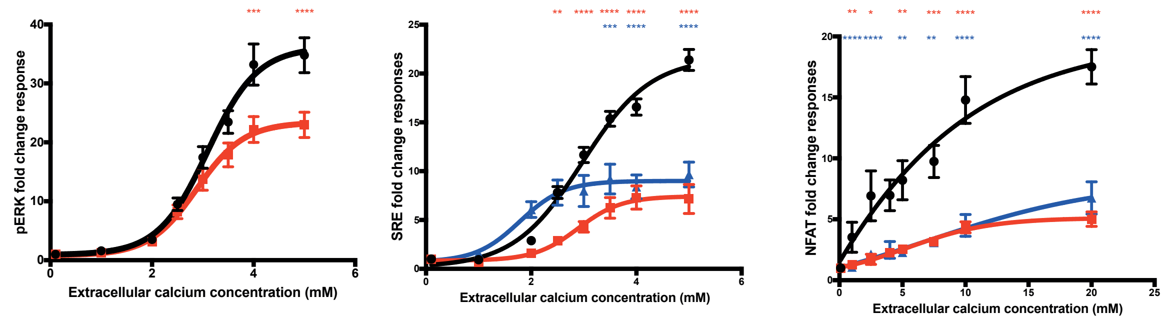

DGKδ knockdown

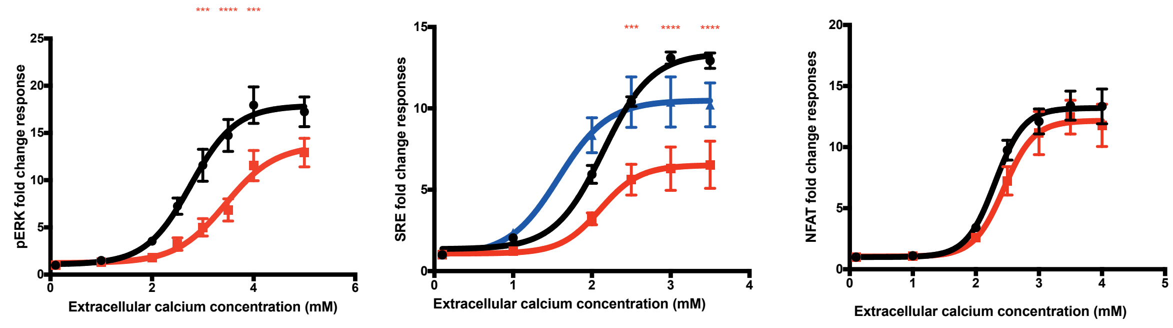

Supplementary Figure S9: Functional characterization of kidney stone-associated DGKδ variants. CaSR-mediated phospho-ERK (pERK), SRE, and NFAT responses to changes in extracellular calcium concentration and effect of 100nM cinacalcet treatment (missense variants) and 5nM cinacalcet (DGKδ knockdown) in HEK-CaSR-DGKδ cells transfected with wild-type or kidney stone-associated variants I91V, H190Q, I221N, T319A, V464I, R900H, and R 1181W, and in HEK-CaSR cells following DGKδ knockdown. The responses ± standard error of the mean (SEM) are shown for n>4 biologically independent experiments. Transfection with kidney stone-associated DGKD variants and DGKδ knockdown led to a reduction in responses (red line) compared to cells transfected with wild-type DGKD (black line). Treatment with cinacalcet increased SRE-mediated responses but had no effect on NFAT responses (blue line). Two-way ANOVA with Dunnet's correction for multiple comparisons was used to compare points on dose response curve with reference to wild-type. Data are shown as mean ± SEM with *p<0.05, **p < 0.01, ***p<0.001, ****p < 0.0001.

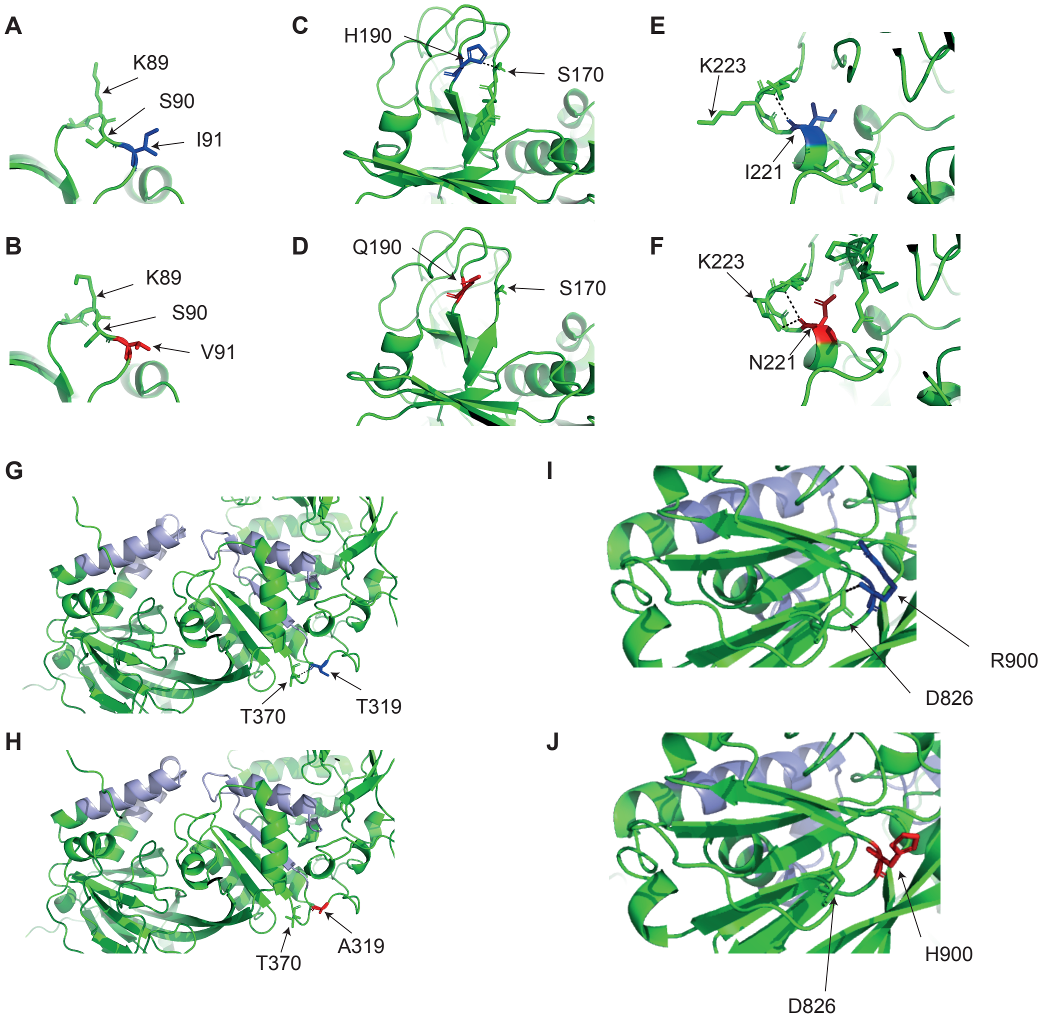

Supplementary Figure S10: Predicted effects of kidney stone-associated DGKδ variants based on the predicted structure of DGKδ isoform 2, (AF-Q16760-F1-mod(2, 3) AlphaFold)

A and B: Residue I91 (wild-type, dark blue) is within the β3-β4 linker of the DGKδ predicted pleckstrin homology (PH) domain; the mutation V91 (red); is predicted to result in an altered confirmation of residues K89 and S90. Residues in an equivalent position have been shown to be important for binding of the PH domain of phospholipase C beta to inositol trisphosphate (4). **C and D:** H190 wild-type, (dark blue) and Q190 (red); the mutation Q190 is predicted to result in the loss of a polar contact (dashed black line, S170) between two β sheets. Studies of the C1 domain of Protein Kinase Cδ suggest that these two β sheets are pulled apart to

enable ligand binding. **E and F:** I221 (wild-type, dark blue) and N221 (red); the mutation N221 is predicted to result in an additional polar contact (dashed black line) with K223 and alteration in residue orientation on comparison to the I221 protein. These residues are within the C1 domain. **G and H:** T319 (wild-type, dark blue) and A319 (red); the mutation A319 is predicted to result in the loss of a polar contact (dashed black line) with T370. A319 is in close proximity to the ATP-binding pocket (light blue), this change in polar contacts may alter the conformation of this cleft. **I and J:** R900 (wild-type, dark blue) and H900 (red); the mutation H900 is predicted to result in the loss of a polar contact (dashed black line) with D826. D826 forms is linked to the accessory domain ATP-binding motif (light blue).

Supplementary Table S1: Inclusion criteria for kidney stone disease association analyses in the UK Biobank.

Coding	Code	Definition
International Classification of Diseases (ICD)	ICD9- 7880	Renal colic
	ICD9- 5920	Calculus of kidney
	ICD9- 5920A	Calculus of kidney
	ICD9- 5921	Calculus of ureter
	ICD9- 5929	Urinary calculus, unspecified
	ICD10-N20.0	Calculus of kidney
	ICD10-N20.1	Calculus of ureter
	ICD10-N20.2	Calculus of kidney with calculus of ureter
	ICD10-N20.9	Urinary calculus, unspecified
	ICD10-N23	Unspecified renal colic
Classification of Interventions and Procedures (OPCS)	OPCS3- 563.1	Removal of renal calculus : nephrolithotomy
	OPCS3- 563.2	Removal of renal calculus : pyelolithotomy
	OPCS3- 563.3	Removal of renal calculus : removal without incision
	OPCS3- 580	Ureterolithotomy
	OPCS3- 587.2	Cystoscopic operation on ureter : lithotomy
	OPCS4- M06.1	Open removal of calculus from kidney
	OPCS4- M07.1	Ureteroscopic laser fragmentation of calculus of kidney
	OPCS4- M07.2	Ureteroscopic extraction of calculus of kidney NEC
	OPCS4- M09	Therapeutic endoscopic operations on calculus of kidney
	OPCS4- M09.1	Endoscopic ultrasound fragmentation of calculus of kidney
	OPCS4- M09.2	Endoscopic electrohydraulic shockwave fragmentation of calculus of kidney
	OPCS4- M09.3	Endoscopic laser fragmentation of calculus of kidney
	OPCS4- M09.4	Endoscopic extraction of calculus of kidney NEC
	OPCS4- M09.8	Other specified
	OPCS4- M09.9	Unspecified
	OPCS4- M14	Extracorporeal fragmentation of calculus of kidney
	OPCS4- M14.1	Extracorporeal shock wave lithotripsy of calculus of kidney
	OPCS4- M14.8	Other specified
	OPCS4- M14.9	Unspecified
	OPCS4- M16·4	Percutaneous nephrolithotomy
	OPCS4- M26.1	Nephroscopic laser fragmentation of calculus of ureter
	OPCS4- M26.2	Nephroscopic fragmentation of calculus of ureter NEC
	OPCS4- M26.3	Nephroscopic extraction of calculus of ureter
	OPCS4- M27.1	Ureteroscopic laser fragmentation of calculus of ureter

	OPCS4-M27.2	Ureteroscopic fragmentation of calculus of ureter NEC
	OPCS4-M27.3	Ureteroscopic extraction of calculus of ureter
	OPCS4- M28	Other endoscopic removal of calculus from ureter
	OPCS4-M28·1	Endoscopic laser fragmentation of calculus of ureter
	OPCS4-M28·2	Endoscopic fragmentation of calculus of ureter
	OPCS4-M28·3	Endoscopic extraction of calculus of ureter
	OPCS4-M28.4	Endoscopic catheter drainage of calculus of ureter
	OPCS4-M28.5	Endoscopic drainage of calculus of ureter by dilation of ureter
	OPCS4-M28.8	Other specified
	OPCS4-M28.9	Unspecified
	OPCS4- M31	Extracorporeal fragmentation of calculus of ureter
	OPCS4-M31.1	Extracorporeal shockwave lithotripsy of calculus of ureter
	OPCS4-M31.8	Other specified
	OPCS4-M31.9	Unspecified
Self-reported operation	1197	Percutaneous/open kidney stone surgery/lithotripsy
Death codes	N20.0	Calculus of kidney
	N20.1	Calculus of ureter
	N20.2	Calculus of kidney with calculus of ureter
	N20.9	Urinary calculus, unspecified
	N23	Unspecified renal colic
Primary care Read V2 and V3 codes	Read V2-14D3.	H/O: urinary stone
	Read V2-1A54.	Ureteric colic
	Read V2-4G6..	O/E - ureteric calculus
	Read V2-7B07.	Percutaneous renal stone surgery
	Read V2-7B070	Nephroscopy and ultrasound lithotripsy of renal calculus
	Read V2-7B071	Nephroscopy and electrohydraulic lithotripsy of renal calculus
	Read V2-7B072	Nephroscopy and laser lithotripsy of renal calculus
	Read V2-7B073	Percutaneous nephrolithotomy without disintegration
	Read V2-7B074	Endoscopic extraction of calculus of kidney nec
	Read V2-7B07y	Other specified percutaneous renal stone surgery
	Read V2-7B07z	Percutaneous renal stone surgery NOS
	Read V2-7B0B.	Extracorporeal shockwave lithotripsy for renal calculus
	Read V2-7B0B0	Extracorporeal shockwave lithotripsy for renal calculus of unspecified size

	Read V2-7B0B1	Extracorporeal shockwave lithotripsy for renal calculus less than 2 cm in diameter
	Read V2-7B0B2	Extracorporeal shockwave treatment for renal calculus of 2 cm or more in diameter
	Read V2-7B0By	Other specified extracorporeal shockwave lithotripsy for renal calculus
	Read V2-7B0Bz	Extracorporeal shockwave lithotripsy for renal calculus NOS
	Read V2-7B170	Nephroscopic laser lithotripsy of ureteric calculus
	Read V2-7B171	Other nephroscopic fragmentation of ureteric calculus
	Read V2-7B172	Nephroscopic extraction of ureteric calculus
	Read V2-7B18.	Ureteroscopic operations for ureteric calculus
	Read V2-7B180	Ureteroscopic laser lithotripsy of ureteric calculus
	Read V2-7B181	Other ureteroscopic fragmentation of ureteric calculus
	Read V2-7B182	Ureteroscopic extraction of ureteric calculus
	Read V2-7B19.	Cystoscopic removal of ureteric calculus
	Read V2-7B190	Cystoscopic laser lithotripsy of ureteric calculus
	Read V2-7B191	Other cystoscopic fragmentation of ureteric calculus
	Read V2-7B193	Cystoscopic catheter drainage for ureteric calculus
	Read V2-7B194	Cystoscopic dilation of ureter for drainage of calculus
	Read V2-7B19y	Other specified cystoscopic removal of ureteric calculus
	Read V2-7B19z	Cystoscopic removal of ureteric calculus NOS
	Read V2-7B1C.	Extracorporeal shockwave lithotripsy of ureteric calculus
	Read V2-7B1C0	Extracorporeal shockwave lithotripsy of unspecified ureteric calculus
	Read V2-7B1C1	Extracorporeal shockwave therapy for stone in upper ureter
	Read V2-7B1C2	Extracorporeal shockwave lithotripsy for stone in mid-ureter
	Read V2-7B1C3	Extracorporeal shockwave lithotripsy for stone in lower ureter
	Read V2-7B1Cy	Other specified extracorporeal shockwave lithotripsy of ureteric calculus
	Read V2-7B1Cz	Extracorporeal shockwave lithotripsy of ureteric calculus NOS
	Read V2-C3411	Uric acid nephrolithiasis
	Read V2-K112.	Hydronephrosis with renal and ureteral calculous obstruction
	Read V2-K12..	Calculus of kidney and ureter
	Read V2-K120.	Calculus of kidney

	Read V2-K1200	Staghorn calculus
	Read V2-K120z	Renal calculus NOS
	Read V2-K121.	Calculus of ureter
	Read V2-K122.	Calculus of kidney with calculus of ureter
	Read V2-K12z.	Urinary calculus NOS
	Read V2-Kyu3.	[X]Urolithiasis
	Read V2-R080.	[D]Renal colic
	Read V2-R0800	[D]Renal colic, unspecified
	Read V2-R0801	[D]Ureteric colic
	Read V2-R080z	[D]Renal colic NOS
	Read V3-14D3.	H/O: urinary stone
	Read V3-4G6..	O/E - ureteric calculus
	Read V3-7B07.	Percutaneous nephrolithotomy
	Read V3-7B070	Nephroscopy and ultrasound lithotripsy of renal calculus
	Read V3-7B071	Nephroscopy and electrohydraulic lithotripsy of renal calculus
	Read V3-7B072	Endoscopic laser fragmentation of renal calculus
	Read V3-7B073	Percutaneous nephrolithotomy without disintegration
	Read V3-7B07y	Other specified percutaneous renal stone surgery
	Read V3-7B07z	Percutaneous renal stone surgery NOS
	Read V3-7B0B.	Extracorporeal shockwave lithotripsy for renal calculus
	Read V3-7B0B0	Extracorporeal shockwave lithotripsy for renal calculus of unspecified size
	Read V3-7B0B1	Extracorporeal shockwave lithotripsy for renal calculus less than 2 cm in diameter
	Read V3-7B0B2	Extracorporeal shockwave treatment for renal calculus of 2 cm or more in diameter
	Read V3-7B0By	Other specified extracorporeal shockwave lithotripsy for renal calculus
	Read V3-7B0Bz	Extracorporeal shockwave lithotripsy for renal calculus NOS
	Read V3-7B170	Nephroscopic laser fragmentation of ureteric calculus
	Read V3-7B171	Other nephroscopic fragmentation of ureteric calculus
	Read V3-7B172	Nephroscopic removal of ureteric calculus
	Read V3-7B18.	Ureteroscopic operations for ureteric calculus

	Read V3-7B180	Ureteroscopic laser lithotripsy of ureteric calculus
	Read V3-7B181	Other ureteroscopic fragmentation of ureteric calculus
	Read V3-7B182	Ureteroscopic extraction of ureteric calculus
	Read V3-7B19.	Cystoscopic operation for ureteric calculus
	Read V3-7B190	Cystoscopic laser lithotripsy of ureteric calculus
	Read V3-7B191	Other cystoscopic fragmentation of ureteric calculus
	Read V3-7B193	Cystoscopic catheter drainage for ureteric calculus
	Read V3-7B194	Cystoscopic dilation of ureter for drainage of calculus
	Read V3-7B19y	Other specified cystoscopic removal of ureteric calculus
	Read V3-7B19z	Cystoscopic removal of ureteric calculus NOS
	Read V3-7B1C.	Extracorporeal shockwave lithotripsy of ureteric calculus
	Read V3-7B1C0	Extracorporeal shockwave lithotripsy of unspecified ureteric calculus
	Read V3-7B1C1	Extracorporeal shockwave therapy for stone in upper ureter
	Read V3-7B1C2	Extracorporeal shockwave lithotripsy for stone in mid-ureter
	Read V3-7B1C3	Extracorporeal shockwave lithotripsy for stone in lower ureter
	Read V3-7B1Cy	Other specified extracorporeal shockwave lithotripsy of ureteric calculus
	Read V3-7B1Cz	Extracorporeal shockwave lithotripsy of ureteric calculus NOS
	Read V3-C3411	Renal stone - uric acid
	Read V3-K112.	Hydronephrosis with renal and ureteral calculous obstruction
	Read V3-K1200	Staghorn calculus
	Read V3-K120z	Renal calculus NOS
	Read V3-K121.	Calculus of ureter
	Read V3-K12z.	Urinary calculus NOS
	Read V3-R080.	[D]Renal colic
	Read V3-R0800	[D]Renal colic, unspecified
	Read V3-R0801	[D]Ureteric colic
	Read V3-R080z	[D]Renal colic NOS
	Read V3-X30Pl	Urolithiasis
	Read V3-X30Pm	Urinary calculus

	Read V3-X30Pn	Nephrolithiasis NOS
	Read V3-Xa07P	C/O - ureteric pain
	Read V3-Xa6m6	Ureteroscopic operation for ureteric calculus
	Read V3-Xa8P3	Cystoscopic extraction of ureteric calculus without disintegration
	Read V3-XE0dj	Calculus of kidney and ureter
	Read V3-XE0dk	Kidney calculus
	Read V3-XE0G7	Ureteroscopic removal of ureteric calculus
	Read V3-XE0G8	Cystoscopic extraction of ureteric calculus
	Read V3-XE2Pu	Ureteric colic
	Read V3-XM0CQ	C/O - ureteric colic

Supplementary Table S2: Variants associated with kidney stone disease at genome-wide association study in the UK Biobank.

Variant	CHR	POS	Candidate gene	EA	NEA	EAF	Conditional OR (95% CI)	Conditional P
rs77362499	1	21836204	*ALPL*	G	C	0.11	1.23 (1.17-1.28)	2.70x10-20
rs1256332	1	21893344	*ALPL*	A	C	0.16	1.17 (1.13-1.21)	3.81x10-17
rs838717	2	234296444	*DGKD*	G	A	0.43	1.1 (1.07-1.13)	2.60x10-11
rs10051765	5	176799992	*SLC34A1*	C	T	0.33	1.16 (1.13-1.19)	1.30x10-24
rs7774646	6	30760454	*FLOT1**	T	G	0.17	1.11 (1.07-1.15)	2.30x10-9
rs1155347	6	39146230	*KCNK5*	C	T	0.22	1.13 (1.09-1.16)	4.40x10-13
rs74348938	6	160614649	*SLC22A2*	A	G	0.03	1.28 (1.18-1.38)	5.30x10-10
rs5883088	7	27610433	*HIBADH*	G	GC	0.33	1.11 (1.08-1.14)	2.80x10-12
rs4252512	7	142605221	*TRPV5*	C	T	0.02	1.34 (1.22-1.49)	7.70x10-9
rs7124537	11	10505718	*AMPD3*	C	A	0.09	1.15 (1.1-1.2)	5.40x10-9
rs1182959	13	42674601	*DGKH*	A	G	0.18	1.14 (1.1-1.18)	1.30x10-13
rs76594840	15	47900177	*SEMA6D*	T	C	0.92	1.15 (1.09-1.21)	3.80x10-8
rs112414002	16	16284360	*ABCC6**	C	T	0.97	1.27 (1.16-1.38)	4.40x10-8
rs77924615	16	20392332	*UMOD*	A	G	0.20	1.15 (1.11-1.19)	7.50x10-16
rs9902482	17	70356993	*SOX9*	C	T	0.50	1.08 (1.05-1.11)	1.10x10-8
rs17216707	20	52732362	*CYP24A1*	T	C	0.81	1.16 (1.12-1.2)	9.70x10-18
rs219772	21	37835347	*CLDN14*	A	T	0.74	1.17 (1.13-1.21)	2.50x10-24
rs13054904	22	23410918	*GNAZ*	A	T	0.26	1.14 (1.11-1.18)	7.00x10-18

CHR = chromosome; POS = position based on NCBI Genome Build 37 (hg19); EA= effect allele; NEA= Non-effect allele; EAF= effect allele frequency; OR= odds ratio; P= P-value; 95% CI= 95% confidence interval, *denotes novel association.

Supplementary Table S3: Heritability estimates for GWAS in UK Biobank and in UK Biobank-FinngenR12 GWAS meta-analysis.

	UK Biobank GWAS	UK Biobank-FinngenR12 GWAS meta-analysis
N SNPs	1,154,335	1,094,306
N cases	11,186	24,167
N controls	390,488	876,673
$h2_{SNP}$- liability scale (SE)	0.19 (0.02)	0.18 (0.01)
LDSC intercept (SE)	1.01 (0.01)	1.05 (0.01)
LDSC ratio (SE)	0.09 (0.5)	0.13 (0.03)
Mean χ^2	1.16	1.38

Estimates are calculated using LD-score regression (LDSC) at a population prevalence of 10%. $h2_{SNP}$= mean SNP-based heritability; N SNPs= number of SNPs analyzed; N cases= number of cases; N controls= number of controls; SE= standard error

Supplementary Table S4: Variants associated with kidney stone disease from meta-analysis of genome-wide association studies in the UK Biobank and FinnGenR12 study.

Variant							Meta-analysis		UK Biobank		FinnGenR12		Heterogeneity	
SNP	CHR	POS	Candidate gene	EA	NEA	EAF	OR (95% CI)	P	OR (95% CI)	P	OR (95% CI)	P	I2	P
rs880315	1	10796866	*CASZ1**	C	T	0.38	1.06 (1.04-1.08)	8.66x10-9	1.07 (1.04-1.1)	2.30x10-6	1.05 (1.02-1.07)	4.82x10-4	29.4	0.23
rs115239632	1	21826530	*ALPL*	T	C	0.05	1.33 (1.27-1.38)	4.06x10-44	1.33 (1.24-1.43)	7.10x10-16	1.32 (1.26-1.39)	6.72x10-30	0.0	0.90
rs1256332	1	21893344	*ALPL*	A	C	0.14	1.15 (1.12-1.18)	1.00x10-24	1.16 (1.12-1.21)	1.80x10-16	1.10 (1.06-1.15)	6.67x10-7	73.6	0.05
rs116804195	1	43201576	*CLDN19**	T	C	0.02	1.23 (1.15-1.31)	4.07x10-9	1.22 (1.09-1.35)	3.60x10-4	1.24 (1.13-1.35)	2.88x10-6	0.0	0.82
rs77663766	1	150686011	*HORMAD1**	A	C	0.07	1.11 (1.07-1.14)	1.53x10-8	1.08 (1.02-1.15)	0.01	1.12 (1.07-1.17)	4.33x10-7	0.0	0.40
rs112851199	1	186621231	*PTGS2*	C	T	0.02	1.25 (1.18-1.33)	3.02x10-13	1.23 (1.12-1.34)	6.60x10-6	1.27 (1.17-1.38)	8.23x10-9	0.0	0.57
rs2383517	1	186654633	*PTGS2*	A	T	0.89	1.10 (1.07-1.13)	2.78x10-10	1.06 (1.02-1.1)	4.20x10-3	1.27 (1.17-1.38)	8.42x10-9	0.0	0.53
rs823101	1	205667006	*SLC41A1*	C	T	0.46	1.06 (1.04-1.08)	7.28x10-11	1.06 (1.03-1.09)	3.40x10-5	1.07 (1.04-1.09)	4.58x10-7	0.0	0.69
rs884127	1	220082150	*SLC30A10*	G	A	0.59	1.08 (1.06-1.1)	2.80x10-15	1.08 (1.05-1.11)	7.70x10-8	1.08 (1.05-1.1)	6.99x10-9	0.0	0.89
rs780093	2	27742603	*GCKR*	T	C	0.37	1.07 (1.05-1.09)	2.19x10-11	1.07 (1.05-1.1)	2.40x10-7	1.06 (1.03-1.09)	1.54x10-5	0.0	0.44
rs10171517	2	28090059	*RBKS**	C	T	0.76	1.07 (1.05-1.09)	1.03x10-9	1.08 (1.04-1.11)	4.00x10-6	1.06 (1.03-1.09)	4.77x10-5	0.0	0.46
rs232573	2	38388481	*CYP1B1**	A	G	0.48	1.05 (1.04-1.07)	7.63x10-9	1.05 (1.02-1.08)	4.80x10-4	1.06 (1.03-1.09)	3.46x10-6	0.0	0.53
rs1430083	2	43448479	*THADA*	A	T	0.76	1.08 (1.06-1.11)	2.79x10-11	1.10 (1.06-1.15)	8.30x10-7	1.07 (1.04-1.1)	2.96x10-6	44.7	0.18
rs838717	2	234296444	*DGKD*	G	A	0.42	1.10 (1.08-1.12)	3.08x10-24	1.10 (1.07-1.13)	2.60x10-11	1.10 (1.08-1.13)	1.71x10-14	0.0	0.70
rs74780677	3	48601774	*COL7A1**	A	G	0.94	1.23 (1.18-1.29)	5.58x10-19	1.14 (1.02-1.27)	0.02	1.26 (1.19-1.32)	2.04x10-18	62.7	0.10
rs34594364	3	55461778	*WNT5AA**	AT	A	0.79	1.10 (1.07-1.14)	2.07x10-9			1.10 (1.07-1.14)	2.06x10-9	0.0	1.00
rs13059018	3	55473083	*WNT5AA**	G	C	0.91	1.11 (1.07-1.15)	3.38x10-9	1.08 (1.02-1.14)	0.01	1.13 (1.08-1.17)	5.03x10-8	27.1	0.24

rs34172859	3	121942713	*CASR*	G	GA	0.28	1.08 (1.06-1.10)	2.03x10-14	1.09 (1.06-1.12)	6.20x10-8	1.08 (1.05-1.11)	5.72x10-8	0.0	0.62
rs61171818	3	124734646	*HEG1**	A	C	0.54	1.06 (1.04-1.08)	3.68x10-10	1.06 (1.03-1.09)	2.00x10-5	1.06 (1.03-1.09)	6.38x10-6	0.0	0.98
rs369200	4	3743004	*ADRA2C**	T	C	0.45	1.06 (1.04-1.08)	6.14x10-9	1.04 (1.01-1.07)	4.70x10-3	1.07 (1.04-1.1)	1.04x10-7	59.7	0.12
rs2231142	4	89052323	*ABCG2*	T	G	0.10	1.12 (1.09-1.15)	7.79x10-13	1.12 (1.08-1.17)	6.20x10-8	1.12 (1.07-1.17)	2.62x10-6	0.0	0.86
rs10011293	4	115577381	*UGT8*	T	C	0.23	1.11 (1.08-1.13)	4.73x10-20	1.10 (1.06-1.14)	1.30x10-7	1.11 (1.08-1.14)	5.16x10-14	0.0	0.54
rs66512073	5	51163093	*ISL1**	T	C	0.37	1.06 (1.04-1.08)	1.44x10-9	1.04 (1.02-1.07)	2.00x10-3	1.07 (1.05-1.1)	6.49x10-8	52.5	0.15
rs11959132	5	58605916	*PDE4D**	G	C	0.75	1.06 (1.04-1.08)	2.98x10-8	1.06 (1.02-1.09)	5.80x10-4	1.07 (1.04-1.1)	1.27x10-5	0.0	0.68
rs638333	5	72419267	*TMEM171*	C	T	0.27	1.08 (1.05-1.1)	2.92x10-12	1.06 (1.03-1.09)	1.50x10-4	1.09 (1.06-1.12)	1.77x10-9	47.0	0.17
rs10051765	5	176799992	*SLC34A1*	C	T	0.37	1.14 (1.12-1.16)	6.45x10-43	1.16 (1.13-1.19)	1.30x10-24	1.13 (1.1-1.15)	1.94x10-20	53.6	0.14
rs9461633	6	30761168	*HCG20**	G	A	0.18	1.11 (1.07-1.15)	2.89x10-9	1.11 (1.07-1.15)	2.90x10-9			0.0	1.00
rs9271375	6	32587067	*HLA-DQA1**	G	A	0.56	1.07 (1.05-1.09)	1.82x10-13	1.08 (1.05-1.11)	6.40x10-8	1.07 (1.04-1.09)	5.26x10-7	0.0	0.64
rs12190363	6	39180487	*KCNK5*	A	G	0.27	1.07 (1.05-1.09)	1.20x10-10	1.09 (1.05-1.12)	1.40x10-7	1.06 (1.03-1.08)	7.48x10-5	48.9	0.16
rs68137036	6	43820215	*VEGFA*	G	A	0.30	1.07 (1.05-1.09)	1.24x10-10	1.06 (1.03-1.09)	4.60x10-5	1.07 (1.04-1.1)	6.04x10-7	0.0	0.73
rs2206271	6	50786008	*TFAP2B**	A	T	0.32	1.06 (1.04-1.08)	3.95x10-10	1.05 (1.02-1.08)	9.50x10-4	1.07 (1.05-1.1)	5.61x10-8	21.9	0.26
rs2465043	6	51180765	*PHKD1*	G	A	0.63	1.06 (1.04-1.08)	2.13x10-8	1.04 (1.02-1.07)	2.20x10-3	1.07 (1.04-1.09)	1.62x10-6	0.2	0.32
rs148684631	6	90121976	*RRAGD*	G	GGAGA	0.68	1.06 (1.04-1.08)	1.90x10-9	1.08 (1.05-1.11)	4.40x10-7	1.05 (1.02-1.08)	3.75x10-4	51.9	0.15
rs6928418	6	101175347	*ASCC3*	T	C	0.55	1.06 (1.04-1.08)	3.92x10-10	1.06 (1.04-1.09)	4.40x10-6	1.06 (1.03-1.08)	1.94x10-5	0.0	0.67
rs7740107	6	130374461	*L3MBTL3*	A	T	0.74	1.08 (1.06-1.1)	1.65x10-12	1.07 (1.04-1.1)	1.20x10-5	1.08 (1.05-1.12)	2.36x10-8	0.0	0.51
rs12190287	6	134214525	*TCF21**	C	G	0.59	1.05 (1.03-1.07)	2.12x10-8	1.07 (1.04-1.1)	3.40x10-6	1.04 (1.02-1.07)	8.07x10-4	27.0	0.24
rs74495751	6	160624947	*SLC22A2*	C	G	0.03	1.24 (1.15-1.33)	2.11x10-8	1.24 (1.15-1.33)	2.10x10-8			0.0	1.00
rs1404278	7	27634726	*HIBADH*	T	C	0.31	1.09 (1.07-1.12)	3.12x10-19	1.11 (1.07-1.14)	1.00x10-11	1.08 (1.06-1.11)	3.19x10-9	0.0	0.32
rs1004317	7	30956858	*AQP1*	G	A	0.39	1.07 (1.05-1.09)	1.16x10-11	1.06 (1.04-1.09)	8.00x10-6	1.07 (1.04-1.1)	3.15x10-7	0.0	0.81
rs4252512	7	142605221	*TRPV5*	C	T	0.02	1.34 (1.22-1.49)	7.68x10-9	1.34 (1.22-1.49)	7.70x10-9			0.0	1.00

rs6464165	7	151413124	*PRKAG2**	T	C	0.74	1.07 (1.05-1.09)	9.80x10-11	1.08 (1.05-1.11)	5.10x10-7	1.06 (1.03-1.1)	3.77x10-5	0.0	0.55
rs4745107	9	71177110	*TMEM252**	A	G	0.73	1.06 (1.04-1.09)	2.57x10-9	1.05 (1.02-1.08)	8.30x10-4	1.08 (1.05-1.11)	2.89x10-7	27.3	0.24
rs148349564	9	77466700	*TRPM6*	C	T	0.07	1.12 (1.08-1.16)	1.74x10-10	1.14 (1.08-1.2)	2.80x10-7	1.11 (1.05-1.16)	1.07x10-4	0.0	0.42
rs143845824	9	97474225	*AOPEP*	C	CA	0.91	1.11 (1.08-1.15)	4.37x10-10	1.10 (1.04-1.16)	1.00x10-3	1.12 (1.08-1.17)	9.08x10-8	0.0	0.52
rs2477001	10	34962926	*PARD3**	C	A	0.28	1.06 (1.04-1.08)	5.43x10-9	1.06 (1.03-1.09)	2.30x10-4	1.07 (1.04-1.1)	5.16x10-6	0.0	0.56
rs61264398	11	10474497	*AMPD3*	G	T	0.56	1.06 (1.04-1.08)	3.30x10-11	1.07 (1.04-1.1)	5.60x10-7	1.06 (1.03-1.09)	1.10x10-5	0.0	0.53
rs10789841	11	111469582	*SIK2**	T	C	0.75	1.07 (1.04-1.09)	1.48x10-9	1.08 (1.05-1.11)	4.80x10-7	1.05 (1.02-1.09)	4.17x10-4	19.2	0.27
rs7133836	12	42861098	*PRICKLE1**	C	T	0.43	1.06 (1.04-1.08)	2.66x10-10	1.05 (1.02-1.08)	4.10x10-4	1.07 (1.04-1.1)	9.40x10-8	8.2	0.30
rs73187207	13	42619919	*DGKH*	T	G	0.21	1.07 (1.05-1.1)	8.72x10-10	1.08 (1.04-1.11)	6.50x10-6	1.07 (1.04-1.11)	4.11x10-6	0.0	0.92
rs12585865	13	42751707	*DGKH*	C	T	0.40	1.08 (1.06-1.1)	1.51x10-14	1.10 (1.07-1.13)	2.70x10-11	1.06 (1.03-1.08)	1.65x10-5	74.3	0.05
rs57719175	13	96175396	*CLDN10*	G	A	0.60	1.06 (1.04-1.08)	7.05x10-10	1.05 (1.02-1.08)	7.70x10-4	1.07 (1.04-1.1)	1.13x10-7	31.1	0.23
rs11851035	14	30678761	*PRKD1**	G	A	0.85	1.09 (1.06-1.12)	4.11x10-10	1.12 (1.07-1.17)	1.50x10-7	1.07 (1.03-1.1)	1.60x10-4	64.2	0.09
rs7181593	15	51146114	*AP4E1**	C	T	0.29	1.06 (1.04-1.08)	1.14x10-8	1.07 (1.04-1.1)	1.30x10-5	1.05 (1.02-1.08)	1.82x10-4	0.0	0.49
rs11629962	15	85530028	*PDE8A**	T	C	0.26	1.06 (1.04-1.08)	3.47x10-8	1.07 (1.04-1.11)	7.60x10-6	1.05 (1.02-1.08)	6.84x10-4	13.0	0.28
rs12921916	16	20407196	*UMOD*	C	T	0.28	1.09 (1.07-1.11)	2.88x10-16	1.10 (1.07-1.13)	2.60x10-10	1.08 (1.05-1.11)	1.30x10-7	0.0	0.34
rs889299	16	23381914	*SCNN1B*	G	A	0.78	1.07 (1.04-1.09)	2.84x10-8	1.06 (1.02-1.09)	6.00x10-4	1.07 (1.04-1.11)	9.67x10-6	0.0	0.46
rs11642015	16	53802494	*FTO*	T	C	0.41	1.06 (1.04-1.08)	1.13x10-9	1.05 (1.02-1.08)	8.70x10-4	1.07 (1.04-1.1)	1.84x10-7	17.2	0.27
rs4782364	16	88516135	*ZFPM1*	G	C	0.41	1.06 (1.04-1.08)	1.70x10-9	1.04 (1.02-1.07)	1.50x10-3	1.07 (1.04-1.1)	1.19x10-7	43.6	0.18
rs2240554	17	12264477	*MAP2K4**	T	G	0.54	1.05 (1.03-1.07)	4.66x10-8	1.05 (1.02-1.08)	4.60x10-4	1.05 (1.03-1.08)	2.65x10-5	0.0	0.77
rs11868029	17	37650304	*CDK12**	A	G	0.22	1.07 (1.05-1.1)	1.54x10-10	1.08 (1.05-1.12)	3.40x10-7	1.07 (1.03-1.1)	8.54x10-5	0.0	0.49
rs12150247	17	43480759	*ARHGAP27**	C	G	0.91	1.10 (1.06-1.13)	2.88x10-8	1.04 (0.99-1.09)	0.14	1.04 (1-1.09)	0.08	0.0	0.93
rs111443054	17	43489161	*ARHGAP27**	TCACACA	T	0.15	1.09 (1.06-1.12)	1.46x10-8	1.06 (1.03-1.1)	5.40x10-4	1.11 (1.05-1.17)	2.00x10-4	37.4	0.21
rs2532340	17	44345653	*ARL17B**	G	A	0.18	1.08 (1.05-1.1)	4.92x10-8	1.07 (1.04-1.11)	3.40x10-5	1.08 (1.04-1.13)	3.74x10-4	0.0	0.67

rs117518238	17	59462062	*BCAS3*	C	T	0.92	1.19 (1.14-1.25)	1.12x10-12			1.19 (1.14-1.25)	1.12x10-12	0.0	1.00
rs1058004	17	59486732	*BCAS3*	T	C	0.73	1.07 (1.05-1.09)	5.32x10-11	1.06 (1.02-1.09)	4.50x10-4	1.09 (1.06-1.12)	9.57x10-9	44.6	0.18
rs9902482	17	70356993	*SOX9*	C	T	0.48	1.08 (1.06-1.1)	4.76x10-17	1.08 (1.05-1.11)	1.10x10-8	1.08 (1.05-1.11)	8.16x10-10	0.0	0.99
rs11669940	19	4336379	*STAP2*	C	G	0.68	1.06 (1.04-1.08)	9.56x10-10	1.07 (1.04-1.1)	2.60x10-6	1.06 (1.03-1.08)	6.41x10-5	0.0	0.43
rs3760702	19	14588237	*PTGER1**	A	G	0.29	1.07 (1.05-1.09)	1.57x10-10	1.08 (1.05-1.11)	4.10x10-7	1.06 (1.03-1.09)	6.78x10-5	0.0	0.45
rs11672660	19	46180184	*GIPR*	C	T	0.77	1.07 (1.05-1.09)	5.51x10-10	1.07 (1.03-1.1)	1.40x10-4	1.07 (1.04-1.11)	9.42x10-7	0.0	0.77
rs7259073	19	53357172	*ZNF28**	T	C	0.91	1.11 (1.07-1.15)	4.58x10-9	1.10 (1.04-1.16)	7.40x10-4	1.11 (1.07-1.16)	1.48x10-6	0.0	0.66
rs1932940	20	14604464	*MACROD2**	T	G	0.63	1.06 (1.04-1.08)	2.40x10-8	1.07 (1.04-1.1)	1.40x10-6	1.04 (1.02-1.07)	1.67x10-3	49.7	0.16
rs6127099	20	52731402	*CYP24A1*	A	T	0.73	1.16 (1.13-1.18)	2.06x10-42	1.14 (1.1-1.17)	1.60x10-16	1.17 (1.14-1.21)	4.22x10-28	60.3	0.11
rs2585442	20	52737123	*CYP24A1*	G	C	0.29	1.08 (1.06-1.1)	1.54x10-13	1.11 (1.08-1.14)	7.70x10-11	1.12 (1.09-1.15)	8.81x10-18	0.0	0.56
rs2823254	21	16783083	*NRIP1**	A	T	0.73	1.06 (1.04-1.08)	1.65x10-8	1.05 (1.02-1.09)	3.00x10-4	1.07 (1.04-1.1)	1.20x10-5	0.0	0.56
rs128494	21	37834258	*CLDN14*	C	T	0.69	1.07 (1.05-1.1)	5.66x10-12	1.02 (0.99-1.06)	0.16	1.02 (0.99-1.05)	0.17	0.0	0.84
rs219772	21	37835347	*CLDN14*	A	T	0.76	1.18 (1.15-1.21)	1.79x10-50	1.17 (1.13-1.21)	2.50x10-24	1.19 (1.15-1.23)	5.55x10-28	0.0	0.42
rs5996460	22	23393090	*GNAZ*	A	G	0.25	1.10 (1.08-1.12)	2.42x10-18	1.12 (1.09-1.15)	1.10x10-13	1.08 (1.05-1.11)	9.85x10-7	64.2	0.09
rs6000889	22	38184851	*H1-0**	C	T	0.37	1.06 (1.04-1.08)	2.99x10-9	1.04 (1.01-1.07)	0.01	1.08 (1.05-1.1)	1.92x10-8	70.7	0.06
rs139495	22	41635425	*CHADL**	C	T	0.71	1.06 (1.04-1.08)	2.85x10-8	1.07 (1.04-1.11)	2.30x10-6	1.05 (1.02-1.07)	1.44x10-3	41.2	0.19

95% CI= 95% confidence interval; CHR= chromosome; EA= effect allele; EAF= effect allele frequency; NEA= non-effect allele; OR= odds ratio; P= p-value; POS= position based on NCBI Genome Build 37 (hg19).*denotes novel association.

Supplementary Table S5: MAGMA gene association test for meta-analysis of genome-wide association studies in the UK Biobank and FinnGenR12 study.

Gene	CHR	N SNP	P-value
DGKD	2	267	3.08x10-15
MXD3	5	15	3.33x10-15
DGKH	13	551	1.71x10-14
SLC30A10	1	30	1.89x10-14
SAG	2	144	2.05x10-14
UGT8	4	156	2.50x10-14
HIBADH	7	497	3.75x10-14
TBX2	17	27	1.94x10-13
PTGS2	1	21	2.40x10-13
RAP1GAP	1	252	3.03x10-13
AQP1	7	35	7.16x10-13
ALPL	1	235	1.64x10-12
ZNF512	2	81	2.92x10-12
PDILT	16	118	5.09x10-12
PRELID1	5	7	6.82x10-12
PTGER1	19	10	7.91x10-12
CASR	3	437	1.16x10-11
RNLS	10	851	1.58x10-11
LSM2	6	27	1.74x10-11
MRPL33	2	13	2.97x10-11
ASCC3	6	1111	4.00x10-11
TMEM89	3	3	4.76x10-11
KCNK5	6	144	5.64x10-11
RBKS	2	234	9.77x10-11
ATG16L1	2	123	1.04x10-10
POU2AF1	11	103	1.06x10-10
CDK12	17	232	1.42x10-10
CLDN10	13	469	2.17x10-10
TFAP2B	6	76	2.61x10-10
FBXL20	17	352	4.85x10-10
RAB29	1	21	4.98x10-10
LMAN2	5	31	5.00x10-10
RGS14	5	22	5.00x10-10
SLC34A1	5	20	5.00x10-10
MSH5	6	52	5.15x10-10
YAF2	12	139	5.83x10-10
MED1	17	90	6.74x10-10
NSD1	5	280	7.24x10-10
CYP24A1	20	92	8.04x10-10
GCKR	2	44	1.04x10-9
VARS	6	30	1.21x10-9
TAX1BP1	7	215	1.69x10-9
ZFPM1	16	355	1.83x10-9
TNFAIP8L3	15	116	2.12x10-9
CLDN14	21	404	2.40x10-9
ZCRB1	12	22	3.29x10-9
GIPR	19	35	4.45x10-9
JAZF1	7	1038	5.39x10-9
TRPV5	7	55	5.94x10-9
FTO	16	1411	6.42x10-9
VWA7	6	29	8.71x10-9
AMPD3	11	213	1.55x10-8
PPP2R1B	11	48	1.61x10-8
TRIOBP	22	229	1.67x10-8
TMEM171	5	43	1.68x10-8
SIK2	11	194	1.69x10-8
KLK15	19	18	1.82x10-8
LY6G6F	6	9	1.84x10-8
CCBL2	1	124	1.90x10-8
MICA	6	253	1.91x10-8
L3MBTL3	6	254	2.00x10-8
GCAT	22	26	2.23x10-8
STAP2	19	79	2.78x10-8
CSNK2B	6	15	3.12x10-8
NUCKS1	1	79	3.98x10-8
AP4E1	15	239	4.23x10-8
PLCL1	2	593	5.72x10-8
PPHLN1	12	335	7.07x10-8
FGFR4	5	25	7.47x10-8
MICB	6	146	7.92x10-8
RAB36	22	69	7.95x10-8
EPRS	1	285	8.44x10-8
CHAF1B	21	118	8.48x10-8
AP1S3	2	250	9.10x10-8
ABHD16A	6	25	1.00x10-7
DXO	6	10	1.09x10-7
PARD3	10	1674	1.09x10-7
TNXB	6	192	1.20x10-7
SLC12A3	16	246	1.23x10-7
SF3B1	2	63	1.43x10-7
DZIP1	13	105	1.53x10-7
SLC15A2	3	69	1.53x10-7
USP50	15	163	1.86x10-7
BCR	22	460	2.25x10-7
PRKAG2	7	1266	2.27x10-7
LST1	6	11	2.90x10-7
FOXL2NB	3	24	3.15x10-7
QPCTL	19	26	3.34x10-7
DDX41	5	7	3.46x10-7
C2	6	121	3.64x10-7
EPHB6	7	42	3.92x10-7
KEL	7	53	4.14x10-7
DDR1	6	63	4.35x10-7
PKN1	19	104	4.93x10-7
ITPK1	14	569	5.03x10-7
TRPV6	7	18	5.39x10-7
STH	17	1	5.65x10-7
AQP4	18	38	5.71x10-7
VPS13B	8	1781	6.23x10-7
CRYAB	11	5	7.93x10-7
CRHR1	17	1074	8.01x10-7
TFEB	6	156	8.38x10-7
PSORS1C1	6	212	9.71x10-7
MAPT	17	785	9.85x10-7
ATP6V1G2	6	8	1.02x10-6
PDE3A	12	1100	1.05x10-6
KANSL1	17	947	1.07x10-6
RSPH14	22	211	1.10x10-6
ARHGAP27	17	108	1.20x10-6
SPPL2C	17	17	1.20x10-6
PDE4D	5	3807	1.21x10-6
PLEKHM1	17	156	1.29x10-6
FAM209A	20	2	1.34x10-6
ITIH1	3	29	1.43x10-6
BRE	2	1126	1.44x10-6
BAG6	6	38	1.59x10-6
SLC14A2	18	1629	1.65x10-6
NEU1	6	2	1.75x10-6
SLC45A3	1	62	1.78x10-6
HSPG2	1	379	1.82x10-6
ITIH3	3	23	1.83x10-6
FDXACB1	11	9	2.00x10-6
TRPM7	15	444	2.08x10-6
DNAH6	2	798	2.10x10-6
CMIP	16	1139	2.15x10-6
USP40	2	184	2.29x10-6
FKBPL	6	5	2.43x10-6
CUL2	10	204	2.50x10-6

CHR= chromosome; N SNP= number of single nucleotide polymorphisms associated with gene included in analysis; P-value= p-value for association of gene.

Supplementary Table S6: MAGMA Gene-set analysis in meta-analysis of genome-wide association studies in the UK Biobank and FinnGenR12 study.

Analysis	Gene-set	N Genes	P-value	Significant genes
Human Phenotype Ontology Gene Ontology	Anion homeostasis	49	2.70x10-6	*ABCC6, ALPL, ATP6V1B1, FGFR4, SLC12A3, SLC17A6, SLC34A1, TFAP2B, UMOD*
	Urate metabolic process	13	4.66x10-6	*ABCG2, GCKR, LRRC16A, SLC17A1, SLC17A3*
	Hypermagnesemia	7	1.40x10-7	*CASR, CLDN10, SLC12A3*
	Hypocalciuria	10	1.42x10-7	*CASR, CLDN10, SLC12A3*
	Apical junction assembly	73	2.71x10-7	*CLDN10, CLDN14, F11R, MYO1C, PARD3, ROCK2, VCL, WDR1*
	Fibroblast growth factor production	6	3.12x10-7	*AIF1, HEG1, PTGS2, ROCK2, WNT11*
	Metanephric loop of Henle development	6	6.20x10-7	*AQP1, PKD2, UMOD*
	Alkaline phosphatase activity	6	6.58x10-7	*ALPI, ALPL, ALPP, ALPPL2*
	Abnormal blood inorganic cation concentration	153	4.43x10-8	*ALG12, ALPL, CASR, CLDN10, CYP24A1, GATA3, KCNJ1, NSD1, PDE4D, RREB1, SEC24C, SLC12A3, SLC30A10, SLC34A1*
Cell-type	Lake adult kidney C3 proximal tubule epithelial cells S1 S2	207	9.26x10-6	*ANKRD11, BCAS3, CALD1, CLDN10, PARD3, PDE4D, PDE8A, PKHD1, SIK2, SLC17A1, SLC17A3, TFDP2, TLN2, WDR72*

Gene-set= gene-set name, ranked by descending P-value; N Genes= number of genes in gene-set; P-value= p-value for association of gene-set with kidney stone disease; Genes= genes in gene-set significantly associated with kidney stone disease, listed in alphabetical order.

Supplementary Table S7: Estimate of bias for Mendelian randomization with sample overlap in the UK Biobank.

Mineral metabolism trait	GWAS sample size	Maximum number of instruments in MR analyses	Estimate of bias
Adjusted calcium	308,679	24	0.011
Phosphate	308,110	20	0.001

The maximum number of instruments was used to derive the most pessimistic estimate of bias. Results are shown for a Type 1 error rate of 0.05.

Supplementary Table S8: Causal effects of lead variants from mineral metabolism GWAS (±500kb) with evidence of colocalization on kidney stone risk.

Exposure: Albumin-adjusted serum calcium concentration																	
Exposure data					Mendelian randomization analysis									Colocalization analysis			
SNP	Gene	Outcome study	CHR	POS	N SNPs	Mean R^2 (SE)	OR (95%CI)	FDR-P	Intercept beta (SE)	Intercept FDR-P	Q	Q_df	P	N SNPs	PP H4	Candidate causal SNP	SNP PP
rs607518	*ANXA9*	Meta-analysis	1	150954671	3	2.20x10-4 (9.56x10-5)	7.06 (4.12-12.11)	1.75x10-11	-0.09 (0.19)	0.99	0.22	2	0.9	1870	0.91	rs267738	0.36
		UK Biobank			4		10.45 (5.28-20.7)	3.24x10-10	-0.11 (0.11)	0.98	1.37	3	0.71	2231	0.98	rs267738	0.44
		FinnGen			3		2.37 (0.4-13.99)	0.46	-0.40 (0.11)	0.97	11.97	2	0	1562	0.03	-	-
rs3011	*SLC30A10*	Meta-analysis	1	220085453	4	1.57x10-4 (2.90x10-5)	18.38 (9.57-35.3)	4.25x10-17	0.05 (0.03)	0.98	3.06	3	0.38	3392	0.99	rs3011	0.19
		UK Biobank			4		22.99 (10.65-49.6)	3.00x10-14	0.03 (0.04)	0.98	1.46	3	0.69	3344	0.98	rs10863512	0.21
rs2675966	*SNORC*	Meta-analysis	2	233735543	4	3.74x10-4 (3.17x10-4)	3.87 (2.71-5.51)	1.27x10-12	-0.01 (0.02)	0.99	2.43	3	0.49	3580	1.00	rs11891546	1.00
		UK Biobank			3		3.04 (1.77-5.23)	2.72x10-4	-0.01 (0.03)	0.98	2.06	2	0.36	3617	0.96	rs11891546	1.00
		FinnGen			4		4.66 (2.9-7.49)	3.05x10-9	-3.69x10-3 (0.02)	0.97	1.57	3	0.67	3087	0.99	rs11891546	1.00
rs838705	*DGKD*	Meta-analysis	2	234273242	10	3.27x10-4 (3.77x10-4)	4.51 (3.38-6.03)	3.31x10-23	-0.03 (0.01)	0.48	14	9	0.12	3303	1.00	rs838717	1.00
		UK Biobank			9		3.89 (2.61-5.79)	3.94x10-10	-0.04 (0.02)	0.98	10.76	8	0.22	3409	1.00	rs838717	1.00
		FinnGen			11		4.93 (3.39-7.19)	1.69x10-15	-0.02 (0.02)	0.97	15.29	10	0.12	2961	1.00	rs838717	1.00
rs838717	*DGKD*	Meta-analysis	2	234296444	10	3.27x10-4 (3.77x10-4)	4.51 (3.38-6.03)	3.31x10-23	-0.03 (0.01)	0.48	14	9	0.12	3265	1.00	rs838717	1.00
		UK Biobank			9		3.89 (2.61-5.79)	3.94x10-10	-0.04 (0.02)	0.98	10.76	8	0.22	3372	1.00	rs838717	1.00
		FinnGen			11		4.93 (3.39-7.19)	1.69x10-15	-0.02 (0.02)	0.97	15.29	10	0.12	2922	1.00	rs838717	1.00
rs79069677	*AOPEP*	Meta-analysis	9	97530059	3	2.56x10-4 (1.85x10-4)	4.06 (1.68-9.79)	0.01	-0.04 (0.02)	0.98	6.87	2	0.03	2862	0.94	rs925813	0.06
		UK Biobank			3		2.48 (0.8-7.7)	0.21	-0.04 (0.02)	0.98	4.96	2	0.08	2999	0.50	-	-
rs1858800	*ZFHX3*	Meta-analysis	16	73024276	4	2.53x10-4 (2.40x10-4)	3.51 (1.73-7.15)	1.63x10-3	8.34x10-4 (0.04)	0.99	8.29	3	0.04	2679	1.00	rs1858800	1.00
		UK Biobank			5		2.12 (0.74-6.06)	0.27	-0.01 (0.05)	0.98	14.7	4	0.01	2811	0.61	-	-
		FinnGen			5		4.63 (2.67-8.04)	6.97x10-7	-4.03x10-3 (0.02)	0.97	1.84	4	0.77	2325	0.97	rs1858800	1.00

rs4782358	*ZFPM1*	Meta-analysis	16	88485506	5	2.66x10-4 (2.80x10-4)	0.31 (0.21-0.46)	4.29x10-8	0.02 (0.02)	0.98	4.46	4	0.35	4679	0.95	rs12918968	0.57
		UK Biobank			5		0.4 (0.23-0.67)	2.53x10-3	-2.45x10-3 (0.02)	0.98	3.52	4	0.47	4809	0.29	-	-
		FinnGen			4		0.22 (0.12-0.38)	6.97x10-7	0.04 (0.04)	0.97	1.79	3	0.62	4090	0.96	rs12918968	0.90
rs12447180	*ZFPM1*	Meta-analysis	16	88517722	5	2.66x10-4 (2.80x10-4)	0.31 (0.21-0.46)	4.29x10-8	0.02 (0.02)	0.98	4.46	4	0.35	4570	0.95	rs12918968	0.87
		UK Biobank			5		0.4 (0.23-0.67)	2.53x10-3	-2.45x10-3 (0.02)	0.98	3.52	4	0.47	4697	0.29	-	-
		FinnGen			4		0.22 (0.12-0.38)	6.97x10-7	0.04 (0.04)	0.97	1.79	3	0.62	3949	0.96	rs12918968	0.84
rs3091842	*MAFB*	Meta-analysis	20	39344272	4	4.60x10-4 (3.57x10-4)	1.89 (1.29-2.76)	2.88x10-3	-2.03x10-3 (0.02)	0.99	5.97	3	0.11	2761	0.94	rs3091842	1.00
		UK Biobank			4		1.79 (1.14-2.81)	0.03	4.64x10-4 (0.02)	0.98	1.65	3	0.65	2697	0.08	-	-
		FinnGen			4		1.95 (1.27-3)	0.01	-2.71x10-3 (0.02)	0.97	4.94	3	0.18	2361	0.79	rs3091842	1.00
rs2425431	*MAFB*	Meta-analysis	20	39352714	4	4.60x10-4 (3.57x10-4)	1.89 (1.29-2.76)	2.88x10-3	-2.03x10-3 (0.02)	0.99	5.97	3	0.11	2754	0.94	rs3091842	1.00
		UK Biobank			4		1.79 (1.14-2.81)	0.03	4.64x10-4 (0.02)	0.98	1.65	3	0.65	2690	0.08	-	-
		FinnGen			4		1.95 (1.27-3)	0.01	-2.71x10-3 (0.02)	0.97	4.94	3	0.18	2351	0.79	rs3091842	1.00
rs209961	*CYP24A1*	Meta-analysis	20	52715154	18	3.29x10-4 (3.08x10-4)	10.36 (8.54-12.56)	1.45x10-123	-0.02 (0.01)	0.48	20.37	17	0.26	3561	1.00	rs6127099	1.00
		UK Biobank			17		8.46 (6.58-10.88)	8.54x10-61	3.29x10-4 (0.01)	0.98	15.74	16	0.47	3647	0.98	rs6127099	0.98
		FinnGen			18		12.69 (9.88-16.31)	1.74x10-86	-0.04 (0.01)	0.10	20.64	17	0.24	3236	1.00	rs6127099	1.00
rs2585442	*CYP24A1*	Meta-analysis	20	52737123	18	3.29x10-4 (3.08x10-4)	10.36 (8.54-12.56)	1.45x10-123	-0.02 (0.01)	0.48	20.37	17	0.26	3591	1.00	rs6127099	1.00
		UK Biobank			17		8.46 (6.58-10.88)	8.54x10-61	3.29x10-4 (0.01)	0.98	15.74	16	0.47	3682	0.98	rs6127099	0.98
		FinnGen			18		12.69 (9.88-16.31)	1.74x10-86	-0.04 (0.01)	0.10	20.64	17	0.24	3262	1.00	rs6127099	1.00
rs117268564	*CYP24A1*	Meta-analysis	20	52738434	18	3.29x10-4 (3.08x10-4)	10.36 (8.54-12.56)	1.45x10-123	-0.02 (0.01)	0.48	20.37	17	0.26	3594	1.00	rs6127099	1.00
		UK Biobank			17		8.46 (6.58-10.88)	8.54x10-61	3.29x10-4 (0.01)	0.98	15.74	16	0.47	3685	0.98	rs6127099	0.98
		FinnGen			18		12.69 (9.88-16.31)	1.74x10-86	-0.04 (0.01)	0.10	20.64	17	0.24	3264	1.00	rs6127099	1.00
rs35194449	*CYP24A1*	Meta-analysis	20	52742047	18	3.29x10-4 (3.08x10-4)	10.36 (8.54-12.56)	1.45x10-123	-0.02 (0.01)	0.48	20.37	17	0.26	3593	1.00	rs6127099	1.00
		UK Biobank			17		8.46 (6.58-10.88)	8.54x10-61	3.29x10-4 (0.01)	0.98	15.74	16	0.47	3684	0.98	rs6127099	0.98
		FinnGen			18		12.69 (9.88-16.31)	1.74x10-86	-0.04 (0.01)	0.10	20.64	17	0.24	3266	1.00	rs6127099	1.00
rs2762943	*CYP24A1*	Meta-analysis	20	52790786	18	3.29x10-4 (3.08x10-4)	10.36 (8.54-12.56)	1.45x10-123	-0.02 (0.01)	0.48	20.37	17	0.26	3599	1.00	rs6127099	1.00
		UK Biobank			17		8.46 (6.58-10.88)	8.54x10-61	3.29x10-4 (0.01)	0.98	15.74	16	0.47	3760	0.98	rs6127099	0.98
		FinnGen			18		12.69 (9.88-16.31)	1.74x10-86	-0.04 (0.01)	0.10	20.64	17	0.24	3340	1.00	rs6127099	1.00

Exposure: Serum phosphate concentration																	
Exposure data					Mendelian randomization analysis									Colocalization analysis			
SNP	Gene	Outcome study	CHR	POS	N SNPs		OR* (95%CI)	FDR-P	Intercept beta (SE)	Intercept P	Q	Q_df	P	N SNPs	PP H4	Candidate causal SNP	SNP PP
rs28383573	*APCS*	Meta-analysis	1	159558672	3	2.80x10-4 (1.05x10-4)	2.22 (1.28-3.83)	0.02	-0.04 (0.03)	0.98	3.45	2	0.18	3268	0.87	rs71628146	0.35
		UK Biobank			3		2.08 (0.71-6.11)	0.35	-0.07 (0.07)	0.99	5.04	2	0.08	3213	0.35	-	-
		FinnGen			3		2.3 (1.36-3.91)	0.02	-0.02 (0.03)	0.98	0.49	2	0.78	2741	0.31	-	-
rs71628146	*APCS*	Meta-analysis	1	159582487	3	2.80x10-4 (1.05x10-4)	2.22 (1.28-3.83)	0.02	-0.04 (0.03)	0.98	3.45	2	0.18	3286	0.87	rs71628146	0.35
		UK Biobank			3		2.08 (0.71-6.11)	0.35	-0.07 (0.07)	0.99	5.04	2	0.08	3232	0.35	-	-
		FinnGen			3		2.3 (1.36-3.91)	0.02	-0.02 (0.03)	0.98	0.49	2	0.78	2754	0.31	-	-
rs838718	*DGKD*	Meta-analysis	2	234296650	4	2.13x10-4 (1.83x10-4)	0.05 (0.03-0.08)	2.06x10-34	0.01 (0.02)	0.98	2.9	3	0.41	3268	0.99	rs838717	0.96
		UK Biobank			4		0.05 (0.02-0.14)	4.88x10-7	-1.56x10-3 (0.05)	0.99	6.99	3	0.07	3378	0.99	rs838718	0.48
		FinnGen			4		0.05 (0.03-0.1)	3.45x10-18	0.01 (0.02)	0.98	1.58	3	0.66	2926	1.00	rs838717	0.73
rs12152922	*SLC34A1*	Meta-analysis	5	176645599	9	2.98x10-4 (3.78x10-4)	0.09 (0.07-0.13)	2.70x10-38	0.01 (0.01)	0.98	14.56	8	0.07	2409	1.00	rs10051765	1.00
		UK Biobank			9		0.07 (0.05-0.11)	9.93x10-40	0.03 (0.01)	0.99	7.54	8	0.48	2582	1.00	rs10051765	0.82
		FinnGen			8		0.12 (0.07-0.18)	1.91x10-19	4.88x10-3 (0.02)	0.98	11.13	7	0.13	2128	1.00	rs10051765	1.00
rs10051765	*SLC34A1*	Meta-analysis	5	176799992	9	2.98x10-4 (3.78x10-4)	0.09 (0.07-0.13)	2.70x10-38	0.01 (0.01)	0.98	14.56	8	0.07	2219	1.00	rs10051765	1.00
		UK Biobank			9		0.07 (0.05-0.11)	9.93x10-40	0.03 (0.01)	0.99	7.54	8	0.48	2359	1.00	rs10051765	0.82
		FinnGen			8		0.12 (0.07-0.18)	1.91x10-19	4.88x10-3 (0.02)	0.98	11.13	7	0.13	2022	1.00	rs10051765	1.00
rs883622	*FAM20A*	Meta-analysis	17	66442603	6	2.57x10-4 (2.36x10-4)	1.68 (0.9-3.13)	0.22	-0.04 (0.01)	0.53	15.82	5	0.01	3189	0.97	rs11867466	0.27
		UK Biobank			6		2.35 (1.43-3.88)	3.94x10-3	-0.02 (0.02)	0.99	2.74	5	0.74	3438	0.61	-	-
		FinnGen			6		1.21 (0.49-2.98)	0.74	-0.06 (0.02)	0.94	16.82	5	0	2930	0.32	-	-
rs11871728	*FAM20A*	Meta-analysis	17	66703728	12	2.56x10-4 (2.32x10-4)	8.40 (4.33-16.29)	0.01	-0.04 (0.01)	0.03	31.64	11	0	3165	0.97	rs11867466	0.27
		UK Biobank			12		2.35 (1.66-3.35)	3.77x10-5	-0.02 (0.01)	0.99	5.49	11	0.91	3261	0.61	-	-
		FinnGen			12		11.36 (4.43-29.1)	0.03	-0.06 (0.01)	0.04	33.64	11	0	2771	0.33	-	-

CHR= chromosome; POS= position based on NCBI Genome Build 37 (hg19); N SNPs= number of SNPs; FDR-P= P-value adjusted for 5% false discovery rate; OR= odds ratio for kidney stone disease per genetically-instrumented standard deviation increase in trait; P= P-value; PP H4=

posterior probability of H4 (full colocalization); Q= Q statistic; Q_df= Q degrees of freedom; SD= standard deviation; SE= standard error; SNP PP= posterior probability explained by SNP; 95% CI= 95% confidence interval

Supplementary Table S9: 95% credible sets for genomic regions with evidence for causal effects on kidney stone risk and colocalization in UK Biobank-FinnGenR12 genome-wide association study meta-analysis.

Adjusted calcium							
SNP	Gene	CHR	POS	N SNPs	PP H4	95% credible set	SNP PP
rs607518	*ANXA9*	1	150954671	1870	0.91	rs267738	0.36
						rs267734	0.32
						rs607518	0.14
						rs198325	0.11
						rs78132593	0.05
rs3011	*SLC30A10*	1	220085453	3392	0.99	rs10863512	0.2
						rs6694088	0.19
						rs6694079	0.17
						rs7533348	0.17
						rs884127	0.15
						rs1891562	0.12
rs2675966	*SNORC*	2	233735543	3580	1.00	rs11891546	1.00
rs838705	*DGKD*	2	234273242	3303	1.00	rs838717	1.00
rs838717	*DGKD*	2	234296444	3265	1.00	rs838717	1.00
rs79069677	*AOPEP*	9	97530059	2862	0.94	rs10993341	0.05
						rs10993340	0.05
						rs75698471	0.05
						rs112045297	0.05
						rs12341764	0.05
						rs12341814	0.05
						rs79069677	0.04
						rs12337706	0.04
						rs10993337	0.04
						rs3808890	0.04
						rs925813	0.04
						rs76491653	0.02
						rs12344157	0.01
						rs12345321	0.01
						rs12352600	0.01
						rs113633127	0.01
						rs12345783	0.01
						rs10993363	0.01
						rs61446634	0.01
						rs16911713	0.01
						rs12346548	0.01
						rs10993360	0.01
						rs141795736	0.01
						rs9987803	0.01
						rs10993349	0.01
						rs7866487	0.01
						rs10993362	0.01
						rs60048957	0.01
						rs139965936	0.01
						rs77820587	0.01
						rs12344256	0.01
						rs10993329	0.01
						rs16911670	0.01
						rs60514919	0.01

						rs12336437	0.01
						rs12343530	0.01
						rs12347744	0.01
						rs10993364	0.01
						rs9987493	0.01
rs1858800	*ZFHX3*	16	73024276	2679	1.00	rs1858800	1.00
rs4782358	*ZFPM1*	16	88485506	4679	0.95	rs12918968	0.87
						rs12447180	0.05
						rs879627	0.02
						rs879628	0.02
rs12447180	*ZFPM1*	16	88517722	4570	0.95	rs12918968	0.87
						rs12447180	0.05
						rs879627	0.02
						rs879628	0.02
rs3091842	*MAFB*	20	39344272	2761	0.94	rs3091842	1.00
rs2425431	*MAFB*	20	39352714	2754	0.94	rs3091842	1.00
rs209961	*CYP24A1*	20	52715154	3561	1.00	rs6127099	1.00
rs2585442	*CYP24A1*	20	52737123	3591	1.00	rs6127099	1.00
rs117268564	*CYP24A1*	20	52738434	3594	1.00	rs6127099	1.00
rs35194449	*CYP24A1*	20	52742047	3593	1.00	rs6127099	1.00
rs2762943	*CYP24A1*	20	52790786	3599	1.00	rs6127099	1.00
Phosphate							
SNP	Gene	CHR	POS	N SNPs	PP H4	Candidate causal SNP	SNP PP
rs28383573	*APCS*	1	159558672	3268	0.87	rs71628146	0.35
						rs77672383	0.05
						rs12059422	0.03
						rs7354905	0.03
						rs12062765	0.03
						rs12061009	0.03
						rs12741812	0.03
						rs71628140	0.03
						rs12084273	0.03
						rs12076447	0.03
						rs12741992	0.02
						rs73030515	0.02
						rs34565293	0.02
						rs12091346	0.02
						rs71628141	0.02
						rs12065183	0.02
						rs12079353	0.02
						rs36126250	0.02
						rs35834732	0.01
						rs12731846	0.01
						rs35085476	0.01
						rs71628138	0.01
						rs35886354	0.01
						rs71628135	0.01
						rs71628136	0.01
						rs71628137	0.01
rs71628146	*APCS*	1	159582487	3286	0.87	rs71628146	0.35
						rs77672383	0.05
						rs12059422	0.03
						rs7354905	0.03
						rs12062765	0.03
						rs12061009	0.03
						rs12741812	0.03
						rs71628140	0.03

						rs12084273	0.03
						rs12076447	0.03
						rs12741992	0.02
						rs73030515	0.02
						rs34565293	0.02
						rs12091346	0.02
						rs71628141	0.02
						rs12065183	0.02
						rs12079353	0.02
						rs36126250	0.02
						rs35834732	0.01
						rs12731846	0.01
						rs35085476	0.01
						rs71628138	0.01
						rs35886354	0.01
						rs71628135	0.01
						rs71628136	0.01
						rs71628137	0.01
rs838718	*DGKD*	2	234296650	3268	0.99	rs838717	0.96
rs12152922	*SLC34A1*	5	176645599	2409	1.00	rs10051765	1.00
rs10051765	*SLC34A1*	5	176799992	2219	1.00	rs10051765	1.00
rs883622	*FAM20A*	17	66442603	3189	0.97	rs11867466	0.27
						rs11871728	0.24
						rs12325975	0.1
						rs12950543	0.1
						rs11077726	0.1
						rs33998125	0.05
						rs11869735	0.03
						rs11650533	0.03
						rs12952992	0.03
rs11871728	*FAM20A*	17	66703728	3165	0.97	rs11867466	0.27
						rs11871728	0.24
						rs12325975	0.1
						rs12950543	0.1
						rs11077726	0.1
						rs33998125	0.05
						rs11869735	0.03
						rs11650533	0.03
						rs12952992	0.03

CHR= chromosome; POS= position based on NCBI Genome Build 37 (hg19); N SNPs= number of SNPs; PP H4= posterior probability of H4 (full colocalization); SNP PP= posterior probability explained by SNP

Supplementary Table S10: 95% credible sets for genomic regions with evidence for causal effects on kidney stone risk and colocalization in the UK Biobank.

Adjusted calcium							
SNP	Gene	CHR	POS	N SNPs	PP H4	Candidate causal SNP	SNP PP
rs607518	*ANXA9*	1	150954671	2231	0.98	rs267738	0.44
						rs267734	0.22
						1:150878099_TC_T	0.11
						rs198325	0.10
						rs607518	0.09
rs3011	*SLC30A10*	1	220085453	3344	0.98	rs3011	0.19
						rs6694079	0.15
						rs10863512	0.15
						rs6694088	0.15
						rs7533348	0.14
						rs884127	0.09
						rs1891562	0.07
						rs11282751	0.04
rs2675966	*SNORC*	2	233735543	3087	0.99	rs11891546	1.00
rs838705	*DGKD*	2	234273242	3409	1.00	rs838717	1.00
rs838717	*DGKD*	2	234296444	3372	1.00	rs838717	1.00
rs209961	*CYP24A1*	20	52715154	3647	0.98	rs6127099	0.98
rs2585442	*CYP24A1*	20	52737123	3682	0.98	rs6127099	0.98
rs117268564	*CYP24A1*	20	52738434	3685	0.98	rs6127099	0.98
rs35194449	*CYP24A1*	20	52742047	3684	0.98	rs6127099	0.98
rs2762943	*CYP24A1*	20	52790786	3760	0.98	rs6127099	0.98
Phosphate							
SNP	Gene	CHR	POS	N SNPs	PP H4	Candidate causal SNP	SNP PP
rs838718	*DGKD*	2	234296650	3378	0.99	rs838718	0.48
						rs838717	0.47
rs12152922	*SLC34A1*	5	176645599	2582	1.00	rs10051765	0.82
						rs56235845	0.18
rs10051765	*SLC34A1*	5	176799992	2359	1.00	rs10051765	0.82
						rs56235845	0.18

CHR= chromosome; POS= position based on NCBI Genome Build 37 (hg19); N SNPs= number of SNPs; PP H4= posterior probability of H4 (full colocalization); SNP PP= posterior probability explained by SNP

Supplementary Table S11: 95% credible sets for genomic regions with evidence for causal effects on kidney stone risk and colocalization in the FinnGenR12 study.

Adjusted calcium							
SNP	Gene	CHR	POS	N SNPs	PP H4	Candidate causal SNP	SNP PP
rs2675966	*SNORC*	2	233735543	3087	0.99	rs11891546	1.00
rs838705	*DGKD*	2	234273242	2961	1.00	rs838717	1.00
rs838717	*DGKD*	2	234296444	2922	1.00	rs838717	1.00
rs1858800	*ZFHX3*	16	73024276	2325	0.97	rs1858800	1.00
rs4782358	*ZFPM1*	16	88485506	4090	0.96	rs12918968	0.90
						rs879627	0.04
						rs879628	0.03
rs12447180	*ZFPM1*	16	88517722	3949	0.96	rs12918968	0.90
						rs879627	0.04
						rs879628	0.03
rs3091842	*MAFB*	20	39344272	2361	0.79	rs3091842	1.00
rs2425431	*MAFB*	20	39352714	2351	0.79	rs3091842	1.00
rs209961	*CYP24A1*	20	52715154	3236	1.00	rs6127099	1.00
rs209961	*CYP24A1*	20	52715154	3236	1.00	rs6127099	1.00
rs2585442	*CYP24A1*	20	52737123	3262	1.00	rs6127099	1.00
rs117268564	*CYP24A1*	20	52738434	3264	1.00	rs6127099	1.00
rs35194449	*CYP24A1*	20	52742047	3266	1.00	rs6127099	1.00
rs2762943	*CYP24A1*	20	52790786	3340	1.00	rs6127099	1.00
Phosphate							
SNP	Gene	CHR	POS	N SNPs	PP H4	Candidate causal SNP	SNP PP
rs838718	*DGKD*	2	234296650	2926	1.00	rs838717	0.73
						rs838718	0.26
rs12152922	*SLC34A1*	5	176645599	2128	1.00	rs10051765	1.00
rs10051765	*SLC34A1*	5	176799992	2022	1.00	rs10051765	1.00
rs12592697	*CYP19A1*	15	51525173	2441	0.71	rs2663546	0.02
						rs2663546	0.02
						rs1147135	0.02
						rs2663543	0.02
						rs2614782	0.02
						rs2663545	0.02
						rs1147136	0.02
						rs2663544	0.02
						rs12902894	0.02
						rs2619688	0.02
						rs2663541	0.01
						rs1147131	0.01
						rs1147133	0.01
						rs2663538	0.01
						rs2663537	0.01
						rs2619693	0.01
						rs2614789	0.01
						rs2614784	0.01
						rs3214932	0.01
						rs1147130	0.01
						rs12908516	0.01
						rs2249535	0.01

						rs12902899	0.01
						rs7494977	0.01
						rs7177267	0.01
						rs7181997	0.01
						rs7168385	0.01
						rs2414080	0.01
						rs4534781	0.01
						rs2663553	0.01
						rs2414079	0.01
						rs12905611	0.01
						rs7171085	0.01
						rs2619694	0.01
						rs2614776	0.01
						rs1147129	0.01
						rs7168846	0.01
						rs2663554	0.01
						rs2414081	0.01
						rs8029155	0.01
						rs12916407	0.01
						rs2663534	0.01
						rs8029369	0.01
						rs2614780	0.01
						rs12904125	0.01
						rs12906866	0.01
						rs2614794	0.01
						rs3784303	0.01
						rs2663533	0.01
						rs7182957	0.01
						rs2614779	0.01
						rs7180258	0.01
						rs7169888	0.01
						rs8031702	0.01
						rs2614790	0.01
						rs1147139	0.01
						rs4438254	0.01
						rs8024428	0.01
						rs2127366	0.01
						rs2170011	0.01
						rs2663535	0.01
						rs2127367	0.01
						rs2663555	0.01
						rs2614781	0.01

CHR= chromosome; POS= position based on NCBI Genome Build 37 (hg19); N SNPs= number of SNPs; PP H4= posterior probability of H4 (full colocalization); SNP PP= posterior probability explained by SNP

Supplementary Table S12: Variants predicted to increase risk of kidney stones via effects on serum mineral metabolism.

Lead variant from mineral metabolism GWAS	CHR	POS	Outcome dataset	PP	Regional probability	Candidate variant	SNP PP	Gene of candidate variant
rs838705	2	234273242	Meta-analysis	1.00	1.00	rs838717	1.00	*DGKD*
			UK Biobank	1.00	1.00	rs838717	1.00	
			FinnGen	1.00	1.00	rs838717	1.00	
rs838717	2	234296444	Meta-analysis	1.00	1.00	rs838717	1.00	*DGKD*
			UK Biobank	1.00	1.00	rs838717	1.00	
			FinnGen	1.00	1.00	rs838717	1.00	
rs838718	2	234296650	Meta-analysis	1.00	1.00	rs838717	1.00	*DGKD*
			UK Biobank	1.00	1.00	rs838717	1.00	
			FinnGen	1.00	1.00	rs838717	1.00	
rs12152922	5	176645599	Meta-analysis	1.00	1.00	rs10051765	1.00	*SLC34A1*
			UK Biobank	1.00	1.00	rs10051765	1.00	
			FinnGen	1.00	1.00	rs10051765	1.00	
rs10051765	5	176799992	Meta-analysis	1.00	1.00	rs10051765	1.00	*SLC34A1*
			UK Biobank	1.00	1.00	rs10051765	1.00	
			FinnGen	1.00	1.00	rs10051765	1.00	
rs4782358	16	88485506	Meta-analysis	0.82	0.82	rs12918968	0.84	*ZFPM1*
			FinnGen	0.82	0.82	rs12918968	0.77	
rs12447180	16	88517722	Meta-analysis	0.82	0.82	rs12918968	0.84	*ZFPM1*
			FinnGen	0.82	0.82	rs12918968	0.77	
rs3091842	20	39344272	Meta-analysis	0.98	0.98	rs3091842	1.00	*MAFB*
			FinnGen	0.96	0.96	rs3091842	1.00	
rs2425431	20	39352714	Meta-analysis	0.98	0.98	rs3091842	1.00	*MAFB*
			FinnGen	0.96	0.96	rs3091842	1.00	
rs209961	20	52715154	Meta-analysis	1.00	1.00	rs6127099	1.00	*CYP24A1*
			UK Biobank	1.00	1.00	rs6127099	1.00	
			FinnGen	1.00	1.00	rs6127099	1.00	
rs2585442	20	52737123	Meta-analysis	1.00	1.00	rs6127099	1.00	*CYP24A1*
			UK Biobank	1.00	1.00	rs6127099	1.00	
			FinnGen	1.00	1.00	rs6127099	1.00	
rs117268564	20	52738434	Meta-analysis	1.00	1.00	rs6127099	1.00	*CYP24A1*
			UK Biobank	1.00	1.00	rs6127099	1.00	
			FinnGen	1.00	1.00	rs6127099	1.00	
rs35194449	20	52742047	Meta-analysis	1.00	1.00	rs6127099	1.00	*CYP24A1*
			UK Biobank	1.00	1.00	rs6127099	1.00	
			FinnGen	1.00	1.00	rs6127099	1.00	

rs2762943	20	52790786	Meta-analysis	1.00	1.00	rs6127099	1.00	*CYP24A1*
			UK Biobank	1.00	1.00	rs6127099	1.00	
			FinnGen	1.00	1.00	rs6127099	1.00	

CHR= chromosome; POS= position based on NCBI Genome Build 37 (hg19); PP= posterior probability of full colocalization; SNP PP= posterior probability explained by single nucleotide polymorphism (SNP); KSD= kidney stone disease; PTH=parathyroid hormone.

Supplementary Table S13: Associations of variants predicted to cause kidney stones with kidney stone disease and mineral metabolism traits.

Candidate causal variant	CHR	POS	Gene	EA	NEA	EAF	KSD (Meta-analysis)		KSD (UK Biobank)		KSD (FinnGenR12)		Adjusted calcium (UK Biobank)		Phosphate (UK Biobank)		PTH (Robinson-Cohen meta-analysis)	
							OR (95% CI)	P	OR (95% CI)	P	OR (95% CI)	P	Beta (SE)	P	Beta (SE)	P	Beta (SE)	P
rs838717	2	234296444	*DGKD*	G	A	0.43	1.10 (1.08-1.12)	$3.08x10^{-24}$	1.10 (1.07-1.13)	$2.60x10^{-11}$	1.10 (1.07-1.13)	$1.71x10^{-14}$	0.05 (0.002)	$1.40x10^{-94}$	-0.03 (0.002)	$7.20x10^{-36}$	0.02 (0.004)	$4.25x10^{-6}$
rs10051765	5	176799992	*SLC34A1*	C	T	0.33	1.14 (1.12-1.16)	$6.45x10^{-43}$	1.16 (1.13-1.19)	$1.30x10^{-24}$	1.13 (1.10-1.15)	$1.94x10^{-20}$	0.03 (0.003)	$5.30x10^{-22}$	-0.05 (0.003)	$9.80x10^{-90}$	-0.04 (0.005)	$2.34x10^{-14}$
rs12918968	16	88520452	*ZFPM1*	C	A	0.44	1.05 (1.04-1.07)	$1.37x10^{-8}$	1.04 (1.01-1.07)	$3.50x10^{-3}$	1.07 (1.04-1.09)	$4.61x10^{-7}$	-0.04 (0.002)	$5.20x10^{-56}$	0.02 (0.002)	$4.00x10^{-10}$	0.01 (0.004)	$1.17x10^{-3}$
rs3091842	20	39344272	*MAFB*	A	G	0.08	1.07 (1.04-1.11)	$9.24x10^{-5}$	1.06 (1.00-1.14)	0.06	1.08 (1.03-1.13)	$5.53x10^{-4}$	0.11 (0.006)	$2.90x10^{-72}$	-0.05 (0.006)	$2.60x10^{-17}$	0.04 (0.010)	$1.59x10^{-4}$
rs6127099	20	52731402	*CYP24A1*	A	T	0.72	1.16 (1.13-1.18)	$2.06x10^{-42}$	1.14 (1.10-1.17)	$1.60x10^{-16}$	1.17 (1.14-1.21)	$4.22x10^{-28}$	0.06 (0.003)	$7.60x10^{-97}$	0.02 (0.003)	$1.40x10^{-13}$	-0.07 (0.005)	$1.01x10^{-44}$

CHR= chromosome; POS= position based on NCBI Genome Build 37 (hg19); EA= effect allele; NEA=non effect allele; EAF= effect allele frequency; KSD= kidney stone disease; P= p-value;

Supplementary Table S14: Associations of variants predicted to cause kidney stones with kidney stone disease and mineral metabolism traits in DiscovEHR.

Variant	Effect allele	Gene	Kidney stone disease prevalence		Serum calcium		Serum phosphate	
			OR	p	Beta	p	Beta	p
rs838717	G	*DGKD*	1.06 (1.04-1.09)	<0.0001	0.02	<0.0001	-0.02	<0.0001
rs10051765	C	*SLC34A1*	1.10 (1.08-1.12)	<0.0001	0.01	<0.0001	-0.04	<0.0001
rs6127099	A	*CYP24A1*	1.05 (1.03-1.08)	<0.0001	0.02	<0.0001	0.01	0.02
All	*G, A, C*	*DGKD, CYP24A1, SLC34A1*	1.07 (1.06-1.09)	<0.0001	0.01	<0.0001	-	-

Mean serum calcium and phosphate are adjusted for kidney stone disease case status. Odds ratio (OR) and beta reflect increased odds of kidney stone disease or change in biochemical measurement, respectively, with addition of one allele. Associations of combinations of *DGKD-, CYP24A1,* and *SLC34A1* risk alleles (All) were not assessed (-) for serum phosphate due to a lack of directional concordance.

Supplementary Table S15: Kidney stone population attributable risk and population attributable fraction in the UK Biobank.

	Risk allele carrier	No risk allele	Total kidney stone prevalence	Kidney stone prevalence non-carriers	Kidney stone prevalence carriers	PAR	PAF (%)
DGKD			0.023	0.021	0.024	$2.20x10^{-3}$	9.43
rs838717	GG/GA	AA					
Case	7994	3365					
No stones	319985	156051					
SLC34A1							
rs10051765	CC/CT	TT	0.023	0.022	0.025	$1.63 x10^{-3}$	7.01
Case	6709	4650					
No stones	266130	209906					
CYP24A1							
rs6127099	AA/AT	TT	0.023	0.020	0.024	$3.56 x10^{-3}$	15.29
Case	10610	749					
No stones	438844	37192					
DGKD, CYP24A1, SLC34A1							
All	1+ risk allele	No risk alleles	0.023	0.019	0.023	$4.42x10^{-3}$	18.95
Case	11256	103					
No stones	470686	5350					

PAR= Population attributable risk (calculated as prevalence in total population- prevalence in individuals without risk allele); PAF= Population attributable fraction (calculated as (prevalence in total population- prevalence in individuals without risk allele)/ prevalence in total population)

Supplementary Table S16: Kidney stone population attributable risk and population attributable fraction in DiscovEHR.

	Risk allele carrier	No risk allele	Total kidney stone prevalence	Kidney stone prevalence non-carriers	Kidney stone prevalence carriers	PAR	PAF (%)
DGKD			0.113	0.106	0.116	$6.12x10^{-3}$	5.44
rs838717	GG/GA	AA					
Case	13121	5839					
No stones	100427	49000					
SLC34A1							
rs10051765	CC/CT	TT	0.113	0.107	0.117	$6.08x10^{-3}$	5.40
Case	11357	7603					
No stones	85653	63774					
CYP24A1							
rs6127099,	AA/AT	TT	0.113	0.108	0.113	$4.23x10^{-3}$	3.76
Case	17454	1506					
No stones	137036	12391					
DGKD, CYP24A1, SLC34A1							
All	1+ risk allele	No risk alleles	0.113	0.100	0.113	$1.26x10^{-2}$	11.23
Case	18767	193					
No stones	147689	1738					

PAR= Population attributable risk (calculated as prevalence in total population- prevalence in individuals without risk allele); PAF= Population attributable fraction (calculated as (prevalence in total population- prevalence in individuals without risk allele)/ prevalence in total population).

Supplementary Table S17. Drug target Mendelian randomization; effects of modulating mineral metabolism traits on odds of kidney stone disease.

Analysis					MR IVW		Heterogeneity			Intercept	
Gene	r^2	Mean R^2 (SD)	Outcome	N SNPs	OR (95% CI)	FDR-P	Q	Q_df	P	Intercept β	P
CaSR-signaling: effects of decreasing albumin-adjusted serum calcium concentration											
DGKD	0.1	3.27x10-4 (3.77x10-4)	Meta-analysis	10	0.22 (0.17-0.30)	2.74x10-24	14.00	9	0.12	4.71x10-3 (0.01)	0.62
			UK Biobank	9	0.26 (0.17-0.38)	4.50x10-11	10.76	8	0.22	0.03 (0.02)	0.24
			FinnGen	11	0.20 (0.14-0.30)	1.62x10-16	15.29	10	0.12	-0.04 (0.03)	0.25
CASR	0.01	9.51x10-4 (1.35x10-3)	Meta-analysis	14	0.72 (0.53-0.97)	0.03	78.52	13	2.09x10-11	8.17x10-4 (0.01)	0.94
			UK Biobank	13	0.67 (0.48-0.93)	0.02	41.42	12	4.17x10-5	0.03 (0.03)	0.45
			FinnGen	11	0.76 (0.54-1.08)	0.13	42.58	10	5.91x10-6	-0.04 (0.04)	0.28
	0.1	7.89x10-4 (1.01x10-3)	Meta-analysis	43	0.74 (0.64-0.85)	2.67x10-5	142.07	42	8.59x10-13	0.04 (0.02)	0.06
			UK Biobank	44	0.70 (0.59-0.82)	2.14x10-5	111.10	43	6.17x10-8	0.02 (0.01)	0.15
			FinnGen	37	0.78 (0.67-0.91)	2.41x10-3	88.96	36	2.24x10-6	-3.29x10-4 (0.01)	0.98
Vitamin D metabolism: effects of decreasing albumin-adjusted serum calcium concentration											
CYP24A1	0.01	5.37x10-4 (4.98x10-4)	Meta-analysis	4	0.09 (0.07-0.12)	3.58x10-5	2.86	3	0.41	-3.74x10-3 (0.01)	0.7
			UK Biobank	5	0.12 (0.08-0.19)	3.46x10-5	6.29	4	0.18	0.03 (0.03)	0.37
			FinnGen	5	0.07 (0.05-0.11)	8.75x10-3	6.15	4	0.19	-0.04 (0.04)	0.33
	0.1	3.29x10-4 (3.08x10-4)	Meta-analysis	18	0.10 (0.08-0.12)	3.43x10-124	20.37	17	0.26	0.02 (0.02)	0.31
			UK Biobank	17	0.12 (0.09-0.15)	1.95x10-61	15.74	16	0.47	-5.80x10-4 (0.01)	0.97

			FinnGen	18	0.08 (0.06-1.10)	4.16x10-87	20.64	17	0.24	0.04 (0.01)	4.62x10-3
Phosphate metabolism: effects of increasing serum phosphate concentration											
SLC34A1	0.1	3.24x10-4 (3.95x10-4)	Meta-analysis	8	0.08 (0.06-0.11)	1.09x10-7	2.54	7	0.92	0.03 (0.01)	0.04
			UK Biobank	8	0.07 (0.04-0.10)	6.57x10-2	4.38	7	0.74	0.01 (0.01)	0.34
			FinnGen	7	0.10 (0.07-0.15)	5.71x10-3	1.44	6	0.96	0.02 (0.01)	0.04

CHR= chromosome; End= end position; FDR-P= P-value adjusted for 5% false discovery rate; IVW = inverse-variance weighted, N SNPs= number of SNPs; P= p--value; Q= Q statistic; Q_df= Q degrees of freedom; r^2= linkage disequilibrium threshold; R^2 = variance in exposure explained by instrumental variable; SD= standard deviation; SE=standard error

Supplementary Table S18: Rare predicted deleterious *DGKD* missense variants in 100kGP participants with kidney stone disease.

Variable	Normal Range	Proband 1	Proband 2	Proband 3
DGKD variant	-	H190Q	I221N	R1181W
DGKD variant ID		2:233434885:C:G	2:233435893:T:A	2:233468539:C:T
Sex	-	Female	Female	Male
Stone analysis	-	UK	UK	Calcium oxalate 70%, calcium phosphate 30%
Adjusted serum calcium (mmol/l)	2.10-2.50	UK	UK	2.45
Serum phosphate (mmol/l)	0.70-1.40	UK	UK	1.14
24-hour urinary calcium excretion (mmol)	>5	UK	UK	8.9
24-hour urinary pH	-	UK	UK	6.86
24-hour urinary phosphate excretion (mmol)	<35	UK	UK	29.6
24-hour urinary oxalate excretion (umol)	<460	UK	UK	273
24-hour urinary citrate excretion (mmol)	>2.5	UK	UK	2.39
24-hour urinary magnesium excretion (mmol)	>3	UK	UK	5.2
24-hour urinary uric acid excretion (umol)	200-430 males 140-360 females	UK	UK	400
Family history of kidney stones	-	UK	Yes	Yes
Additional phenotypes	-	Hyperparathyroidism	-	-
Allele frequency 100KGP*	-	$9.4x10^{-5}$	$3.9x10^{-5}$	$6.7x10^{-4}$
Allele frequency DiscovEHR	-	$1.4x10^{-4}$	-	$1.9x10^{-3}$
Allele frequency Gnomad	-	$5.0x10^{-5}$	-	$6.0x10^{-4}$
CADD	-	11	24	32
SIFT	-	0.02	0	0
PolyPhen	-	0.768	0.987	1

*Frequency in rare diseases HG38 cohort; UK unknown.

Supplementary Table S19: Rare *DGKD* missense variants associated with kidney stone disease in the DiscovEHR cohort.

DGKD Variant	Variant ID	Controls n (%)	Stone cases n (%)	P value	CADD	SIFT	Polyphen	Allele frequency Gnomad	Allele frequency 100kGP*	Allele frequency DiscovEHR
Ile91Val	2:233390406:A:G	14 (0.01)	5 (0.03)	0.049	21	0.01	0.102	3.5x10-5	-	$1.4x10^{-4}$
Thr319Ala	2:233438249:A:G	1 (<0.1)	2 (0.01)	0.033	20	0.26	0.00	-	-	$2.2x10^{-5}$
Val464Ile	2:233446767:G:A	25 (0.02)	8 (0.06)	0.023	22	0.03	0.003	1.1x10-4	7.1x10-5	$2.4x10^{-4}$
Arg900His	2:233459761:G:A	5 (<0.01)	3 (0.02)	0.048	27	0	0.954	2.8x10-5	-	$5.9x10^{-5}$

*Frequency in rare diseases HG38 cohort.

Bibliography

1. Howles SA, et al. Genetic variants of calcium and vitamin D metabolism in kidney stone disease. *Nat Commun*. 2019;10(1):5175.

2. Varadi M, et al. AlphaFold Protein Structure Database: massively expanding the structural coverage of protein-sequence space with high-accuracy models. *Nucleic Acids Res*. 2022;50(D1):D439–D444.

3. Jumper J, et al. Highly accurate protein structure prediction with AlphaFold. *Nature*. 2021;596(7873):583–589.

4. Ferguson KM, et al. Structure of the high affinity complex of inositol trisphosphate with a phospholipase C pleckstrin homology domain. *Cell*. 1995;83(6):1037–1046.
