# Supplementary material for: Genetic variants predisposing to an increased risk of kidney stone disease
Source: J Clin Invest. 2025 May 15;135(15):e186915. doi: 10.1172/JCI186915 (PMC12321396; doi:10.1172/JCI186915)

Full unedited gel for Figure S8a and b Tubulin 2024-03-25 17h46m21s(Chemiluminescence)

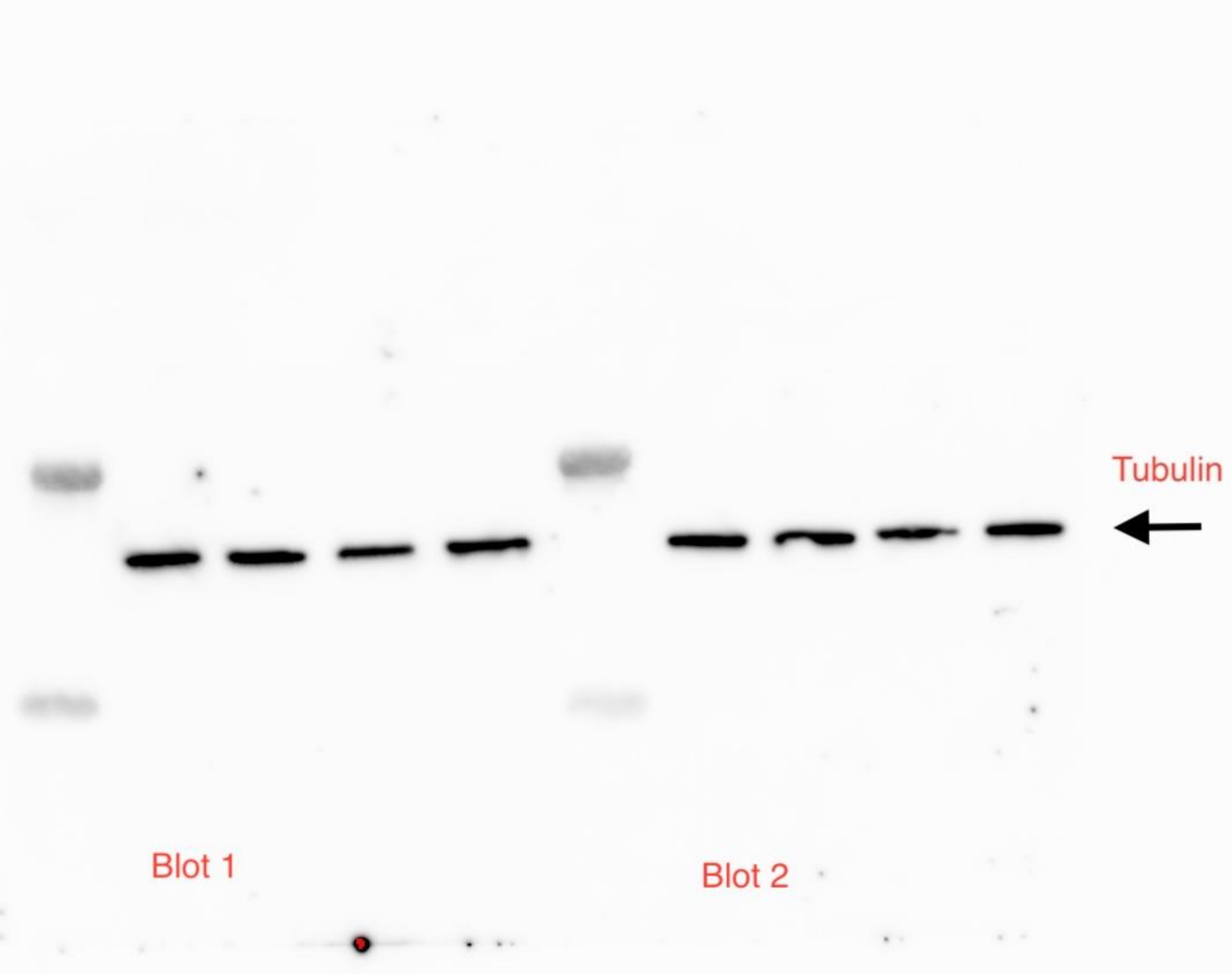

Full unedited gel for Figure S8a and b Tubulin Antibody 2024-03-28 13h56m52s(Composite)

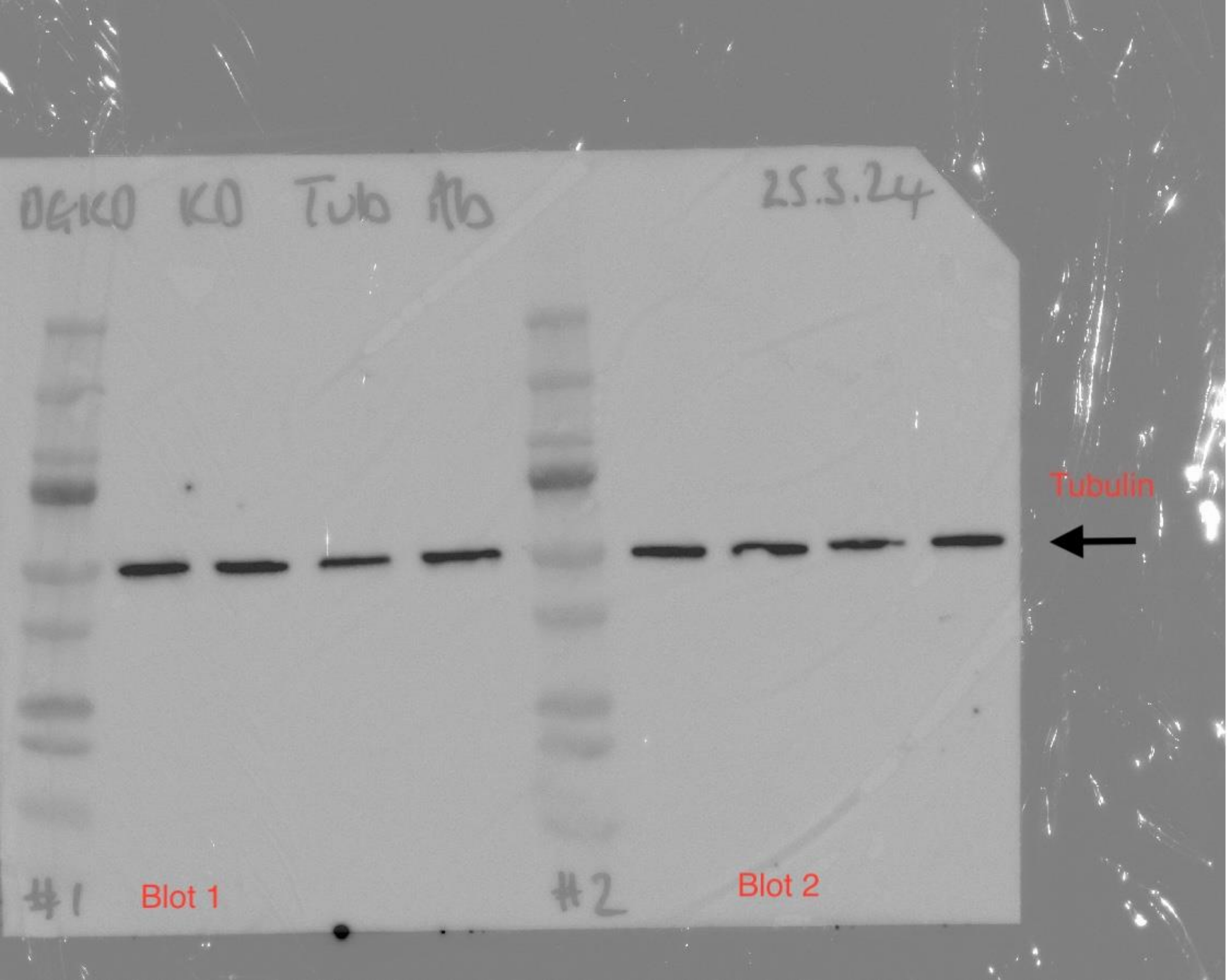

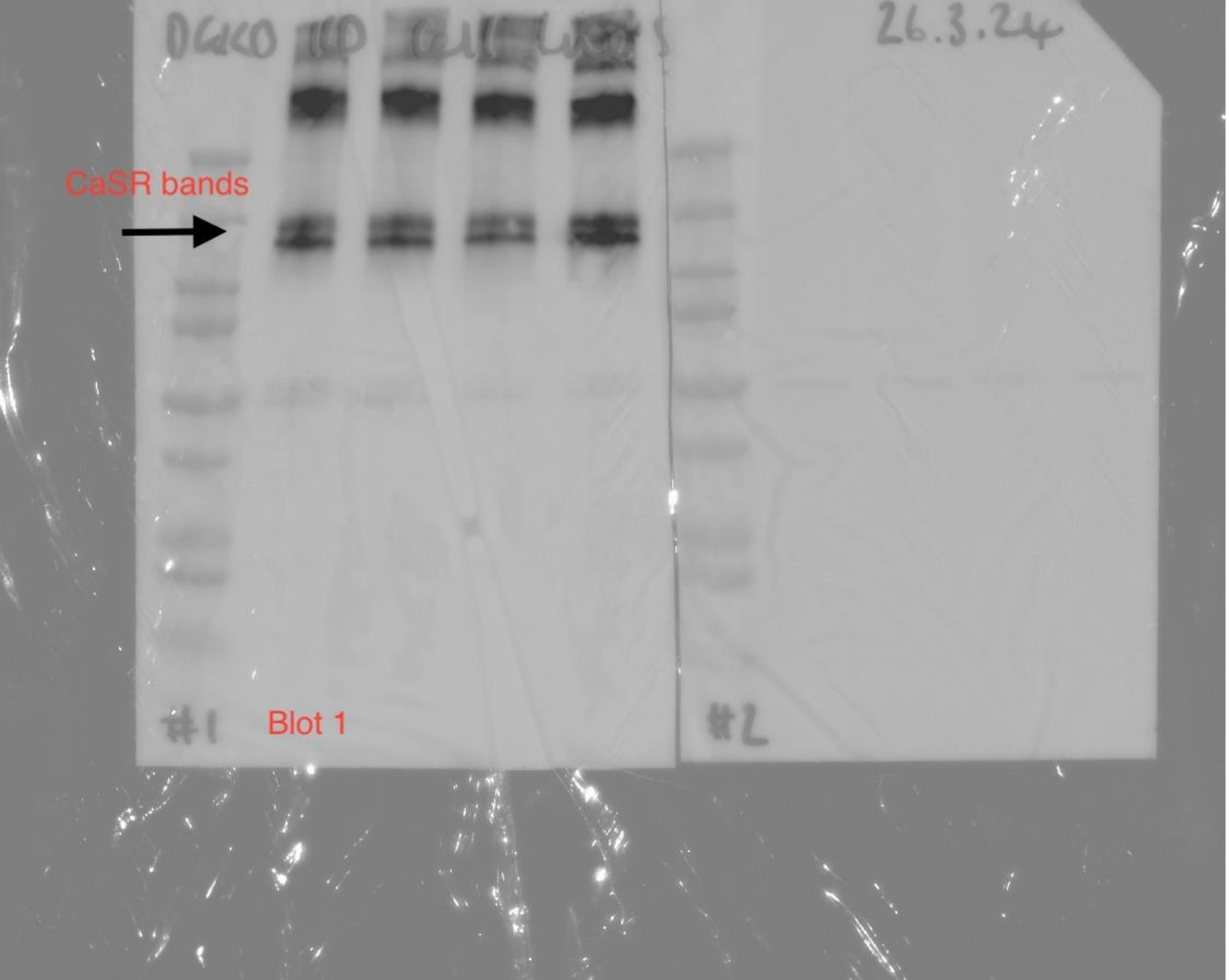

Full unedited gel for Figure S8a CaSR Antibody 2024-03-28 13h58m16s(Composite)

Full unedited gel for Figure S8ai CaSR 2024-02-19 14h20m15s(Chemiluminescence)

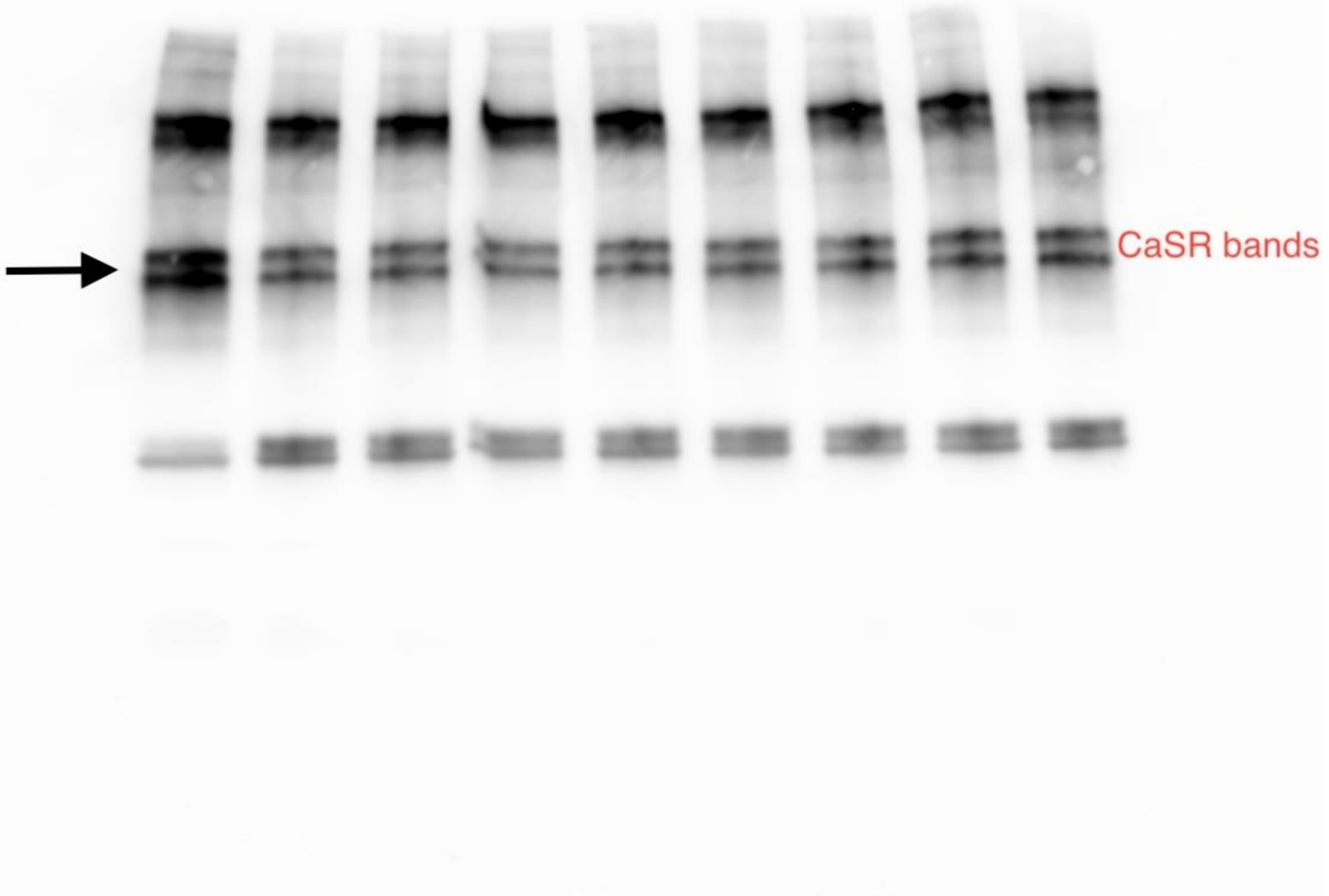

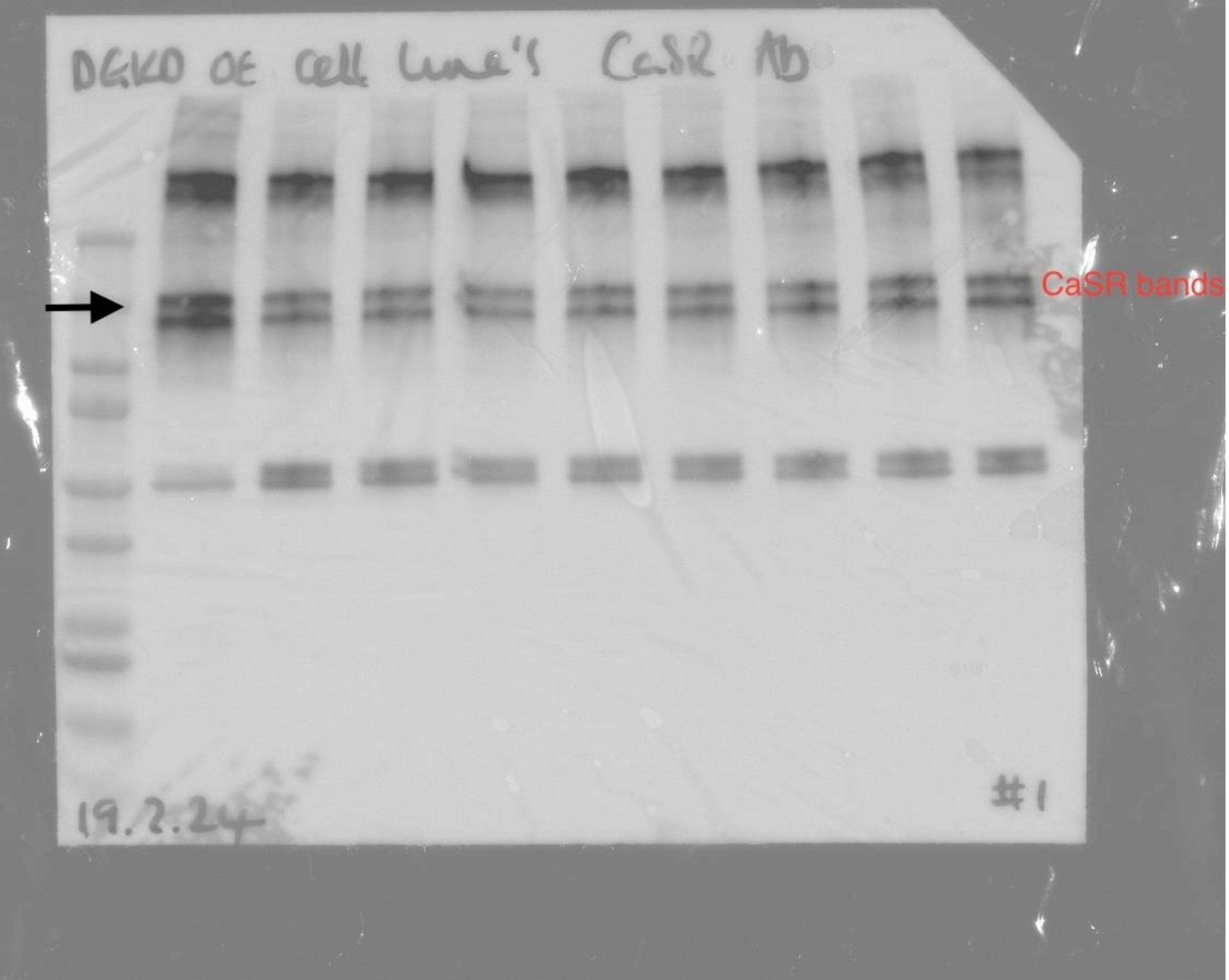

Full unedited gel for Figure S8ai CaSR 2024-02-19 14h21m14s(Composite)

Full unedited gel for Figure S8ai CaSR 2024-03-26 15h30m37s(Chemiluminescence)

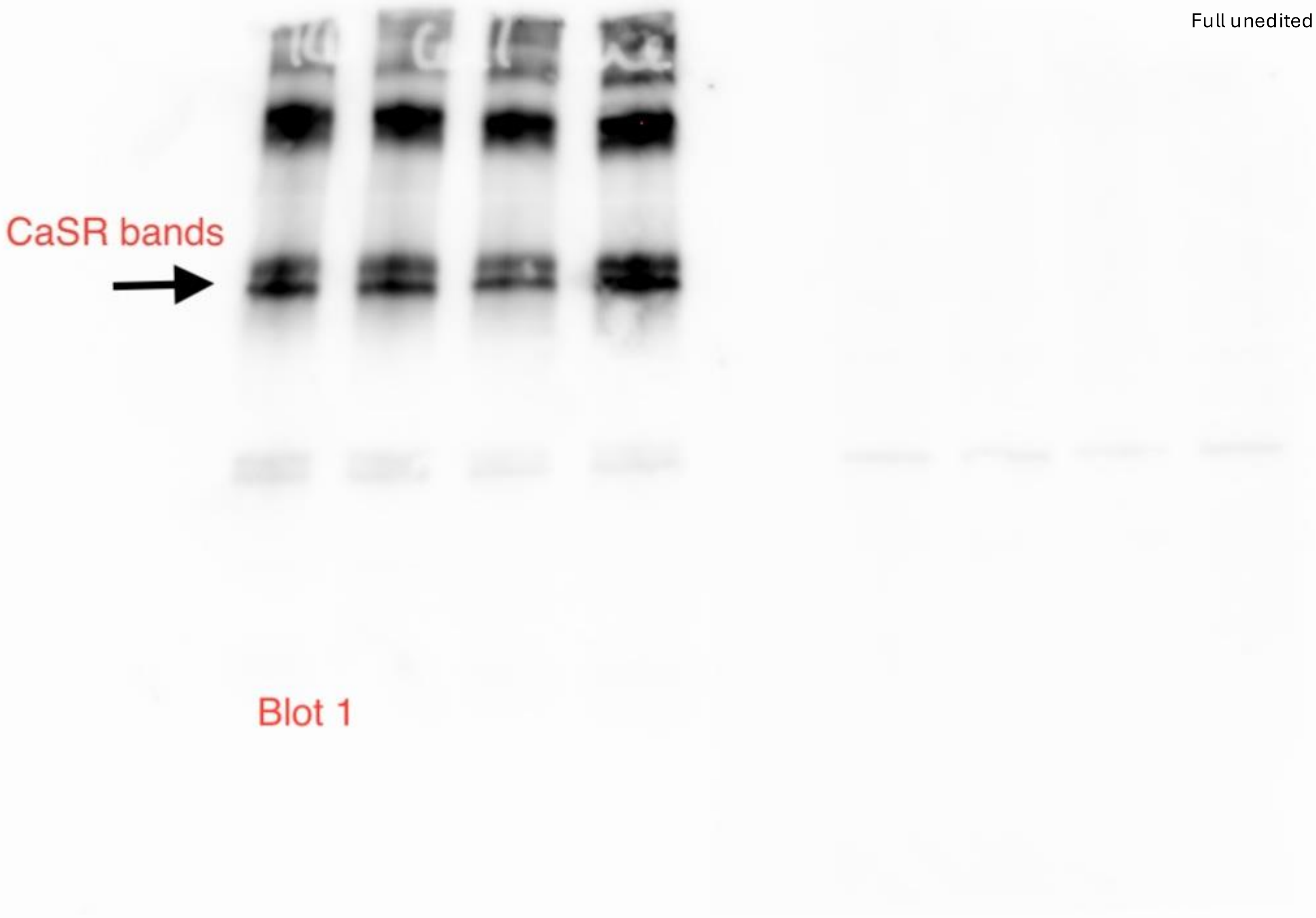

Full unedited gel for Figure S8ai Tubulin 2024-02-16 14h24m28s(Chemiluminescence)

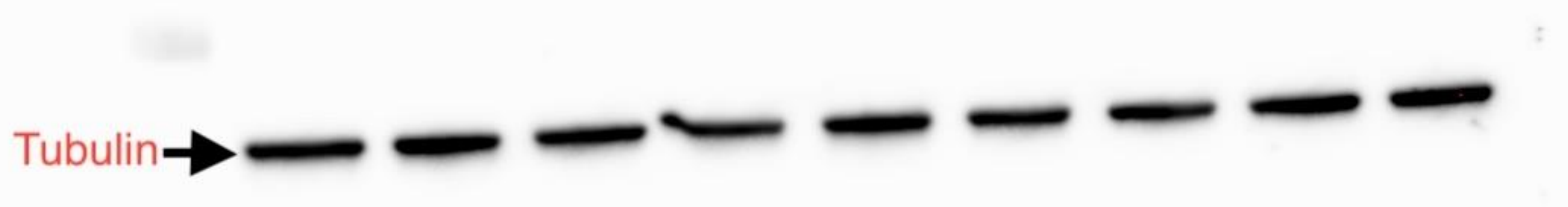

Full unedited gel for Figure S8ai Tubulin 2024-02-16 14h26m17s(Composite)

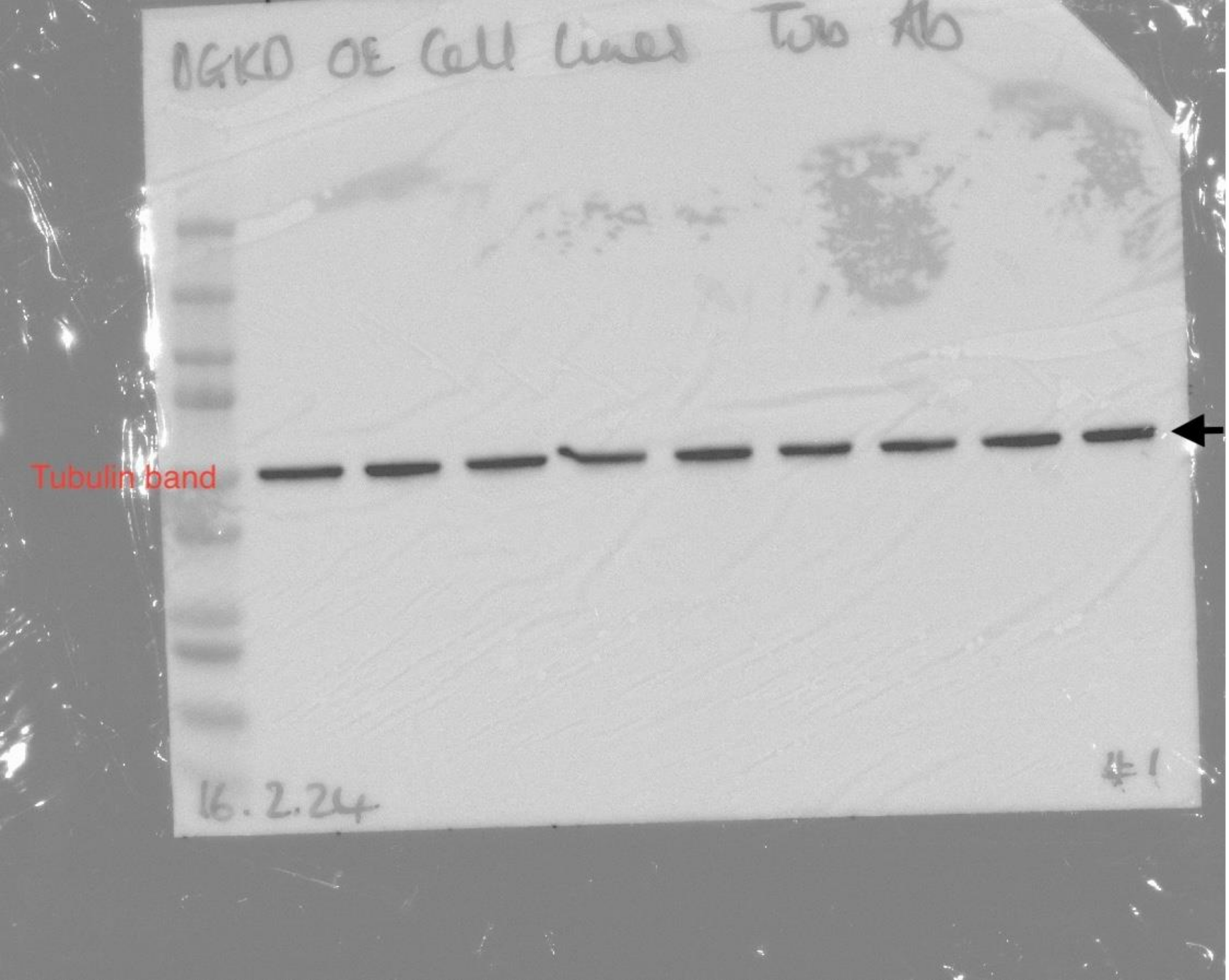

Full unedited gel for Figure S8aii DGKD Myc 2024-02-19 12h38m48s(Chemiluminescence)

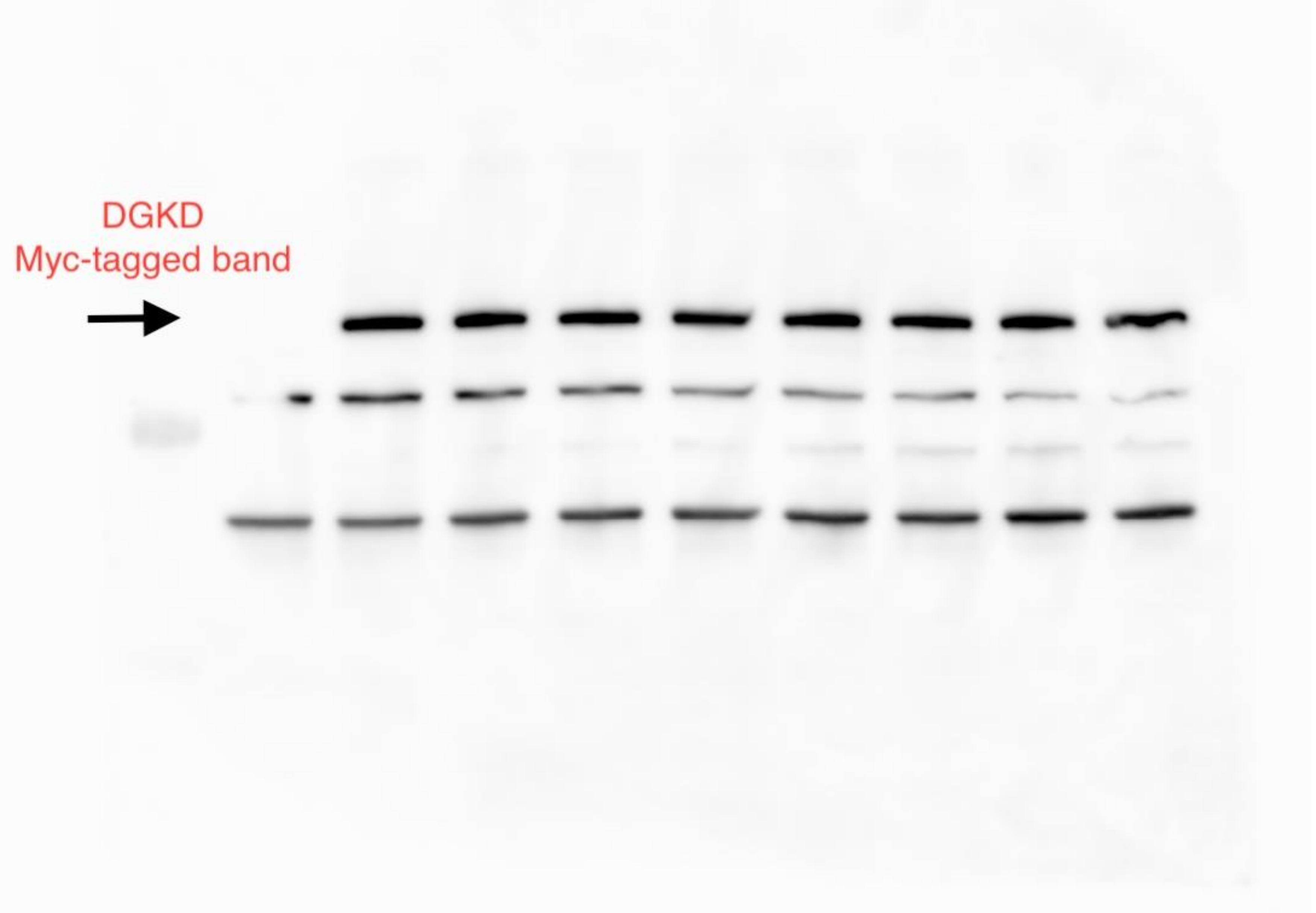

Full unedited gel for Figure S8aii DGKDMyc 2024-02-19 12h40m11s(Composite)

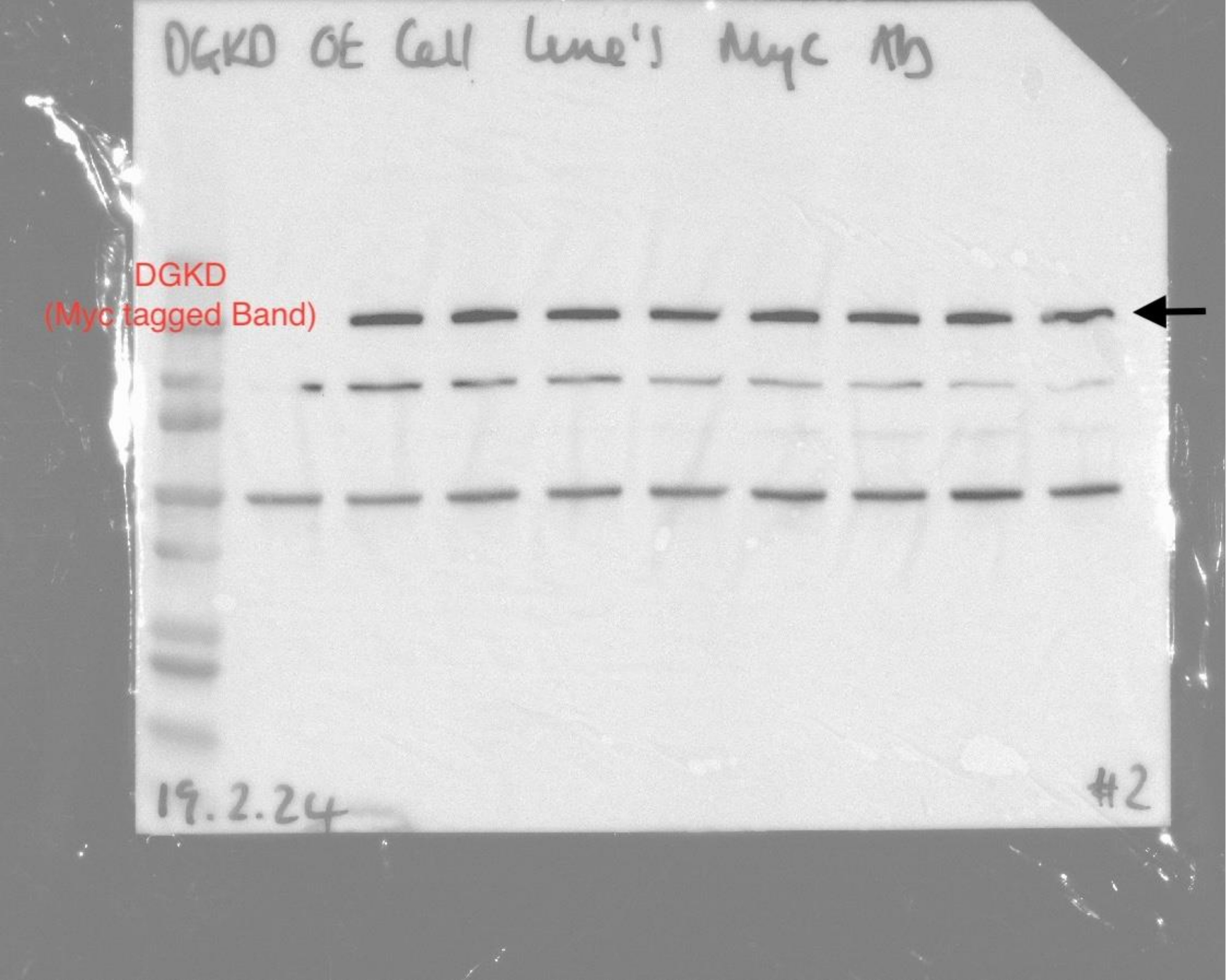

Full unedited gel for Figure S8b DGKD 2024-03-28 14h36m07s(Chemiluminescence)

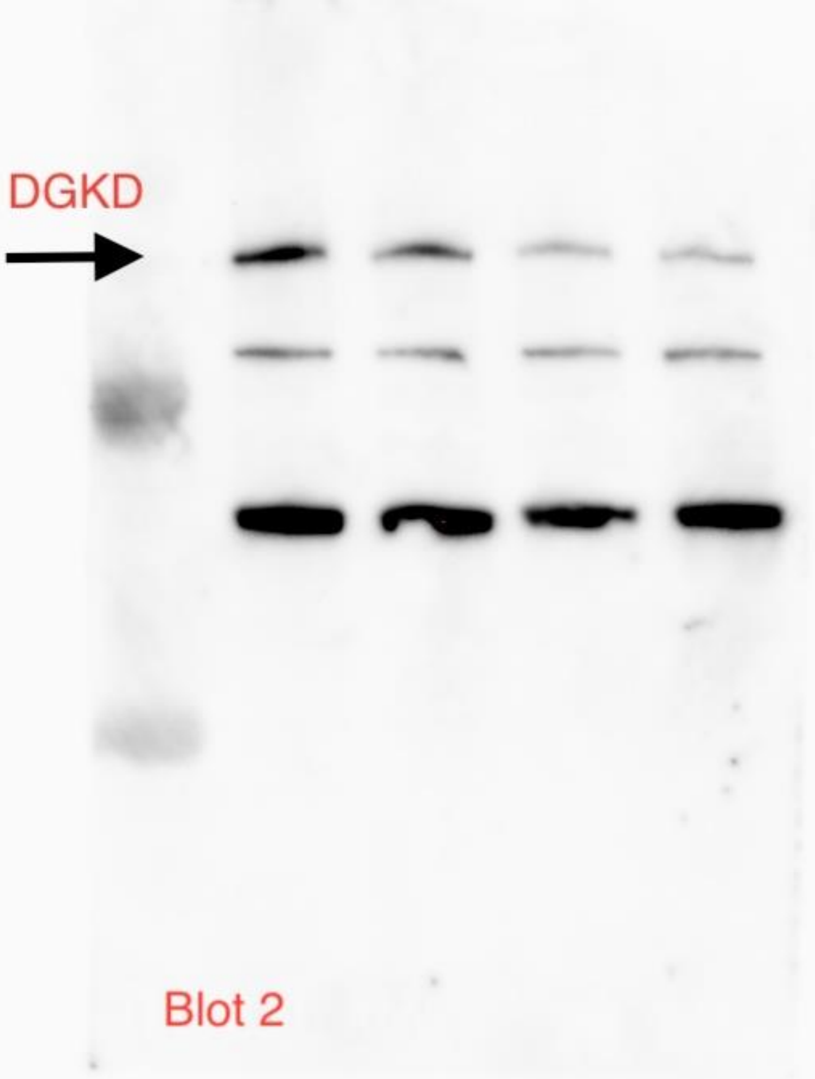

Full unedited gel for Figure S8b DGKD Antibody 2024-03-28 14h37m06s(Composite)

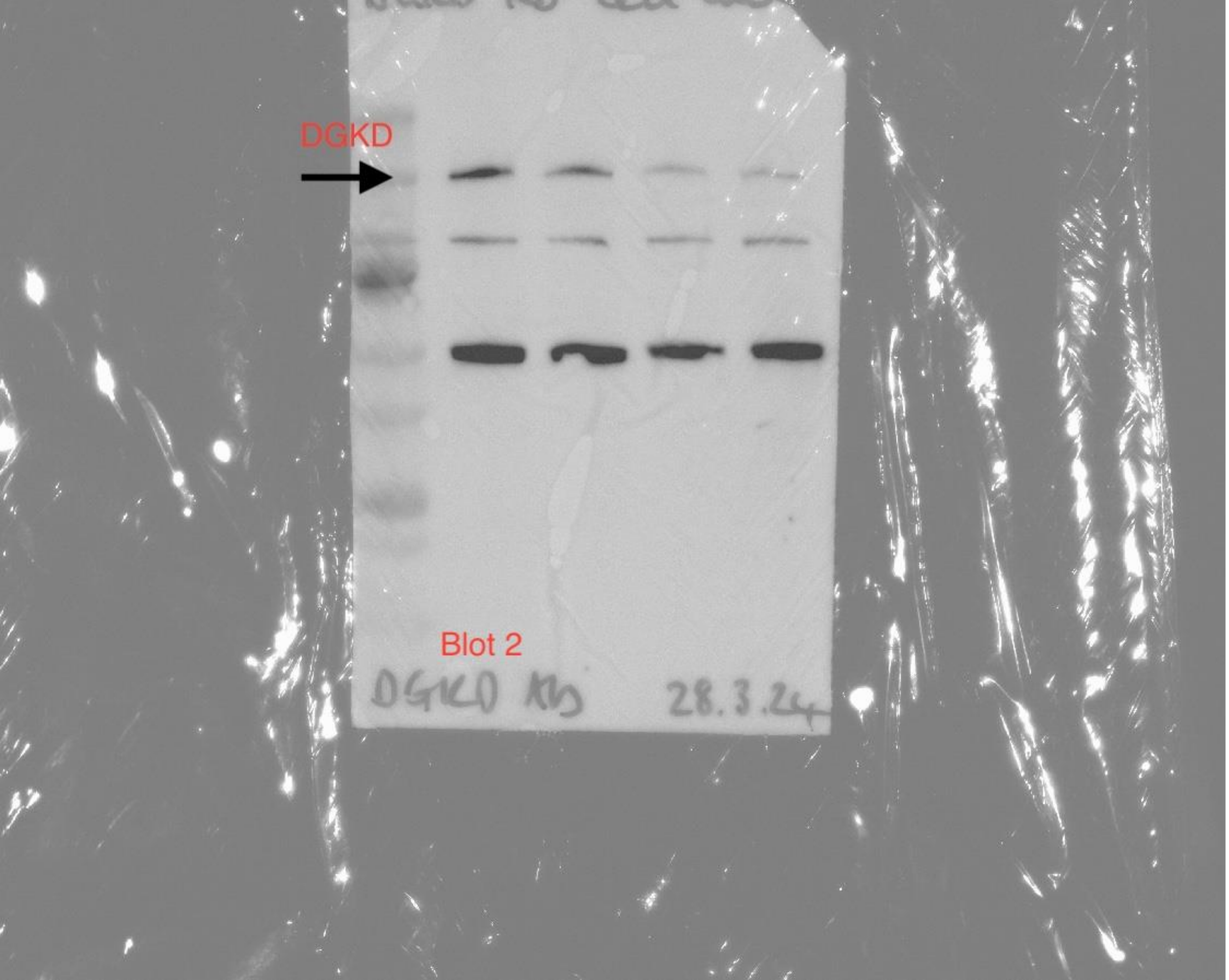

Full unedited gel for Figure S8b Tubulin 2024-02-16 14h31m51s(Chemiluminescence)

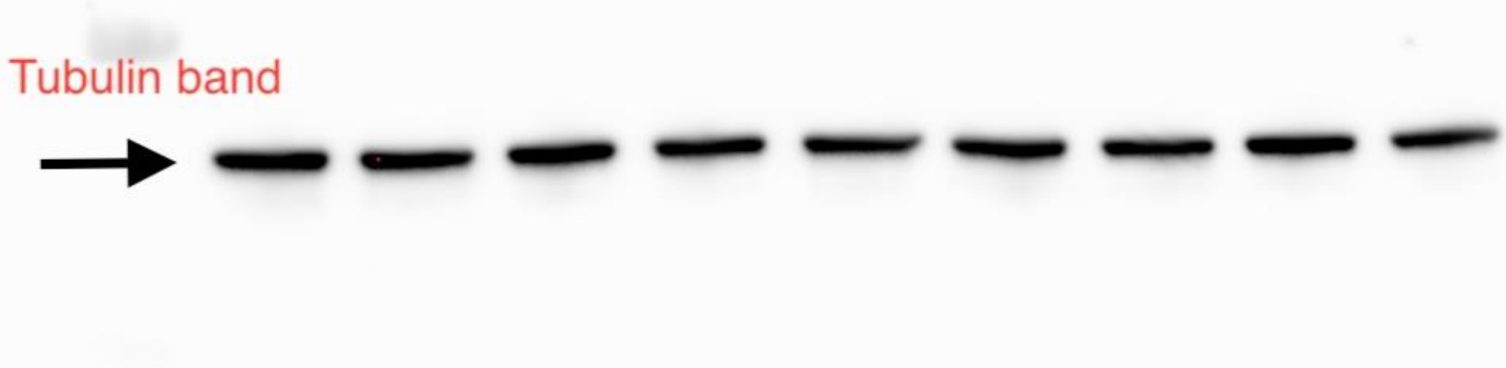

Full unedited gel for Figure S8b Tubulin 2024-02-16 14h32m41s(Composite)

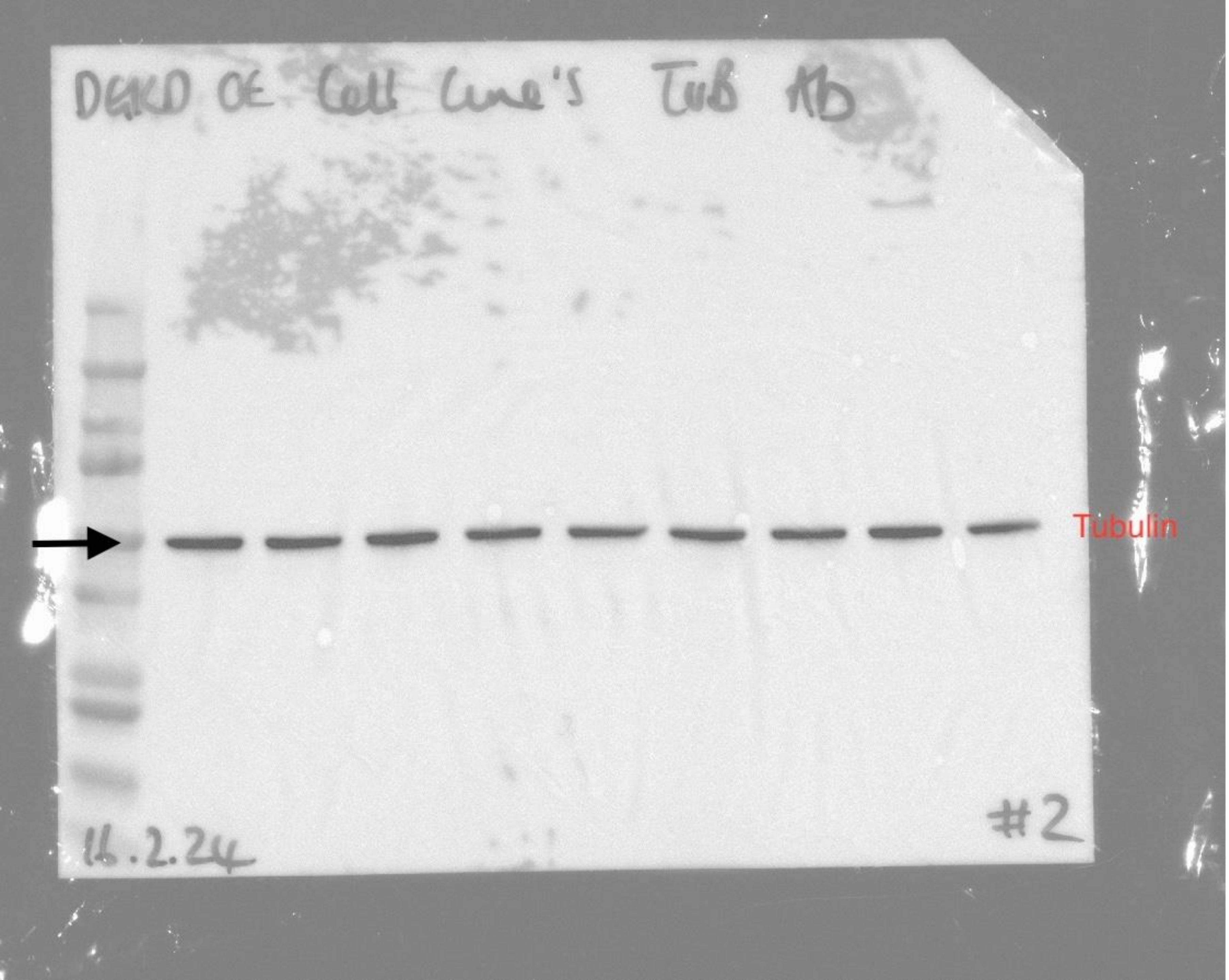

Supplement: Unedited blot and gel images [file jci-135-186915-s123.pdf]
